# Exploring Human Milk Dynamics: Interindividual Variation in Milk Proteome, Peptidome, and Metabolome

**DOI:** 10.1021/acs.jproteome.1c00879

**Published:** 2022-02-01

**Authors:** Pieter M. Dekker, Sjef Boeren, Johannes B. van Goudoever, Jacques J. M. Vervoort, Kasper A. Hettinga

**Affiliations:** † Food Quality and Design Group, Wageningen University & Research, Bornse Weilanden 9, 6708 WG Wageningen, The Netherlands; ‡ Laboratory of Biochemistry, Wageningen University & Research, Stippeneng 4, 6708 WE Wageningen, The Netherlands; § Department of Pediatrics, Amsterdam UMC Vrije Universiteit Emma Children’s Hospital, 1081 Amsterdam, The Netherlands

**Keywords:** breast milk, proteomics, peptidomics, metabolomics, proteins, peptides, metabolites, variability

## Abstract

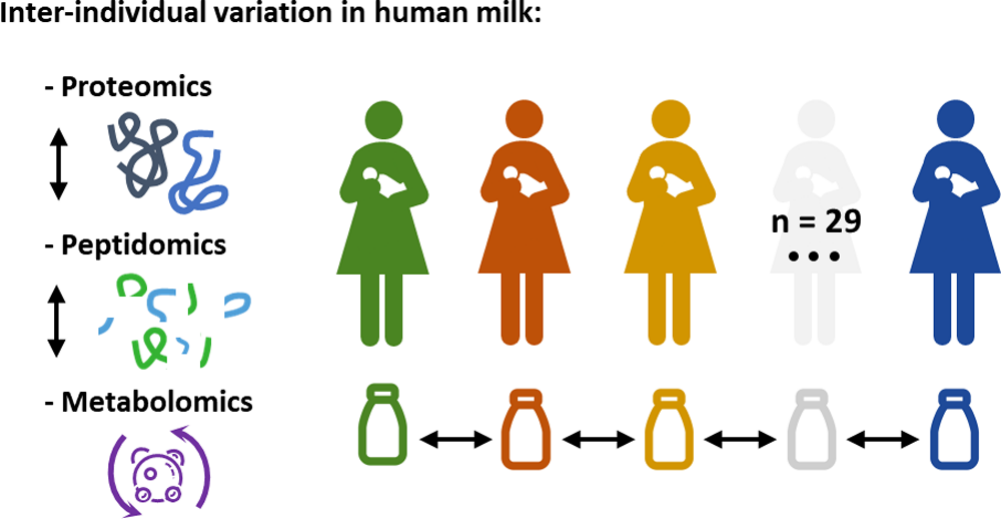

Human milk is a dynamic biofluid, and its detailed composition receives increasing attention. While most studies focus on changes over time or differences between maternal characteristics, interindividual variation receives little attention. Nevertheless, a comprehensive insight into this can help interpret human milk studies and help human milk banks provide targeted milk for recipients. This study aimed to map interindividual variation in the human milk proteome, peptidome, and metabolome and to investigate possible explanations for this variation. A set of 286 milk samples was collected from 29 mothers in the third month postpartum. Samples were pooled per mother, and proteins, peptides, and metabolites were analyzed. A substantial coefficient of variation (>100%) was observed for 4.6% and 36.2% of the proteins and peptides, respectively. In addition, using weighted correlation network analysis (WGCNA), 5 protein and 11 peptide clusters were obtained, showing distinct characteristics. With this, several associations were found between the different data sets and with specific sample characteristics. This study provides insight into the dynamics of human milk protein, peptide, and metabolite composition. In addition, it will support future studies that evaluate the effect size of a parameter of interest by enabling a comparison with natural variability.

## Introduction

Human milk is a dynamic biofluid. Its composition depends on, for example, lactation stage and health status of the mother. Proteins are one of the main constituents of human milk and have been shown to be involved in the growth and the healthy development of the infant. To date, the composition of the human milk proteome is well established. The most recent studies on this have reported up to 1500 proteins in human milk.^1^ Part of the proteins in human milk are synthesized in the mammary gland, for instance, caseins and α-lactalbumin. Besides this, a vast number of proteins are transferred into the alveolar lumen from the systemic circulation of the mother.^2^ Among these are for example albumin, immunoglobulin G, and even nonhuman proteins.^3,4^

Already before excretion of the milk, proteolysis of proteins takes place, resulting in the human milk peptidome. This peptidome has been shown to comprise more than 4000 unique peptides.^5^ The majority of these peptides originate from the precursor protein β-casein. The fact that β-casein is overrepresented in the human milk peptidome is first of all due to its abundance. Besides this, its open and flexible structure makes it prone to proteolytic digestion. Other proteins that are abundant in milk, such as α-lactalbumin, have a closed and globular structure, resulting in a lower contribution of these precursor proteins to the peptidome. Within the human milk peptidome, a substantial number of peptides was found to be a bioactive peptide itself or to be a precursor for a bioactive peptide.^6,7^

Researchers have pursued evaluation of the presence of biomarkers in the peptidome or proteome, investigating the relation with factors such as breast cancer risk or maternal allergy.^8,9^ Besides this, many recent proteomics and peptidomics studies on human milk have focused on longitudinal variation.^10−15^ These studies provide the evidence that the human milk proteome changes over lactation according to functionality, that is, from a direct defense mechanism toward the reinforcement for an independent immune system. So far, however, there has been little discussion about interindividual variation in the human milk proteome and peptidome. In the few studies on this done so far, it was established that variation between individual mothers is greater than longitudinal variation. This was observed to be valid for both the human milk proteome^12^ and peptidome.^16^ From this, the questions arise: what is the extent of this interindividual variation and what is its origin? In addition, it emphasizes the challenge in investigating relations between composition and other parameters such as maternal characteristics. If the interindividual variation is not considered in those investigations, the relevance of differences found between groups of samples will be hard to interpret and can easily be overestimated.

Besides the importance of mapping the interindividual variation in the human milk proteome and peptidome, it remains a challenge to understand the mechanisms underlying this interindividual variation. Part of this variation might be explained by biological processes in the human body, of which indicators might be found in low molecular weight substances, that is, metabolites. An example of this could be the relation between, for example, free amino acids and protein synthesis. Nevertheless, both proteomics and peptidomics analyses result in hundreds of features, giving rise to a challenge in turning data into biologically relevant information. Synthesis and secretion of milk proteins are regulated by biological pathways, and proteins can function interactively in different biological pathways. Therefore, protein coexpression networks can provide useful information on protein relationships and involvement in biological pathways.^17^ In recent years, weighted correlation network analysis (WGCNA) has been used to construct and analyze such coexpression networks in proteomics data.^18−20^ Peptides, on the other hand, are intermediates in the proteolytic degradation of proteins. In complex samples, such as human milk, peptides originate from dozens of precursor proteins. Peptide levels can be interdependent due to, for example, partly overlapping sequences (peptide-ladders) or specificity of proteolytic cleavage. Grouping peptides based on correlation in intensities can unveil patterns of proteolytic degradation.^21^ In approaching these complex data, WGCNA can be used to identify clusters of associated proteins and peptides. In short, the goals with this WGCNA approach were (1) to elucidate whether interindividual variation was specific for certain biological functions or pathways, (2) to shed light on protein–protein and peptide–peptide associations, (3) to investigate associations of proteins and peptides with sample characteristics, and (4) to investigate whether protein and peptide intensities were associated with metabolite levels.

In the current study, we investigated the variation in human milk proteome, peptidome, and metabolome in pooled human milk samples from 29 healthy mothers taken in the third month of lactation. Longitudinal variation in the human milk proteome is the largest in the first month, where a transition takes place from colostrum to mature milk. In the third month of lactation, it is known that longitudinal variation due to the maturation of the milk has leveled out.^22,23^ This time point was therefore chosen as a representation of mature human milk. Samples were analyzed, and interindividual variation for all three omics analyses were reported. Furthermore, relations between the three omics data sets were studied using WGCNA to find underlying reasons for the interindividual variation.

## Experimental Section

### Sample Material

Human milk samples were obtained from healthy mothers donating breastmilk to the Dutch Human Milk Bank (Amsterdam, The Netherlands). Donating mothers were subjected to a preliminary screening by the milk bank, and the milk was collected according to standardized procedures (http://www.moedermelkbank.nl). Informed consent was provided by all mothers to use remnants of the donated milk for scientific research.

A selection of 298 samples was made, donated by 30 different mothers, in the third month postpartum. Latter criterion was chosen to avoid influence of the large longitudinal variation present in milk in the first weeks postpartum. Subsequently, samples were pooled per mother. One of these pooled samples was removed from our selection due to its distinct peptide profile in combination with a low fat and carbohydrate content. These observations indicate the occurrence of mastitis; consequently, the sample was considered an outlier. After this removal, the sample set comprised 29 pooled samples from a total of 286 milk samples. The number of samples included in the pooled samples ranged from 5 to 16, with a time range from 2 to 28 days. Milk was obtained by manual or pump expression at home and collected in a polypropylene bottle. After collection, samples were stored immediately at −18 °C. Samples were picked up from homes and transported in a freezer at −20 °C to the milk bank where they were stored at the same temperature. Detailed information on the samples included in this study can be found in Table 1. Fat content in the samples was measured by the Dutch Human Milk Bank as described by De Waard et al.^24^

**Table 1:** Subject Demographics and Sample Characteristics

Infant		
gender	female	14
	male	15
Mother		
BMI	normal	18
	overweight	7
	obese	4
age, years	median	32.4
	range	26–42.8
Milk Samples		
samples included in pool	median	9
	range	5–16
time (days) between first and last sample in pool	median	10
	range	2–28
total protein concentration (mg/mL)	mean	9.4
	range	8.6–10.2

### Proteomics

#### Sample Preparation

Human milk samples were thawed at 4 °C, and skimmed milk was obtained after centrifugation at 1500*g* for 10 min at 10 °C. Skimmed milk was then centrifuged at 100 000*g* for 30 min at 30 °C. Milk serum was collected, and the serum protein concentration was determined in duplicate with the Pierce bicinchonic acid (BCA) assay (Thermo Scientific, Waltham, MA). According to these results, milk serum samples were diluted in 100 mM Tris to a concentration of 1 μg/μL protein. To a 100 μL diluted milk sample, a final concentration of 15 mM dithiothreitol was added and subsequently incubated at 45 °C for 30 min. After disulfide bonds were reduced, the sample was transferred into 6 M urea, and alkylation of the reduced cysteine residues was obtained by addition of 20 mM acrylamide and 10 min incubation at room temperature. From this alkylated protein sample, 180 μL, containing 36 μg of protein, was transferred to a Pall 3K omega filter (10–20 kDa cutoff, OD003C34; Pall, Washington, NY, USA), and the samples were centrifuged at 12 000*g* for 30 min. The filter was washed with a 50 mM ammonium bicarbonate solution. Then 100 μL of 5 ng/μL sequencing grade trypsin was added, and digestion took place overnight under mild shaking at room temperature. The filter with the digested proteins was centrifuged and washed with 100 μL of 1 mL/L formic acid solution. The pH of the final peptide solution was set to around 3 using a 10% trifluoroacetic acid solution.

#### LC–MS/MS

The prepared samples were analyzed with LC–MS/MS as described before.^12^ In short, an LTQ-Orbitrap XL system (Thermo electron, San Jose, CA, USA) was used to obtain full scan FTMS spectra in positive mode (*m*/*z* 380 to 1400). MS/MS scans of the four multiply charged peaks with the highest intensity were recorded in the linear trap in data-dependent mode and with an MS/MS threshold of 5000.

#### Data Analysis (Proteins)

The Andromeda search engine of the MaxQuant software v1.6.1.0 was used to analyze the raw LC–MS/MS data.^25^ A database (*n* = 4296) was used comprising the major human and bovine milk proteins as well as allergen proteins. Detailed information on the creation of this database as well as the database itself can be found in a previous study.^26^ In silico digestion was carried out with trypsin digestion with a maximum of 2 missed cleavages per peptide sequence. Peptide length was set to a minimum of 6 and a maximum of 35 amino acids, and a fixed modification was set to acrylamide on cysteines to account for the alkylation. A false discovery rate (FDR) of 1% was used at the peptide and protein level. Furthermore, a precursor mass tolerance was set to 20 ppm and fragment mass tolerance to 0.5 Da. Recalibration was carried out with a first search using a database with common contaminants.

Further data analysis was carried out, and figures were made using R version 4.0.1.^27^ First, identifications were filtered to exclude matches with the decoy database, potential contaminants, proteins only identified with modified peptides, proteins only identified with one peptide, and proteins identified in less than 10 out of 29 samples. Label-free quantification (LFQ) intensities were used to analyze the data further and were imputed (described below), transformed with logarithm base 10, and corrected for the dilution factor.

Imputation of missing values was carried out with the Gibbs sampler based GSimp algorithm, designed for the imputation of left censored missing values.^28^ For annotation purposes, a leading protein was selected for protein groups with more than one protein. If a protein group included one or more reviewed proteins, the first reviewed protein was selected as leading protein. If no reviewed protein was included, the protein with the most extensive GO annotation was selected as leading protein.

The mass spectrometry proteomics data have been deposited to the ProteomeXchange Consortium via the PRIDE^29^ partner repository with the data set identifier PXD028280.

### Peptidomics

#### Sample Preparation

Human milk samples were thawed at 4 °C, and skimmed milk was obtained after centrifugation at 1500*g* for 10 min at 10 °C. Proteins were precipitated by addition of an equal volume of 200 g/L trichloroacetic acid in milli-Q water and subsequent centrifugation at 3000*g* for 10 min at 4 °C. From the resulting supernatant, 50 μL was cleaned by solid phase extraction (SPE) on C18+ Stage tip columns (prepared in-house).^30^ Clean-up and elution of the peptides were carried out as described before.^6^ Lastly, peptides were reconstituted in 50 μL of 1 mL/L formic acid in water.

#### LC–MS/MS

Cleaned peptide samples were analyzed using a nanoLC–MS/MS mass spectrometry system (Thermo EASY nLC1000 connected to a Thermo Orbitrap XL) in which the Orbitrap was used to measure both MS and MS/MS scans. A volume of 18 μL of sample was injected onto a 0.10 × 32 mm Magic C18AQ 200A 5 μm beads (Bruker Nederland B.V.) preconcentration column (prepared in-house) at a constant pressure of 800 bar (normally resulting in a flow of ca. 11 μL/min). Peptides were eluted from the preconcentration column onto a 0.10 × 250 mm Magic C18AQ 200A 3 μm beads analytical column (prepared in-house), and separation of the peptides took place at a flow rate of 0.5 μL/min with a gradient of acetonitrile. In 50 min, the gradient increased from 5% to 30% acetonitrile in water with 1 mL/L formic acid, followed by a 3 min cleaning of the column by a fast increase to 50% acetonitrile. Between preconcentration and analytical column, a P777 Upchurch microcross was positioned, with a stainless-steel needle fitted into the waste line. Using this needle, a 3.5 kV electrospray potential was applied to the eluent. Full scan positive mode FTMS spectra were obtained with the Orbitrap at a resolution of 15 000 and within the range of *m*/*z* 280 to 1400. For the most abundant doubly and triply charged peaks in the FTMS scans, CID (isolation width 2 *m*/*z*, 28% normalized collision energy, activation Q 0.25 and activation time 15 ms) MS/MS scans were recorded in data-dependent mode at a resolution of 7500 in the Orbitrap as well (MS/MS threshold 10 000, 45 s exclusion duration).

#### Data Analysis (Peptides)

Raw LC–MS/MS data files from peptidomics analysis were processed similar to the proteomics data, with some differences. In silico digestion was carried out with unspecific digestion settings and a peptide length set to a minimum of 8 and a maximum of 25 amino acids. Variable modifications were set to acetylation of the protein N-term, oxidation of methionine, deamidation of asparagine and glutamine, and phosphorylation of serine and threonine. A maximum of 5 variable modifications were allowed per peptide sequence. LFQ intensities were used to further analyze the data and imputed with the same algorithm as the proteomics data.

Filtering was applied on the MaxQuant output to reduce the number of false positives. Identifications that were removed matched with the decoy database, matched with contaminants, were only identified with a modification, or were identified in less than 10 out of 29 samples. Imputation and selection of a leading protein was carried out using the same approach as for the proteomics data.

The mass spectrometry peptidomics data have been deposited to the ProteomeXchange Consortium via the PRIDE^29^ partner repository with the data set identifier PXD028294.

### Metabolomics

#### Sample Preparation

Human milk samples were prepared and analyzed with NMR as described in previous studies.^31,32^ In brief, samples were thawed at 4 °C and centrifuged for 30 min at a speed of 12 000 rpm (Eppendorf centrifuge 5424, Eppendorf AG, Hamburg, Germany). Next, 500 μL of supernatant was added to 500 μL of deuterated chloroform, and this was thoroughly mixed for 30 min. This mixture was again centrifuged for 15 min at 10 000 rpm. The aqueous top layer was obtained and with an equal volume of phosphate buffer (pH = 7) transferred to a Pall 3K omega filter (10–20 kDa cutoff, OD003C34; Pall, Washington, NY, USA). The filtrate obtained by centrifugation at 10 000 rpm for 30 min was transferred to a 3 mm NMR tube.

#### NMR Analysis

NMR measurements were carried out using a Bruker Avance III NMR spectrometer with a 600 MHz/54 mm UltraShielded Plus magnet. The spectrometer was equipped with a CryoPlatform cryogenic system for cooling, a BCU-05 cooling unit, and with an ATM automatic tuning and matching unit (Bruker Biospin, Rheinstettten, Germany). Samples were measured in ^1^H NMR tubes of 3 mm (Bruker matching system). One-dimensional nuclear Overhauser effect spectroscopy (NOESY) spectra were obtained at a temperature of 300 K. All obtained spectra were corrected with automatic baseline correction and aligned to the resonance of alanine (1.484 ppm). The Human Metabolome Database version 4 (http://hmdb.ca) and published literature were used for the assignment of metabolites to the spectra.^33^ Full details on parameters used for NMR analysis can be found in the Supporting Information listing S1.

#### Data Analysis (Metabolomics)

NMR data were aligned, and the water region was removed. To minimize overlap in the spectra, NMR resonances were specifically integrated by careful selection of peaks. A selection of one NMR resonance was made in case a metabolite was represented by multiple resonances in the NMR spectra. Nonoverlapping peaks were chosen for further data analysis. In case baseline correction resulted in negative intensities, a value of 0.0001 was imposed to replace these. All NMR resonances were scaled to unit variance before correlations were investigated.

### Statistical Analysis

All statistical analyses were performed using R version 4.0.1.^27^ Interindividual variation was calculated as coefficient of variation (CV), which is also known as relative standard deviation and expressed as percentage.

#### Weighted Correlation Network Analysis (WGCNA)

To reduce dimensionality and to elucidate patterns of cross-correlation present in the proteomics and peptidomics data, a weighted correlation network analysis was carried out using the WGCNA package for R (version 1.70.3).^34^

With this analysis, a set of clusters was obtained for each data set, where each cluster consists of highly correlating proteins or peptides. Details of WGCNA applied to proteomics data were described by Pei et al.^20^ In brief, a correlation matrix was obtained using the biweight midcorrelation measure. From this, a signed and weighted network was created, to which a soft-thresholding power was applied. This soft-thresholding power was chosen based on the approximation of scale-free topology. As shown in the Supporting Information, a power of 5 was chosen for the proteomics data (see Supplemental Figure S2) and a power of 8 for the peptidomics data (Supplemental Figure S4). By applying this power, noise was removed, and the strength of correlations was enhanced. After this, topology overlap metrics were calculated from the network and were subjected to hierarchical clustering. Clusters were obtained from the dendrogram using the cutreeDynamic function with a minimum cluster size of 15. The eigenvalues of the clusters (later referred to as eigenproteins and eigenpeptides) were used to investigate relations between the data sets.

Relationships between characteristics of the samples or mothers, eigenproteins, eigenpeptides, and metabolites were assessed using Spearman’s rank correlation (denoted with ρ). To calculate statistical significance, the “corPvaluestudent” function from the WGCNA package was used, which provides Student asymptotic *p*-values.

#### Gene Overrepresentation

To investigate whether protein clusters resulting from the WGCNA were characterized by specific gene ontology (GO) annotations, a GO overrepresentation analysis was carried out using the R package ClusterProfiler, version 3.16.1.^35^ The “enrichGO” function was used in combination with the “compareCluster” function with as background all identified proteins. GO terms were obtained from the org.Hs.eg.db package.^36^ On the output of the overrepresentation analysis, the “simplify” function was applied to remove redundant GO annotations. For this, the “Wang” measure, and a similarity cutoff of 0.7, was used. Overrepresentation was visualized using dot plots in which the GO annotations with the top 3 most significant GO terms were shown.

#### Sequence Logos

Sequence logos were created for the P1 and P1′ positions of the peptides’ N- and C-terminal ends. This was done based on both frequency and intensity of the amino acid in the P1 and P1′ position using the R package ggseqlogo, version 0.1.^37^

## Results and Discussion

**Figure 1:**
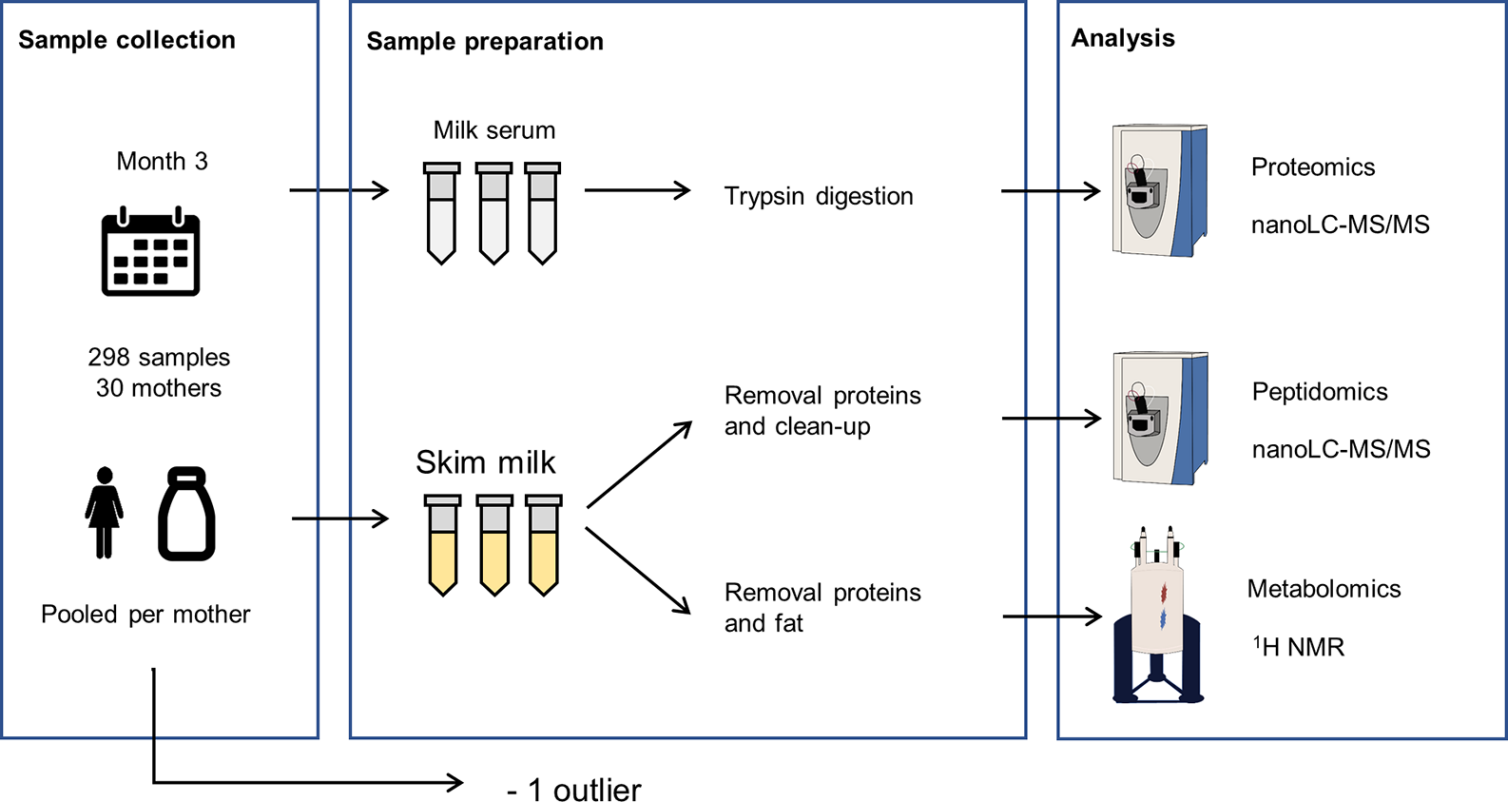
Schematic overview of the workflow used for the analysis of the human milk proteome, peptidome, and metabolome.

In this study, the human milk peptidome, proteome, and metabolome of 29 mothers were analyzed (see Figure 1). With the resulting data, the interindividual variation in mature human milk was investigated.

### Proteomics

After analysis and subsequent filtering, 237 proteins were identified and quantified with label-free quantification (LFQ). The number of identified proteins per sample ranged from 110 to 228. From these 237 proteins, 84% were identified in more than half of the 29 samples. An overview of all identified proteins can be found in Supplemental Table S1.

**Figure 2:**
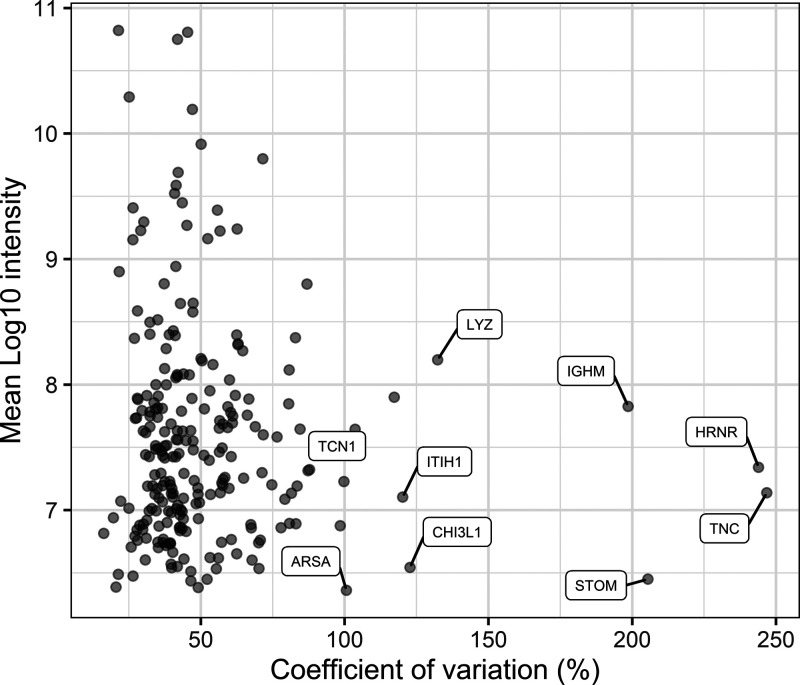
Distribution of the variation of proteins. The overall CV (%) on the *x*-axis versus the mean log10 of the LFQ intensities for each identified protein on the *y*-axis. Proteins with the largest variation are labeled with their respective gene code.

As shown in Figure 2, only 4.2% (*n* = 10) of the identified proteins show an extensive overall CV of >100%. From this figure, it can also be noted that high abundant proteins show a relatively low variation between samples when compared with low abundant proteins. This corresponds with previous studies in which a relatively low variation was found for the most abundant human milk proteins.^14,38^ In addition, it was observed that the overall variation in proteins (median CV = 42.8%) surpasses to a great extent the technical variation (median CV = 20.4%) (see Supplemental Figure S1A). Furthermore, it was found by Zhang et al. that the interindividual variation also surpasses the intraindividual variation in the proteome of human milk.^12^ This indicates that the major contributor to the overall variation is interindividual variation. This is a pattern also found for the proteome of other body fluids.^39−41^

A few proteins show a remarkably large interindividual variation (Table 2). From these, the first four will be discussed more in detail. Tenascin (TNC) is well-known for its neutralizing effect on HIV.^42^ Whereas the decrease of TNC over time was shown to become stable after 30 days postpartum,^43^ it was found that the concentration of TNC in milk from HIV negative mothers (21 to 46 days postpartum) can range from around 0.1 to more than 100 μg/mL.^44^ This is in line with the large variation observed in the current study, where all donating mothers tested HIV negative. Although little is known about the expression of TNC in milk, it is known that TNC synthesis is rapidly induced in many tissues in response to pathological stress and inflammation.^45^ Mills et al. showed, in line with this, that airway epithelial cells generate TNC in response to viral infection.^46^ Furthermore, Sur et al. showed recently that exosomes in plasma from COVID-19 patients contain significantly increased levels of TNC, triggering pro-inflammatory cytokine signaling.^47^ Hence, it can be hypothesized that a higher level of TNC in milk might be an attempt to protect the offspring against the transmission of viral infections from the mother. Alternatively, high TNC levels could indicate an inflammatory response in the mother (e.g., mammary gland), although the donors were reported healthy at the time of the donations. For the second protein, hornerin (HRNR), it is known that it is expressed in regenerating and psoriatic skin.^48^ Nevertheless, none of the donating mothers mentioned psoriasis as an underlying disease in the current study. In addition, HRNR has also been found to be differently expressed in breast epithelial cells that are in different stages of mammary development.^49^ A significant difference was observed in HRNR staining of murine mammary tissue during lactation and at the onset of involution.^49^ This might be due to epithelial cell turnover or apoptosis and could explain the large interindividual variation for this protein. The third protein, stomatin (STOM), is a protein found in the plasma membrane associated with lipid rafts. The presence of STOM in milk is dependent on energy balance in the lactation of cows.^31^ Nevertheless, little is known about the function or expression of this protein in human milk, and a cause for its large interindividual variation remains speculative. The fourth protein, immunoglobulin M (IgM), is secreted into milk as sIgM and is secreted in the same way as secretory immunoglobulin A (sIgA). Whereas most other identified immunoglobulins show a CV < 100%, cDNA FLJ41552 fis (UniProt ID: Q6ZW64) is highly similar to the constant region of IgA and has a CV > 100% as well (Table 2). Using ELISA, two studies showed a large interindividual variation of IgM in the first 2 weeks of lactation.^50,51^ Although there is a gradual decrease of this protein over lactation,^12,52^ it was found that its interindividual variation in mature milk is larger than the other immunoglobulins.^53^

**Table 2:** Top 10 Proteins with the Largest Interindividual Variation (CV)

protein ID	protein name	gene	mean log10 intensity	CV (%)
P24821	Tenascin	TNC	7.1	246.8
Q86YZ3	Hornerin	HRNR	7.3	243.9
P27105	Stomatin	STOM	6.5	205.5
P01871	Immunoglobulin heavy constant mu	IGHM	7.8	198.6
P61626	Lysozyme C	LYZ	8.2	132.3
P36222	Chitinase-3-like protein 1	CHI3L1	6.5	122.8
P19827	Interalpha-trypsin inhibitor heavy chain H1	ITIH1	7.1	120.2
Q6ZW64	cDNA FLJ41552 fis	NA	7.9	117.2
P20061	Transcobalamin-1	TCN1	7.6	103.7
P15289	Arylsulfatase A	ARSA	6.4	100.6

It should be noted that in the current study, pooled samples were used from the third month postpartum. It is known that, in this month, longitudinal variation due to the maturation of the milk has leveled out.^22,23^ The influence of intraindividual variation is therefore expected to be minor. Nevertheless, in case of large intraindividual variation due to single outliers before pooling, the effect on interindividual variation is reduced due to the pooling of the samples.^54^

To examine whether proteins with high interindividual variation relate to specific biological processes or sample characteristics, a coexpression network was constructed using weighted correlation network analysis (WGCNA). With this, a set of 5 protein clusters was identified (see Supplemental Figures S2 and S3).

**Figure 3:**
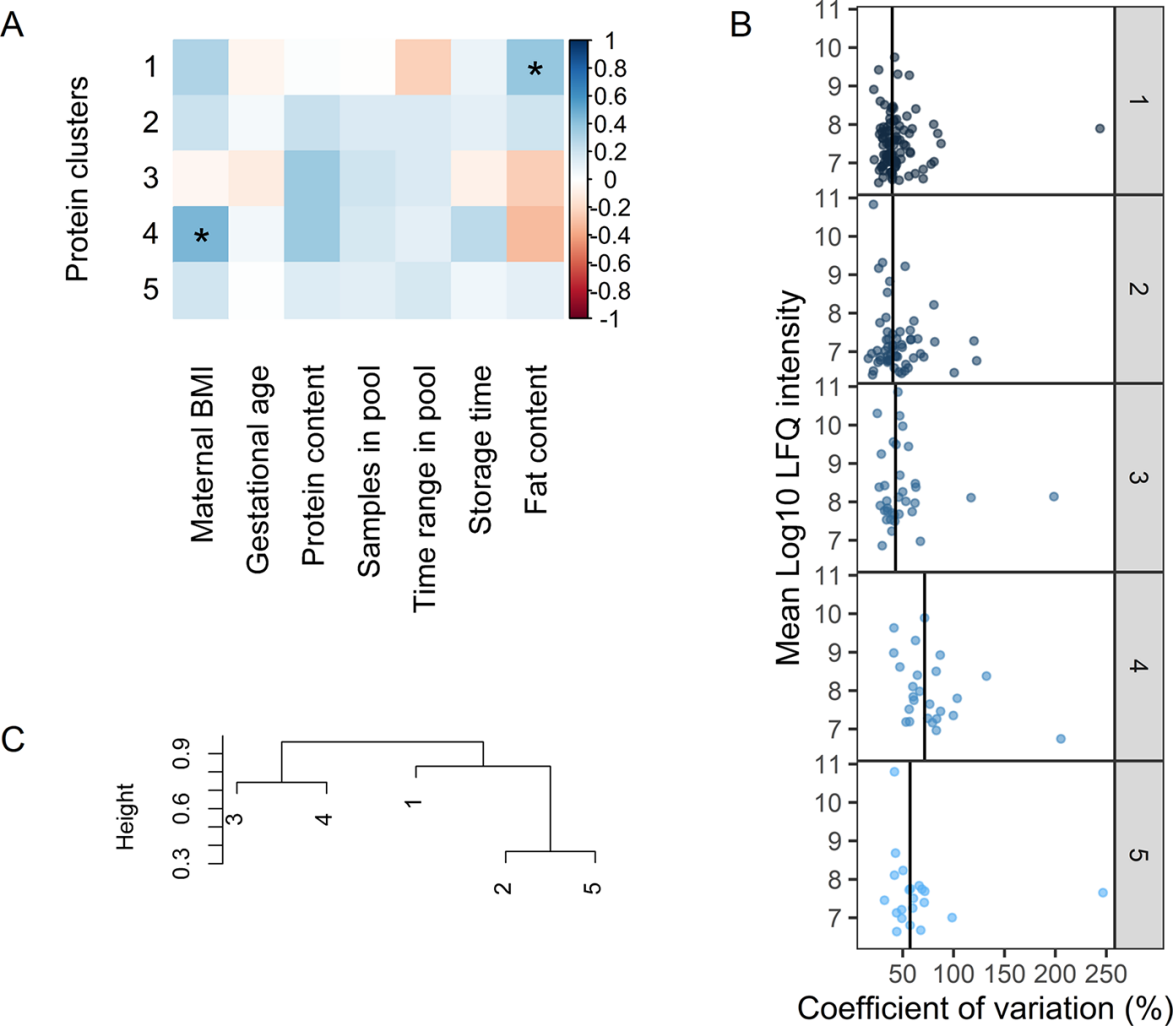
(A) Association of eigenproteins with subject and sample characteristics using spearman correlation. Significant correlations are annotated with ∗ (*p* < 0.05). (B) Interindividual variation (CV) in proteins per WGCNA cluster. Vertical lines indicate the median CV of the cluster. (C) Hierarchical clustering of the eigenproteins of each cluster.

**Figure 4:**
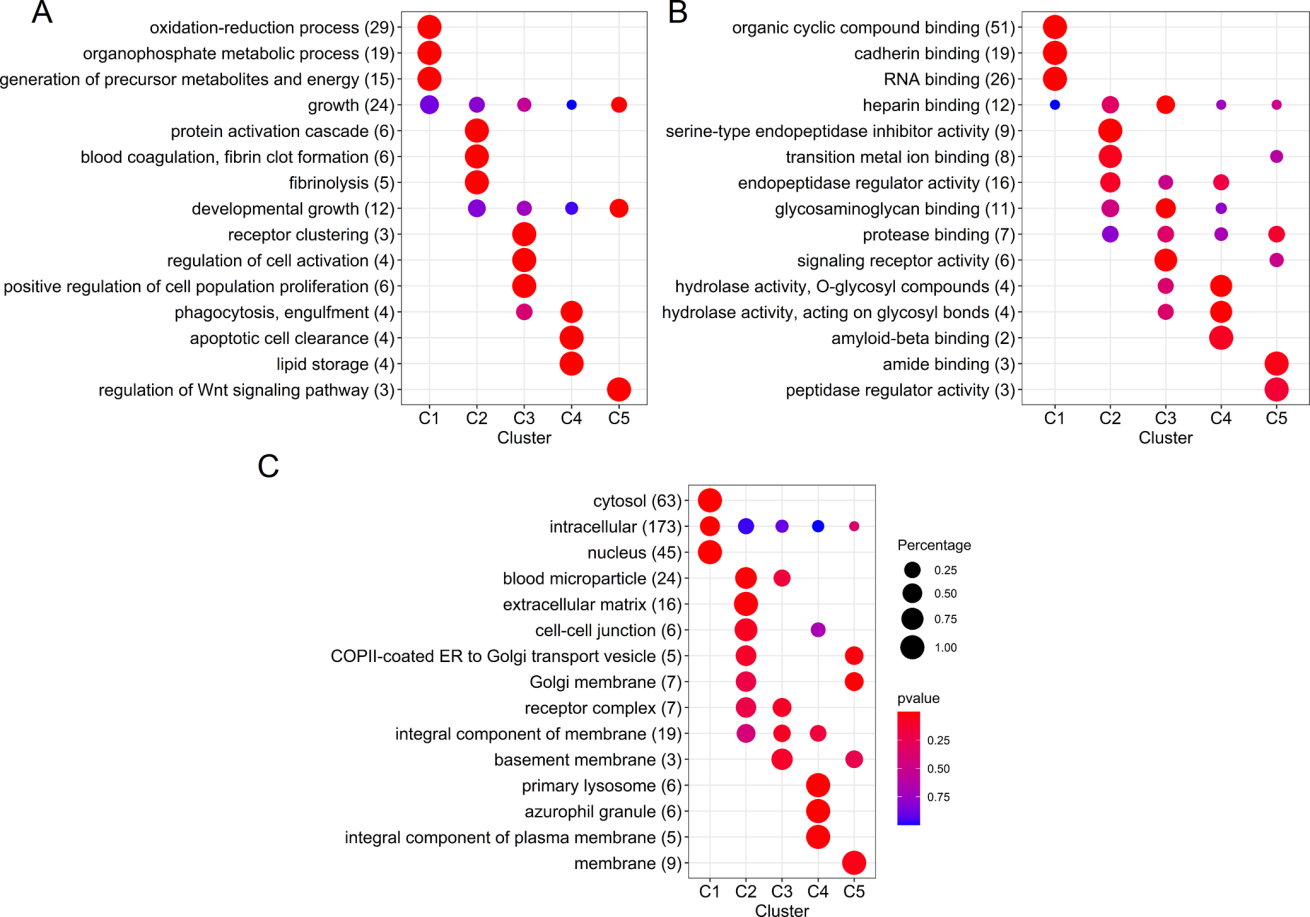
Overrepresented GO annotations in each WGCNA cluster of the proteomics data, with (A) biological processes, (B) molecular functions, and (C) cellular components.

As can be seen in Figure 3B, the largest interindividual variation is present in cluster 4 with a median CV of 71.6% and containing medium abundant proteins. This cluster includes nonmicellar caseins and milk fat globule membrane (MFGM) related proteins such as butyrophilin, lactadherin, lipoprotein lipase, lysozyme C, platelet glycoprotein 4, stomatin, and mucins. This suggests the coabundance of proteins involved in the pathway of MFGM secretion by the mammary epithelial cell. When comparing the gene annotations of the clusters (Figure 4), cluster 4 contains specifically proteins annotated with lipid storage and phagocytosis, biological processes typical for MFGM proteins.^55^ As can be seen in Figure 3A, a positive relation was found between protein cluster 4 and maternal BMI (ρ = 0.45, *p* = 0.01). The strongest correlation between individual proteins in this cluster and BMI was found with the antiadhesive protein podocalyxin (PODXL). A study by Crujeiras et al. showed that PODXL is negatively associated with methylation levels in subcutaneous adipose tissue and suggested that there may be an epigenetic regulation associated with obesity.^56^ Nevertheless, further research is needed to investigate the relation between PODXL in human milk and maternal BMI.

From the other clusters, clusters 1 and 2 show a similar pattern with low abundant proteins and relatively low variation (median CVs of 39.7 and 40.4%, respectively). Cluster 1 comprises the majority of the proteins, with annotations showing involvement in energy pathways and metabolism (Figure 4A), and has a positive association with total fat content (ρ = 0.39, *p* = 0.04) (see Figure 3A). It has been suggested that there might be a common regulation for lipid and protein synthesis in milk.^57^ Although the measurements of total protein and fat content in this study do not show a correlation, the observed association might be due to a selective pathway, concerning a selection of the proteins present in human milk.

Cluster 2 is characterized by proteins with serine-type endopeptidase inhibitor activity (Figure 4B), among which are α-1-antitrypsin (SERPINA1), plasma protease C1 inhibitor (SERPING1), and α-2-macroglobulin (A2M), proteins also involved in blood coagulation. In addition, this cluster contains a majority of other blood originating proteins such as albumin (ALB) and haptoglobin (HP) (Figure 4C). This indicates a coabundance, and possibly related/shared pathways, of these proteins and the protease systems present in milk.

Cluster 3 comprises many of the immune proteins of milk such as polymeric immunoglobulin receptor (PIGR), immunoglobulin A (IgA), immunoglobulin M (IgM), J chain, and lactoferrin (LF). This cluster comprises, in general, medium and high abundant proteins with low variation (median CV = 43%). Several lines of research have suggested a relation between immune proteins in milk and their degradation by proteases.^14,58^ However, such correlation was not observed in the current study, possibly because the focus was on interindividual variation, and Elwakiel et al.^14^ studied longitudinal (intraindividual) variation, observing large variation for these proteins over lactation.

Cluster 5 has a median CV of 43% and, in general, low abundant proteins. This cluster is closely related to cluster 2 (see Figure 3C) and does not seem to be characterized by large protein groups with unique biological processes or molecular functions (Figure 4).

Overall, these results indicate that there is a high interindividual variation in several specific proteins as well as in a cluster of coabundant proteins containing MFGM related proteins and nonmicellar caseins.

### Peptidomics

For the analysis of the peptides, proteins were removed from the milk by precipitation. LC–MS/MS analysis of the supernatant and subsequent filtering of the data resulted in the identification of 740 peptides originating from 23 different precursor proteins. The number of peptides identified per sample ranged from 440 to 637. A major part of the identified peptides (38.4%) originated from β-casein, followed by polymeric immunoglobulin receptor (PIGR) (16.5%) and osteopontin (8.5%). This overrepresentation of peptides from a few proteins is probably due to the high abundance of these proteins in combination with direct or indirect association with plasminogen and sensitivity for proteolysis.^59^ This pattern is typical for human milk peptidomics and corresponds to previous findings.^6,60^ An overview of all identified peptides can be found in Supplemental Table S2.

**Figure 5:**
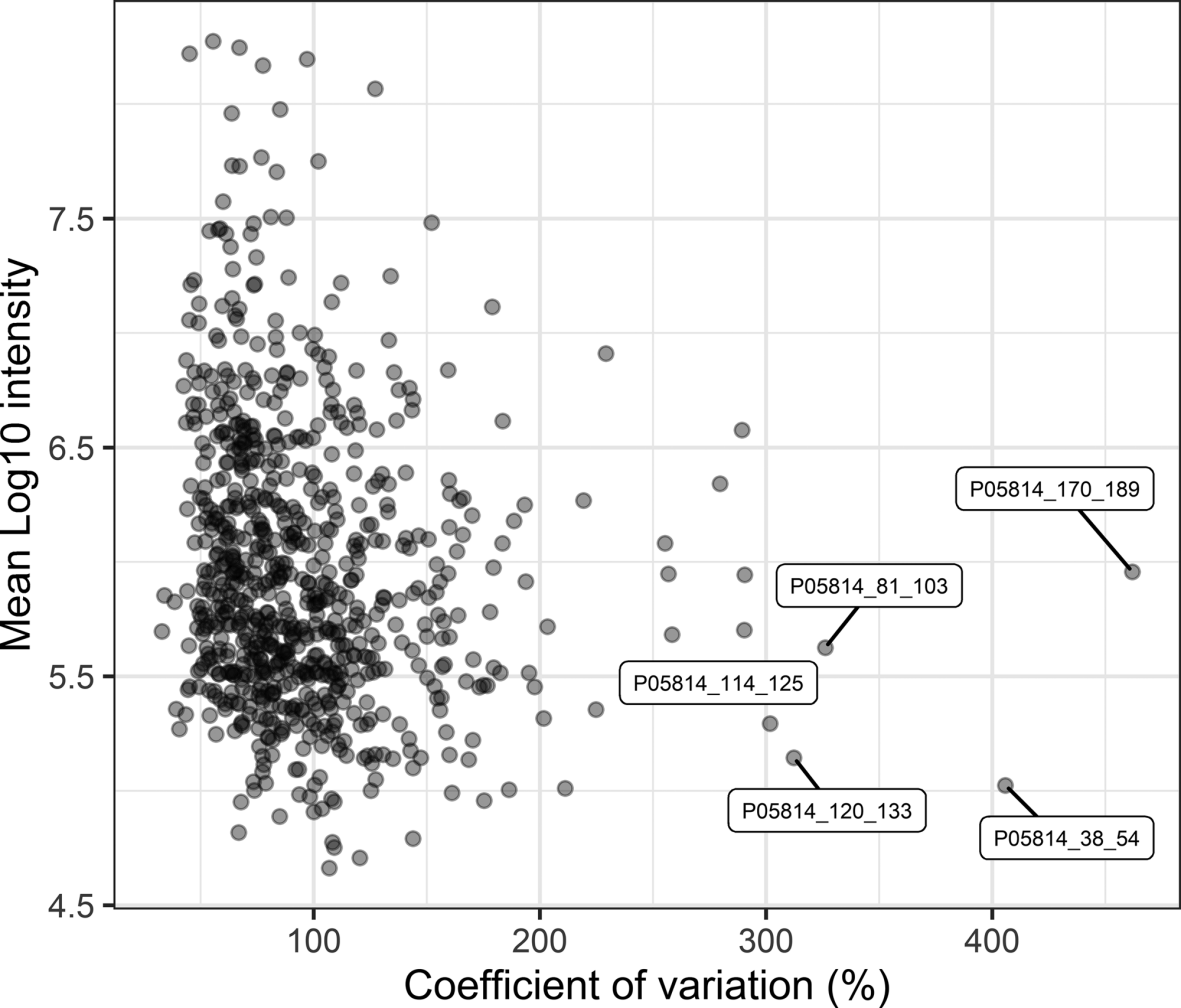
Distribution of the variation of peptides. The overall CV (%) on the *x*-axis versus the mean log10 of the LFQ intensities for each identified peptide on the *y*-axis. Peptides with the largest variation are labeled with the UniProt ID of their respective precursor protein and their range in the protein sequence.

As shown in Figure 5, 36.2% (*n* = 268) of the identified peptides show an overall CV of >100%. This figure shows that, like the proteomics data, high abundant peptides show a relatively low variation compared with the lower abundant peptides. In addition, the overall variation (median CV = 85.2%) is substantially larger than the technical variation (median CV = 21.8%) (see Supplemental Figure S1B). This indicates that, also for the peptidome, the major contributor to the overall variation is interindividual variation.

It can be noted that the overall variation in peptides is larger than the variation in proteins. This difference is not observed in the technical replicates, where the median and maximum CV for proteins are 20.4 and 107% and for peptides 21.8 and 101%. Therefore, it can be concluded that interindividual variation in the human milk peptidome is substantially larger than in the proteome.

In Table 3, the 10 peptides with the highest interindividual variation are shown. All but one of these peptides are from the precursor protein β-casein. Proteolysis of human milk proteins depends on a complex system of proteases, protease inhibitors, and other factors. Therefore, it can be hypothesized that highly variable peptides are, for example, to a variable extent, further degraded depending on the balances in the proteolytic systems. Most of these peptides are relatively long and originate from a region in the protein sequence that is heavily hydrolyzed. Many possible precursor and product peptides of these peptides were also identified, indicating that further proteolysis of these peptides is highly variable and results in their large interindividual variation.

**Table 3:** Top 10 Peptides with the Largest Interindividual Variation (CV)

sequence	protein ID	peptide range	mean log10 intensity	CV (%)
SVPQPKVLPIPQQVVPYPQR	P05814	170–189	6.0	461.9
KVKHEDQQQGEDEHQDK	P05814	38–54	5.0	405.8
ILPLAQPAVVLPVPQPEIMEVPK	P05814	81–103	5.6	326.2
SPTIPFFDPQIPKL	P05814	120–133	5.1	312.3
VMPVLKSPTIPF	P05814	114–125	5.3	301.8
SVPQPKVLPIPQQVVPYPQ	P05814	170–188	5.9	290.6
SDISNPTAHENYEKNNVMLQW	P47710	165–185	5.7	290.3
GRVMPVLKSPTIPF	P05814	112–125	6.6	289.3
LAPVHNPISV	P05814	217–226	6.3	279.6
DTVYTKGRVMPVLKSPTIPF	P05814	106–125	5.7	258.4

Little is known about longitudinal variation in the human milk peptide profile. However, it is expected that there is less variation in the third month postpartum due to the maturation of the milk. This is in line with the observation that the activity of plasmin, the main human milk protease, decreases over time.^61^ Nevertheless, future research is needed to confirm this. In addition, the effect of single outliers before pooling, that is, large intraindividual variation, will be less reflected in the interindividual variation due to the pooling of the samples.

**Figure 6:**
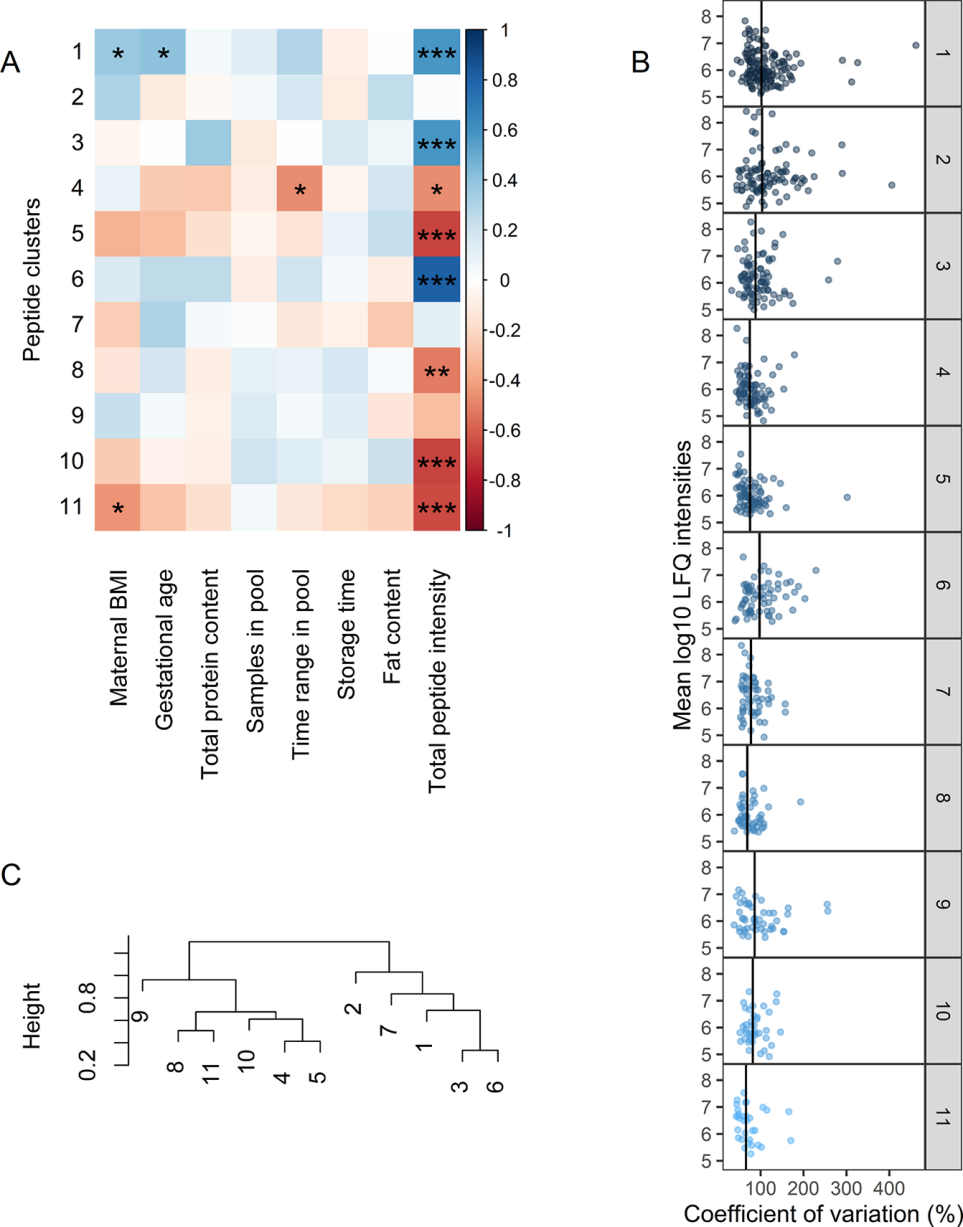
(A) Association of peptide clusters with subject and sample characteristics using Spearman correlation. Significant correlations are annotated with ∗ (*p* < 0.05), ∗∗ (*p* < 0.01), or ∗∗∗ (*p* < 0.001). (B) Interindividual variation (%) in peptides per WGCNA cluster. Vertical lines indicate the median CV of the cluster. (C) Hierarchical clustering of the eigenpeptides of each cluster.

Similar to the proteomics data, WGCNA was applied to the peptidomics data, resulting in 11 clusters of coabundant peptides (see Supplemental Figures S4 and S5). Although to our knowledge, this is the first time WGCNA has been applied to peptidomics data, clustering of coabundant peptides might provide insights into the different factors that affect the proteolytic degradation of proteins in milk. As can be noted from Table 4, several of the clusters are distinctly dominated by peptides from certain precursor proteins. Furthermore, there are several significant correlations between eigenpeptides and sample characteristics (Figure 6).

**Table 4:** Peptide Clusters with Their Size and Dominating Precursor Proteins

cluster label	cluster size	top precursor protein ID	top precursor protein name	number peptides per precursor protein
1	132	P05814	Beta-casein	98
		P47710	Alpha-S1-casein	7
2	91	P05814	Beta-casein	39
		P10451	Osteopontin	15
3	83	P05814	Beta-casein	39
		P47710	Alpha-S1-casein	13
4	77	P01833	Polymeric immunoglobulin receptor	34
		P05814	Beta-casein	23
5	74	Q99541	Perilipin-2	22
		P15941	Mucin-1	19
6	62	P05814	Beta-casein	40
		P10451	Osteopontin	6
		P47710	Alpha-S1-casein	6
7	53	P05814	Beta-casein	33
		P47710	Alpha-S1-casein	10
8	49	P01833	Polymeric immunoglobulin receptor	20
		P10451	Osteopontin	14
9	47	P01833	Polymeric immunoglobulin receptor	26
		P07498	Kappa-casein	7
10	40	Q13410	Butyrophilin subfamily 1 member A1	18
		Q99541	Perilipin-2	8
11	32	P01833	Polymeric immunoglobulin receptor	13
		Q13410	Butyrophilin subfamily 1 member A1	7

The largest interindividual variation can be observed in clusters 1, 2, 3, and 6 (median CV of 102.4, 103.7, 88.4 and 97.6%, respectively), which are dominated by peptides from β-casein, α_S1_-casein, and osteopontin (Table 4).

**Figure 7:**
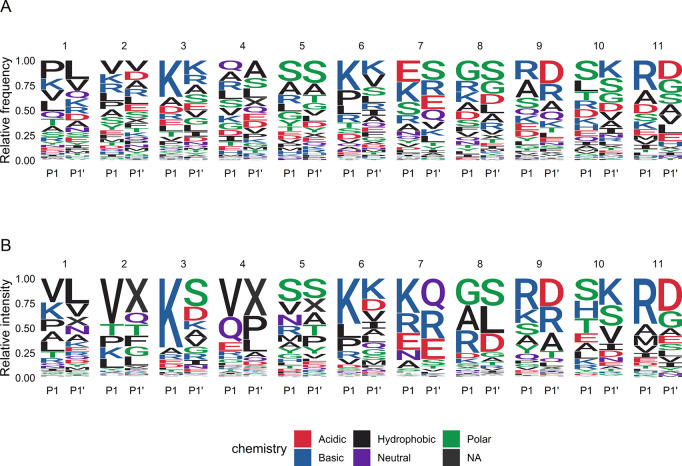
(A) Relative frequencies, and (B) relative intensities of amino acids in P1 and P1′ position for each peptide cluster. When P1 or P1′ position is the C-terminal or N-terminal end of the protein sequence, the empty position is annotated with “X”.

Several peptides with large variation (Table 3) show coabundance in the first three clusters. For example, two of these peptides (SVPQPKVLPIPQQVVPYPQR and SVPQPKVLPIPQQVVPYPQ) occur in cluster 1 and are only different in one amino acid. This coabundance of peptides shows that the level of a certain peptide can depend on the level of a larger, precursor peptide. When it comes to the responsible proteases, this could mean that further digestion of precursor peptides by, for example, nonspecific carboxypeptidases is dependent on cleavage of the proteins by a more specific protease such as plasmin. It is known that plasmin cleaves preferentially with lysine (K) or arginine (R) in the P1 position. From Figure 7A, it can be seen that from the clusters dominated by casein peptides, especially clusters 3 and 6 are characterized by many peptides with K or R in the P1 position, matching plasmin specificity. Besides clusters 1, 2, 3, and 6, cluster 7 is also dominated by peptides from β-casein and α_S1_-casein. Nevertheless, this cluster has a much lower median variation (77.7%), and the β-casein peptides in this cluster are exclusively from the N-terminal end of the protein (sequence position 16 to 54, see Supplemental Table S2). This suggests that the N-terminal of β-casein is proteolyzed with different driving factors and lower interindividual variation than the rest of the sequence. Since cleavage specificity of this cluster is not unique (Figure 7), factors such as structure, peptidase activity, or protease inhibition might cause the difference with the other clusters. Nevertheless, proteolysis resulting in the peptides in cluster 7 does not seem to be influenced by total proteolytic activity (Figure 6A). This was also observed for cluster 2, even though both clusters comprise several highly abundant peptides (Figure 6B). This suggests that higher proteolytic activity seems specific for certain proteins and even protein regions. Peptide clusters dominated by β-casein and α_S1_-casein are associated more with each other than with the clusters dominated by other precursor proteins (Figure 6C). This indicates that the degradation of caseins is distinct from the degradation of other proteins, which might be due to their association in micelles.

Clusters 4, 8, 9, and 11 are dominated by peptides from PIGR, with a median CV of 74.9, 69.2, 86.1, and 66.1%, respectively. Clusters 5 and 10 are dominated by peptides from MFGM proteins (median CV of 75.5 and 81.8%, respectively). The clustering of peptides of MFGM proteins is in line with Giuffrida et al., who proposed a specific mechanism for proteolysis of MFGM proteins by proteolytic enzymes in the alveolar cell membranes.^62^ This is further supported by the fact that most peptides in these clusters do not match the specificity of plasmin (Figure 7). As shown in Figure 6, several clusters dominated by peptides from PIGR or MFGM related proteins (5, 8, 10, and 11) show a strong negative correlation with total peptide intensity. As was observed before, higher proteolytic activity seems to attribute mainly to an increase in the intensity of peptides originating from β-casein and α_S1_-casein, possibly driven by plasmin activity and leading to the largest interindividual variation.

Taken together, these results show that the largest interindividual variation is present in peptides of β-casein, α_S1_-casein, and osteopontin. With WGCNA, 11 distinct clusters of peptides were obtained, showing differences in characteristics related to proteolytic degradation, such as precursor proteins, cleavage patterns, and association with total peptide intensity.

### Metabolomics

Metabolomics analysis with NMR resulted in the identification of 40 metabolites, among which were fatty acids, free amino acids, oligosaccharides, and other small molecules. A detailed list of all identified metabolites can be found in Supplemental Table S3.

**Figure 8:**
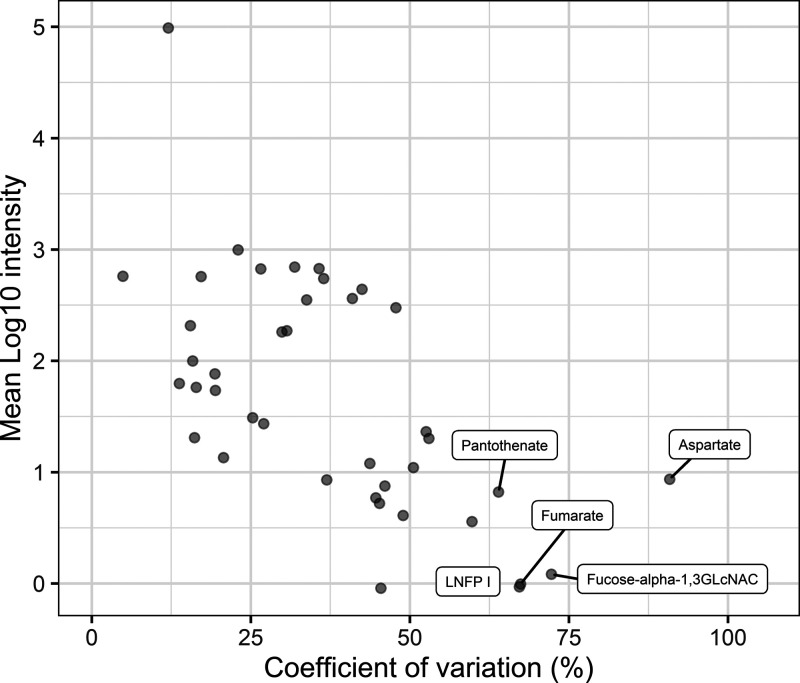
Distribution of the variation of metabolites. The overall CV (%) on the *x*-axis versus the mean log10 of the intensities for each identified metabolite on the *y*-axis. Metabolites with the largest variation are labeled.

As shown in Figure 8, similar to the proteome and peptidome, metabolites with high intensity also show low interindividual variation. Overall variation between the samples is for the majority of the metabolites larger than the technical variation (see Supplemental Figure S1C).

All metabolites identified show a CV < 100%, a low variation compared to the proteomics and peptidomics data. The variation in metabolites is similar to the results reported by Smilowitz et al., with the notable exception of butyrate and formate.^63^ Smilowitz et al. found a CV of 77.3 and 121% for these metabolites, whereas in the current study, these metabolites had a CV of 4.9 and 36.9%, respectively. One explanation for this difference might be the sample collection. Whereas in the current study, samples were pooled, Smilowitz et al. used nonpooled samples. Larger variation in single samples versus smaller variation in pooled samples might be due to high intraindividual variation, that is, variation between different feedings.

It is proposed from several studies that a large part of the interindividual variation in the human milk oligosaccharide (HMO) metabolome is due to secretor status and Lewis blood type.^63−66^ On the basis of intensities of 2′-fucosyllactose (2’FL) and lacto-n-fucopentaose I (LNFP I), 3 out of the 29 donating mothers in the current study are proposed as nonsecretors (Se−) (see Supplemental Figure S6). On the basis of intensities of 3′FL, LNFP III, and lactodifucotetraose (LDFT), 2 out of the 29 mothers are proposed to be Lewis negative (Le−), of which one was also Se– (see Supplemental Figure S6). Removal of the Se– and Le– samples (*n* = 4) from the calculations shows decreases in the interindividual variation of the HMO metabolites (see Table 5 and Supplemental Table S3). Nevertheless, it should be noted that there remains a substantial interindividual variation in, for example, LNFP I, 3′-FL, and fucose-α-1,3-GLcNAC. This is an important finding considering the important role of HMOs in the healthy development of the infant.

**Table 5:** Metabolites with Highest (top 10 rows) and Lowest (bottom 10 rows) Interindividual Variation (CV) Together with Interindividual Variation in Samples from Mothers Proposed to Be Secretors (Se(+)) as Well as Lewis Positive (Le(+))

metabolite	CV (%)	CV (%) Se(+)Le(+)
Aspartate	90.8	89.3
Fucose-alpha-1,3GLcNAC	72.2	58.1
Fumarate	67.4	64.7
LNFP I	67.2	63.0
Pantothenate	63.9	64.1
CMP	59.8	52.2
3′-FL	53.0	41.1
LDFT	52.6	44.6
2′-FL	50.6	37.2
Histidine	48.9	50.6
Methionine	19.4	18.7
Lacto-N-difucohexaose II	19.4	13.8
Urea	17.2	17.0
Lactate	16.4	16.0
Valine	16.2	17.1
Acetate	15.9	17.0
Alanine	15.5	16.1
cis-Aconitate	13.8	11.2
Lactose	12.0	9.5
Butyrate	4.9	4.8

**Figure 9:**
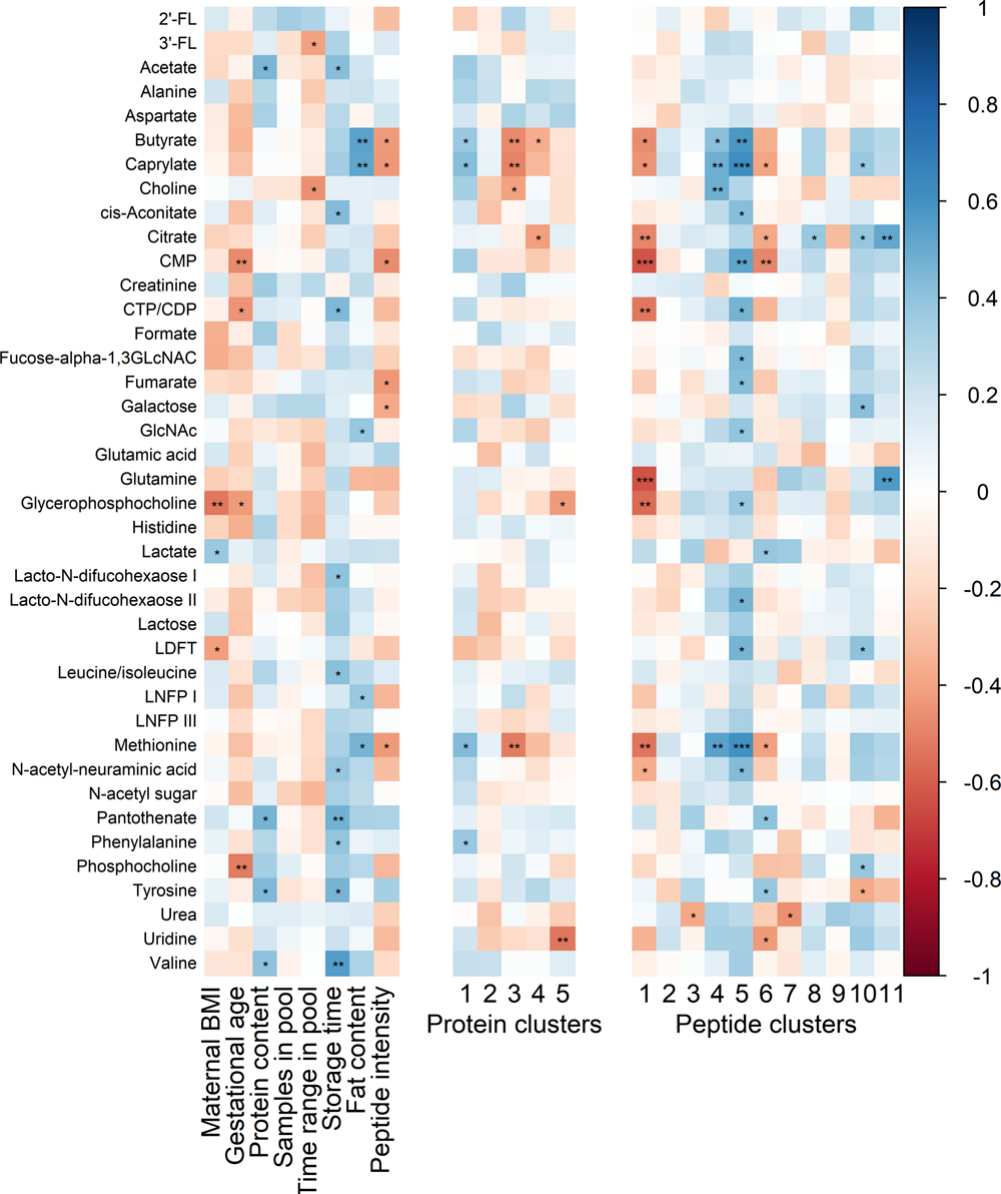
Associations of metabolites with subject and sample characteristics (left) eigenproteins (middle) and eigenpeptides (right). Significant correlations are annotated with ∗ (*p* < 0.05), ∗∗ (*p* < 0.01), or ∗∗∗ (*p* < 0.001).

Several metabolites show associations with subject and sample characteristics (Figure 9). First, a strong negative relation between glycerophosphocholine (GPC) and BMI (ρ = −0.52, *p* = 0.003) is present. It was found that in serum of patients with metabolic abnormal obesity, GPC is significantly decreased.^67^ Future research is necessary to show whether this holds for human milk as well.

Second, cytidine monophosphate (CMP), cytidine triphosphate and diphosphate (CTP/CDP), GPC, and phosphocholine (PC) all show a significant negative correlation with maternal age (ρ < −0.42, *p* < 0.022). These metabolites play an important role in the synthesis of the cellular membranes. Therefore, their negative correlation with maternal age might be a marker of the known decrease in mammary epithelial cell proliferation at a higher age.^68,69^ This also accords with Wei et al., who found that PC in bovine milk is negatively correlated with energy balance and proposed a relation with cell proliferation.^70^

Third, the fatty acids butyrate (C4:0) and caprylate (C8:0), and the amino acid methionine show a positive relation with fat content (ρ > 0.53, *p* < 0.003) and a negative relation with total peptide intensity (ρ < −0.44, *p* < 0.018). This relation with methionine could point to the involvement of this amino acid in fat synthesis. Qi et al. found that methionine promotes fat synthesis through the SNAT2-PI3K signaling pathway in bovine mammary epithelial cells.^71^ If this pathway is also present in humans, it would explain the correlations observed in the current study.

Fourthly, a negative relation was found between total peptide intensity and butyrate, caprylate, CMP, fumarate, galactose, LNFP I, and methionine (ρ > −0.43, *p* < 0.02). A higher intensity of these metabolites might indicate a lower proteolytic activity in the milk. Although little is known about milk metabolites and their relation with proteolytic activity, it is known that butyrate can stimulate the secretion of plasminogen activator inhibitor 1 in colonic epithelium.^72^ Knowing that plasminogen activation needs to precede plasmin activity, this might also hold for the mammary epithelium, causing a decrease in proteolytic activity in the secreted milk.

As can be noted from Figure 9, several metabolites show a positive association with the storage time of the samples. From previous research, it is known that butyrate and acetate levels can be affected by storage time.^73^ However, the strongest associations were found with pantothenate (vitamin B5) (ρ = 0.48, *p* = 0.009) and valine (ρ = 0.55, *p* = 0.002). An increase of pantothenate during frozen storage contradicts the findings of Goldsmith et al., who reported a decrease.^74^ On the other hand, the association with valine could point to continued proteolysis during storage, resulting in more FAA. Nevertheless, no strong positive associations were found with peptide or protein clusters (Figures 6A and 3A, respectively). Therefore, further research on the influence of storage time on the metabolome of human milk is needed for more insight into this.

### Relation among Omics Data Sets

**Figure 10:**
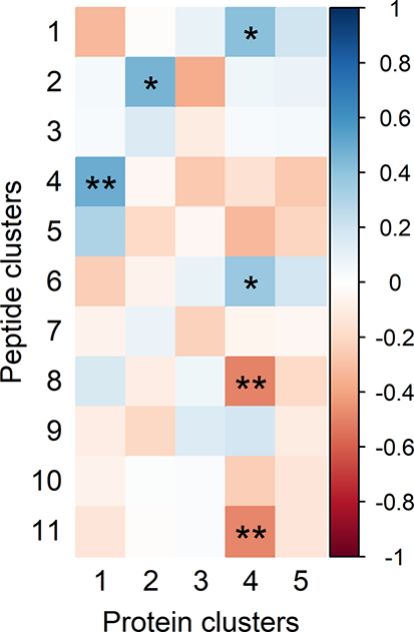
Association of eigenproteins with eigenpeptides. Significant correlations are annotated with ∗ (*p* < 0.05) or ∗∗ (*p* < 0.01).

To identify associations between the proteomics and peptidomics data, eigenproteins were compared with eigenpeptides (Figure 10). From this, several associations were found, of which the most notable will be discussed.

First, it can be observed that protein cluster 4, comprising caseins and MFGM proteins, relates positively with the β-casein-dominated peptide clusters 1 and 6 (ρ > 0.38, *p* < 0.04). From this, it can be hypothesized that the interindividual variation of part of the β-casein peptides is related to the amount of nonmicellar β-casein present in the milk. Second, protein cluster 2, which contains most serine protease inhibitors (SERPINs), relates positively with peptide cluster 2 (ρ = 0.47, *p* = 0.01). This peptide cluster is dominated by β-casein peptides but does not relate to total peptide intensity (Table 4 and Figure 6A). This points to SERPIN inhibition of serine proteases, such as thrombin and plasmin, responsible for further degradation of these peptides.

In the association of eigenproteins and eigenpeptides with metabolites (Figure 9), it was found that butyrate, caprylate, and methionine are negatively associated with protein cluster 3, which contains the majority of the immune-related proteins. In addition, these metabolites associate positively with peptide cluster 5, which is dominated by peptides from MFGM related proteins (perilipin-2 and mucin-1) and negatively with peptide cluster 1. Qi et al. reported that methionine was not only found to promote fat synthesis but also to promote protein synthesis and cell proliferation through the same SNAT2-PI3K signaling pathway.^71^ The proteolysis of MFGM related proteins by specific enzymes in the alveolar cell membranes, as discussed before, might therefore be related to cell proliferation. In addition, several other metabolites relate positively with peptide cluster 5 including CMP, GPC, fucose-GlcNac, and LNFP I. Most of these metabolites relate negatively with total peptide intensity, suggesting that higher proteolytic activity correlates with metabolic changes and a decrease in peptides from MFGM proteins.

Therefore, it seems that changes in the metabolome can explain part of the interindividual variation in the human milk proteome and peptidome. Nevertheless, these findings raise intriguing questions regarding the nature of especially the human milk peptidome and deserve further investigation.

## Conclusion

In this study, pooled human milk samples were used to investigate the interindividual variation in proteome, peptidome, and metabolome. The largest interindividual variation was observed in the peptidome (median CV 85.2%), after which follows the proteome (median CV 42.8%) and the metabolome (median CV 36.1%). Nevertheless, the majority of proteins, peptides, and metabolites show interindividual variation with a CV < 100%. With the WGCNA algorithm, 5 protein clusters and 11 peptide clusters were obtained, each with distinct characteristics. Using these WGCNA clusters, several associations were found between the data sets and with sample characteristics, giving insight into the causes of interindividual variation. Since the donating mothers in this study are generally healthy, the interindividual variation observed in this study can be considered a normal variation. The findings reported in this study can help in the interpretation of effect sizes in future omics studies since these can now be compared to the natural variability.

## Supporting Information Available

Additional results from technical replicates; additional figures on WGCNA data analysis of both proteomics and peptidomics data; boxplots showing proposed nonsecretor and Lewis negative mothers included in the study; tables with all identified proteins, peptides, and metabolites; parameters of NMR analysis (PDF)

## Notes

The mass spectrometry proteomics data have been deposited to the ProteomeXchange Consortium via the PRIDE^29^ partner repository with the data set identifier PXD028280. The mass spectrometry peptidomics data have been deposited to the ProteomeXchange Consortium via the PRIDE^29^ partner repository with the data set identifier PXD028294.

## Dedication

The authors dedicate the paper to the bright memory of Jacques J. M. Vervoort, a friend and recognized scientist, who passed away in July 2021.
