# Supplementary material for: Exploring Human Milk Dynamics: Interindividual Variation in Milk Proteome, Peptidome, and Metabolome
Source: J Proteome Res. 2022 Feb 1;21(4):1002–16. doi: 10.1021/acs.jproteome.1c00879 (PMC8981310; doi:10.1021/acs.jproteome.1c00879)
Supplement: Supplementary file 1 — pr1c00879_si_001.pdf [file pr1c00879_si_001.pdf]

Exploring human milk dynamics: Inter-individual variation in milk proteome, peptidome and metabolome

Pieter M. Dekker,[1,2] Sjef Boeren,[2] Johannes B. van Goudoever,[3] Jacques J.M. Vervoort,[2] and Kasper A. Hettinga*,[4,5]

[1] *Wageningen University & Research, Food Quality and Design Group, Bornse Weilanden 9, 6708 WG Wageningen, The Netherlands*

[2] *Wageningen University & Research, Laboratory of Biochemistry, Stippeneng 4, 6708 WE Wageningen, The Netherlands*

[3] *Amsterdam UMC Vrije Universiteit Emma Children's Hospital Department of Pediatrics, 1081 Amsterdam, The Netherlands*

[4] *Wageningen sensory evaluation techniques 5th edition pdfUniversity & Research, Food Quality and Design Group, Bornse Weilanden 9, 6708 WG Wageningen, The Netherlands*

[5] *Corresponding author*

* E-mail: kasper.hettinga@wur.nl

Phone: +31-317-48-2401

Table of contents

- Figure S1: Overall CV (%) of proteins, peptides, and metabolites plotted against CV (%) of technical replicates.
- Figure S2: Visualization of the determination of the WGCNA soft-threshold power for the proteomics data.
- Figure S3: WGCNA cluster dendrogram and overview for the proteomics data.
- Figure S4: Visualization of the determination of the WGCNA soft-threshold power for the peptidomics data.
- Figure S5: WGCNA cluster dendrogram and overview for the peptidomics data.
- Figure S6: Boxplots showing the proposed non-secretor and Lewis negative mothers.
- Table S1: Table with all identified proteins.
- Table S2: Table with all identified peptides.
- Table S3: Table with all identified metabolites.
- Listing S1: Overview with the parameters of the NMR analysis.

Overall CV (%) of proteins, peptides, and metabolites plotted against CV (%) of technical replicates

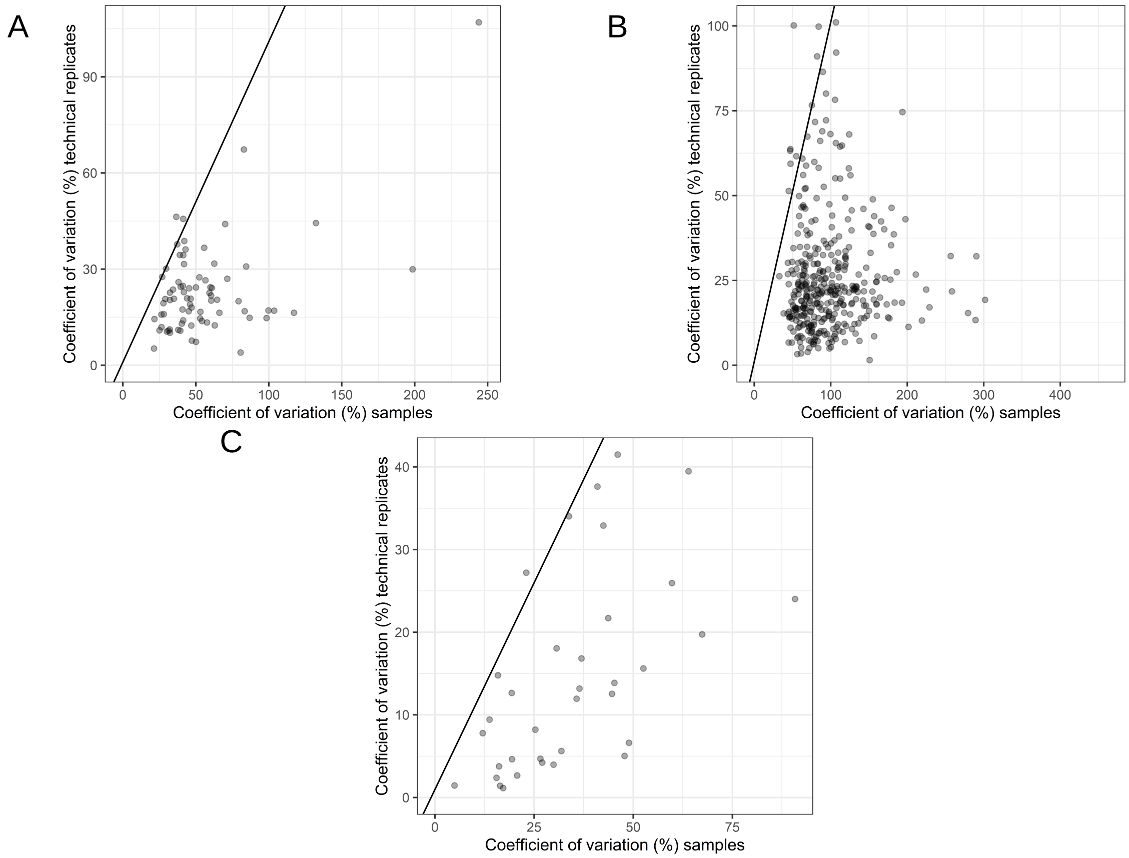

Supplemental Figure S 1: The overall CV (%) plotted against the CV (%) of the technical replicates for the proteins (A), peptides (B) and metabolites (C). The diagonal line is drawn to indicate the features for which technical CV > overall CV.

Technical replicates of proteomics data (n = 5) were used from a previous study.[1] Technical replicates of peptidomics data (n = 5) were used from a previous experiment (unpublished work, Wageningen University, 2016-05-10). Technical replicates of metabolomics data (n = 4) were analysed together with the samples used for the current study. Datafiles of all technical replicates were analysed with the same software and settings as described in the materials and methods.

Visualization of the determination of the WGCNA soft-threshold power for the proteomics data.

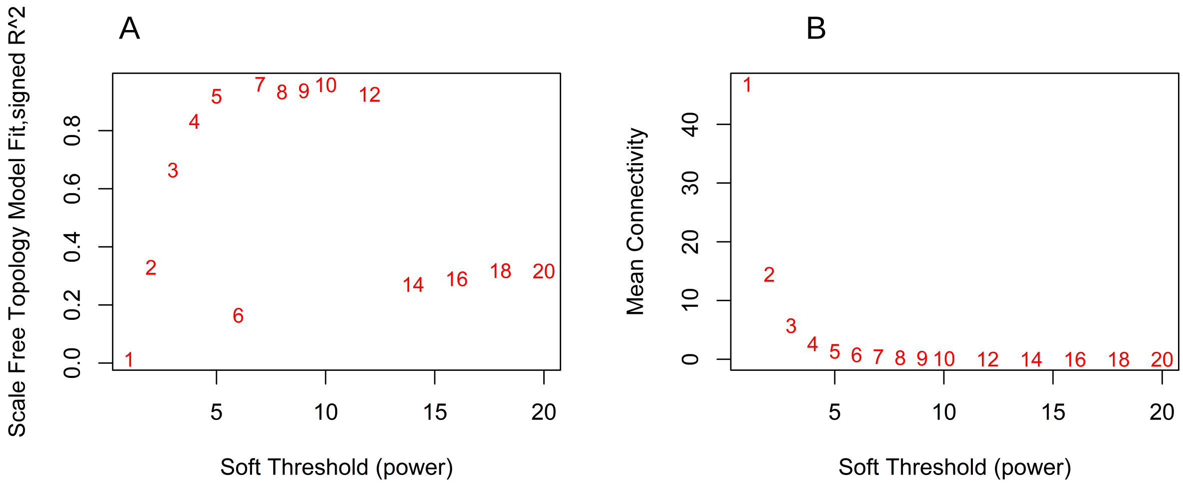

Supplemental Figure S 2: Determination of the soft-threshold power for WGCNA on proteomics data. (A) Scale-free topology fit index (y-axis) for soft-threshold values of 1-20 (x-axis). (B) Mean connectivity analysis with degree (y-axis) and soft-threshold values of 1-20 (x-axis).

WGCNA cluster dendrogram and overview for the proteomics data.

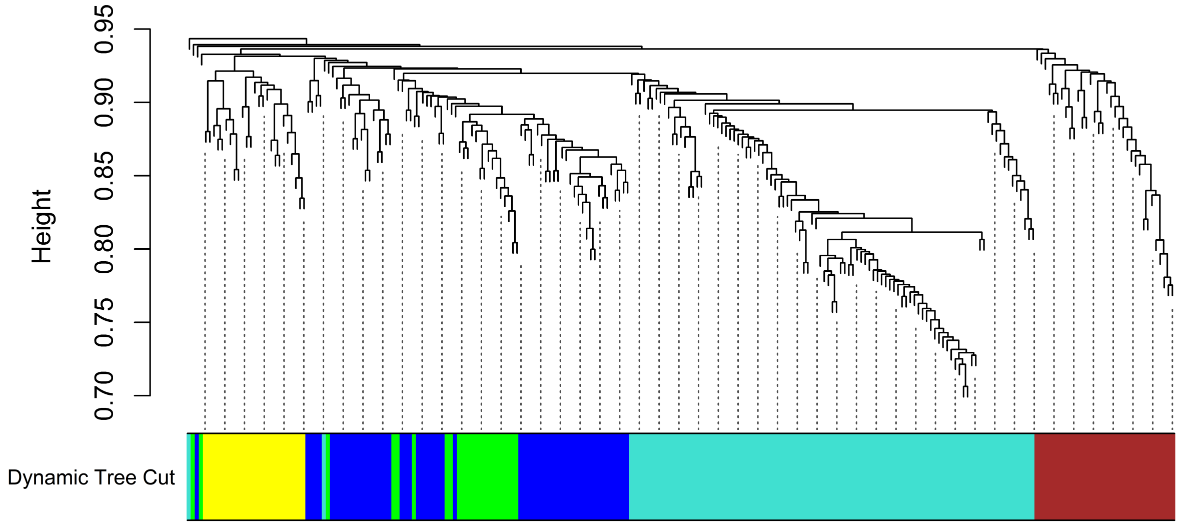

Supplemental Figure S 3: Cluster dendrogram and cluster overview for the proteomics data. Different colors indicate the different clusters, where turquoise = cluster 1, blue = cluster 2, brown = cluster 3, yellow = cluster 4 and green = cluster 5.

Visualization of the determination of the WGCNA soft-threshold power for the peptidomics data.

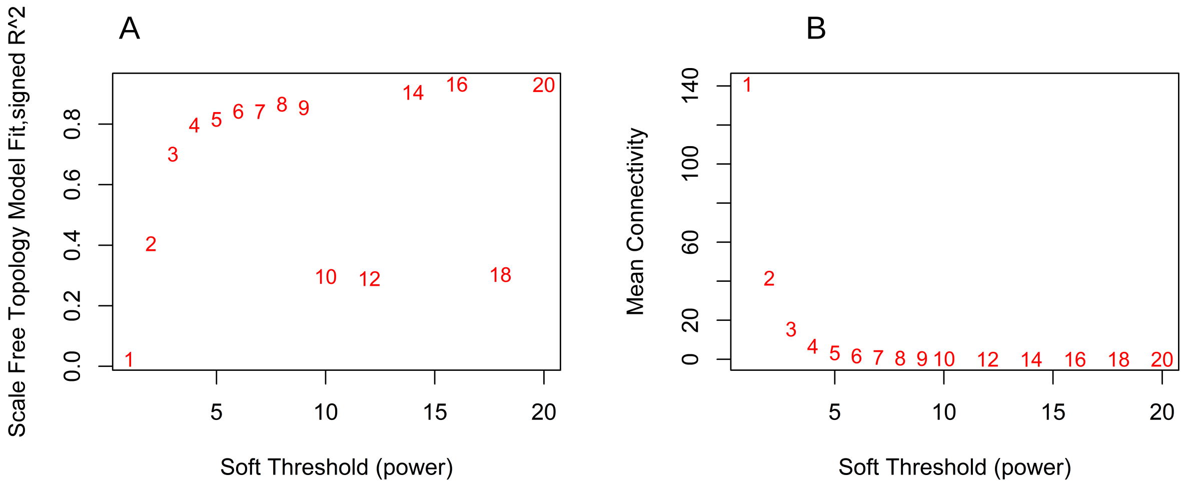

Supplemental Figure S 4: Determination of the soft-threshold power for WGCNA on peptidomics data. (A) Scale-free topology fit index (y-axis) for soft-threshold values of 1-20 (x-axis). (B) Mean connectivity analysis with degree (y-axis) and soft-threshold values of 1-20 (x-axis).

WGCNA cluster dendrogram and overview for the peptidomics data.

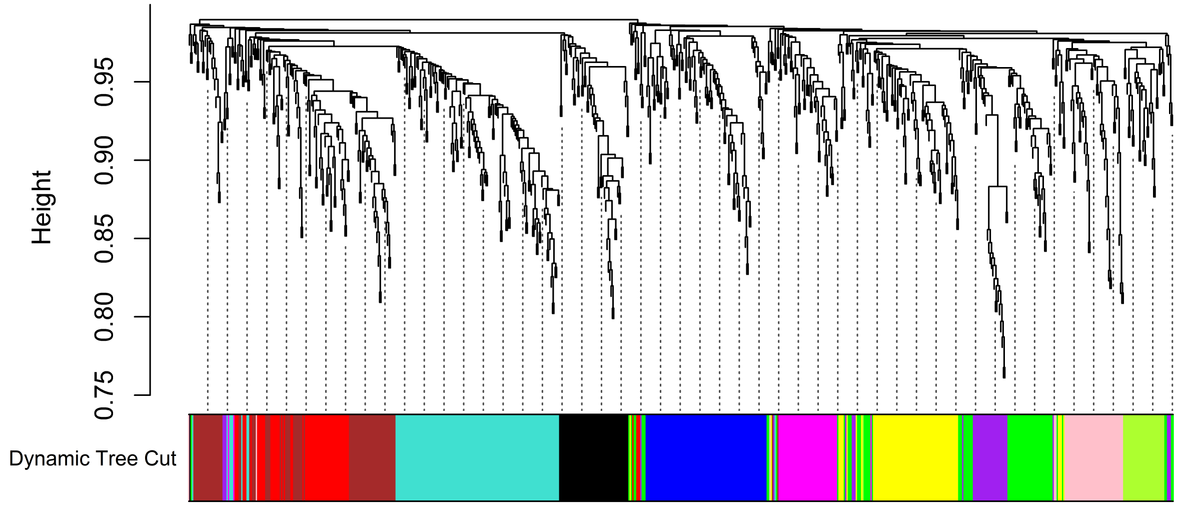

Supplemental Figure S 5: Cluster dendrogram and cluster overview for the peptidomics data. Different colors indicate the different clusters, where turquoise = cluster 1, blue = cluster 2, brown = cluster 3, yellow = cluster 4, green = cluster 5, red = cluster 6, black = cluster 7, pink = cluster 8, magenta = cluster 9, purple = cluster 10 and greenyellow = cluster 11.

Boxplots showing the proposed non-secretor and Lewis negative mothers.

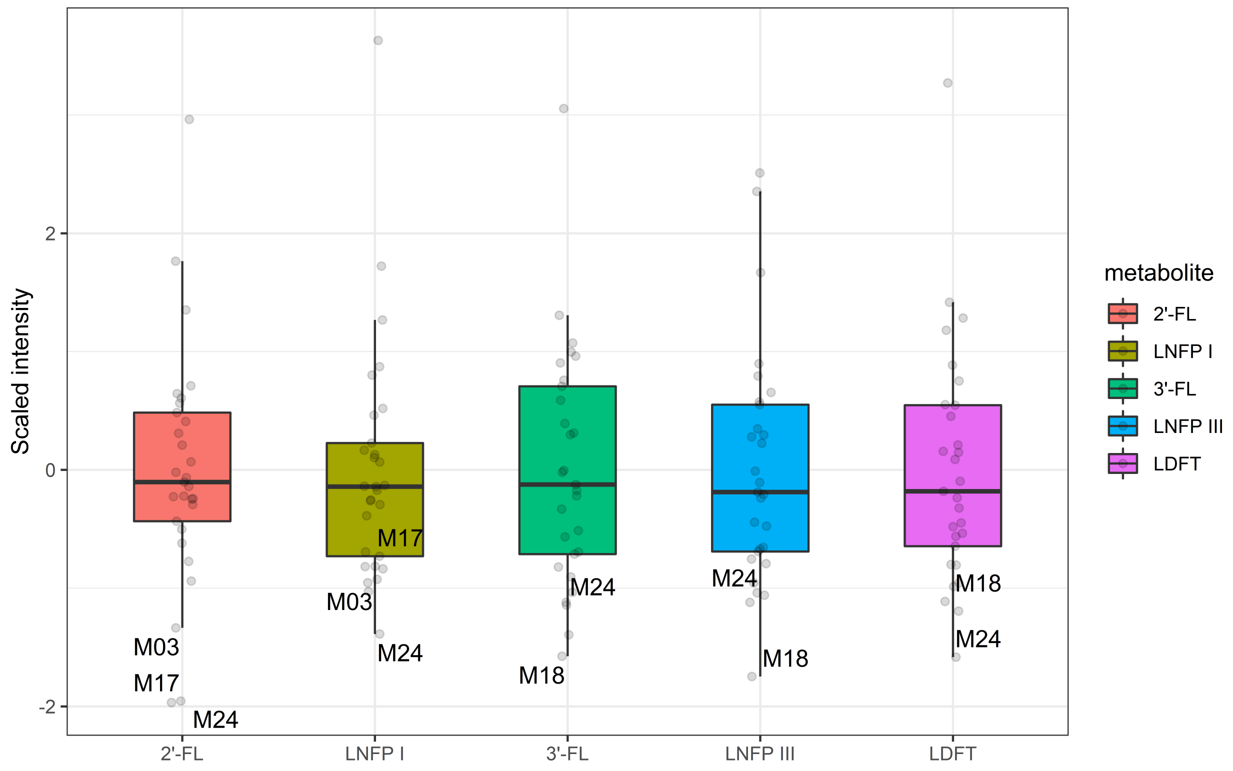

Supplemental Figure S 6: Scaled intensitites for HMOs. Labels indicate samples from mothers proposed as non-secretor (Se(-)) or Lewis negative (Le(-)).

Table with all identified proteins.

Supplemental Table S 1: List with all identified proteins, including their mean log10 LFQ intensity, inter-individual variation (CV), WGCNA cluster, sequence coverage, and identification score.

Uniprot ID	Leading protein name	Mean log10 LFQ intensity	CV (%)	WGCNA cluster	Sequence coverage (%)	Identificat-ion score
A0A193CHR0	10E8 heavy chain variable region	7.8	66.2	5	28.2	39.0
P31946	14-3-3 protein beta/alpha	7.2	39.9	1	43.1	115.1
P62258	14-3-3 protein epsilon	7.5	37.4	1	54.5	181.9
Q04917	14-3-3 protein eta	6.7	37.9	1	29.3	18.8
P61981	14-3-3 protein gamma	6.7	62.5	1	36.7	133.8
P31947	14-3-3 protein sigma	7.2	31.5	1	29.0	6.5
P63104	14-3-3 protein zeta/delta	7.9	31.3	1	63.7	323.3
B9EKV4	4-trimethylaminobutyraldehyde dehydrogenase	7.1	36.7	1	11.4	8.9
Q9BRK5	45 kDa calcium-binding protein	7.5	36.4	1	29.8	36.2
P60709	Actin	8.9	21.7	1	63.5	323.3
P07108	Acyl-CoA-binding protein	7.6	44.9	1	18.4	28.8
P02768	Albumin	10.8	21.4	2	93.9	323.3
P35747	Albumin	6.9	43.5	2	15.0	4.0
P14550	Aldo-keto reductase family 1 member A1]	7.2	34.8	1	33.8	56.4
P02763	Alpha-1-acid glycoprotein 1	8.1	41.3	1	44.8	220.0
P19652	Alpha-1-acid glycoprotein 2	6.8	60.6	2	24.4	11.4
P01011	Alpha-1-antichymotrypsin	8.9	86.9	4	47.5	323.3
P01009	Alpha-1-antitrypsin	9.2	26.4	2	67.7	323.3
P04217	Alpha-1B-glycoprotein	7.8	35.0	3	33.1	77.6
P08697	Alpha-2-antiplasmin	7.8	29.6	1	17.5	41.9
P02765	Alpha-2-HS-glycoprotein	7.8	36.6	1	25.9	58.0
P01023	Alpha-2-macroglobulin	7.5	40.3	2	11.1	30.4
O43707	Alpha-actinin-4	7.0	43.0	1	12.1	27.3
P04745	Alpha-amylase 1B	8.4	62.9	3	39.7	249.8
P06733	Alpha-enolase	8.6	28.0	1	73.0	323.3
P00709	Alpha-lactalbumin	10.8	41.9	5	54.2	323.3
P47710	Alpha-S1-casein	9.9	71.6	4	80.0	5.9

Supplemental Table S 1: List with all identified proteins, including their mean log10 LFQ intensity, inter-individual variation (CV), WGCNA cluster, sequence coverage, and identification score. *(continued)*

Uniprot ID	Leading protein name	Mean log10 LFQ intensity	CV (%)	WGCNA cluster	Sequence coverage (%)	Identificat-ion score
P05067	Amyloid-beta precursor protein	6.8	57.2	5	10.8	19.7
P01019	Angiotensinogen	6.9	42.8	2	7.7	7.3
A2KBC6	Anti-FactorVIII scFv	8.1	46.0	3	45.4	323.3
P01008	Antithrombin-III	7.5	38.1	3	23.3	26.1
P02647	Apolipoprotein A-I	8.4	41.1	1	74.2	227.3
P02652	Apolipoprotein A-II	7.8	56.6	1	40.4	28.1
P06727	Apolipoprotein A-IV	7.1	49.5	2	35.6	37.0
P04114	Apolipoprotein B-100	8.2	80.7	2	4.4	33.0
P05090	Apolipoprotein D	7.3	58.4	2	33.0	16.6
P02649	Apolipoprotein E	7.2	39.4	3	40.8	29.6
P15289	Arylsulfatase A	6.5	100.6	2	20.9	9.5
P15291	Beta-1,4-galactosyltransferase 1	7.3	44.1	2	38.4	102.1
P02749	Beta-2-glycoprotein 1GPI	7.5	47.4	2	43.1	61.0
P61769	Beta-2-microglobulin	8.8	37.3	2	58.8	89.2
P05814	Beta-casein	9.6	41.4	4	35.8	323.3
Q13410	Butyrophilin subfamily 1 member A1	8.6	47.2	4	46.4	323.3
B4E1Z4	C3/C5 convertase	7.8	61.1	2	16.0	159.1
P0DP25	Calmodulin-3	7.6	42.0	1	56.4	32.0
Q9NZT1	Calmodulin-like protein 5	7.0	77.9	1	53.4	39.2
P27797	Calreticulin	7.3	37.5	1	25.6	12.8
P22676	Calretinin	7.0	25.0	2	15.1	83.1
P00918	Carbonic anhydrase 2	6.6	41.8	2	8.1	4.6
P23280	Carbonic anhydrase 6	8.0	62.0	3	22.4	134.3
O75612	Carboxylic ester hydrolase	9.6	40.9	3	58.0	323.3
P07858	Cathepsin B	6.7	53.2	2	21.2	7.2
P07339	Cathepsin D	7.2	53.4	4	14.8	61.6
Q9UBR2	Cathepsin Z	6.4	20.5	2	7.3	7.4
E9PR17	CD59 glycoprotein	7.3	83.5	4	30.6	13.5
Q96K68	cDNA FLJ14473 fis	10.2	47.1	3	53.8	323.3
B3KU96	cDNA FLJ39433 fis	6.9	31.2	1	22.4	14.2
Q6ZW64	cDNA FLJ41552 fis	8.1	117.2	3	52.0	58.2

Supplemental Table S 1: List with all identified proteins, including their mean log10 LFQ intensity, inter-individual variation (CV), WGCNA cluster, sequence coverage, and identification score. *(continued)*

Uniprot ID	Leading protein name	Mean log10 LFQ intensity	CV (%)	WGCNA cluster	Sequence coverage (%)	Identification score
Q6ZVX0	cDNA FLJ41981 fis	8.5	62.5	3	53.2	164.8
B4DPP8	cDNA FLJ53075	7.7	39.6	3	22.2	68.6
P29373	Cellular retinoic acid-binding protein 2	6.8	38.9	1	39.9	7.1
P00450	Ceruloplasmin	7.8	27.6	2	25.8	55.2
A0A2P9AAP7	Chaperone protein DnaK	7.1	35.5	2	4.9	2.6
P36222	Chitinase-3-like protein 1	6.8	122.8	2	21.4	15.3
O00299	Chloride intracellular channel protein 1	6.8	31.0	1	33.6	14.6
Q6WN34	Chordin-like protein 2	8.3	50.1	3	25.1	86.8
H0YCU0	Chordin-like protein 2	7.5	42.4	3	30.8	11.7
P10909	Clusterin	9.3	62.6	4	52.6	323.3
P23528	Cofilin-1	7.2	40.4	2	62.5	42.3
A2NB45	Cold agglutinin FS-1 L-chain	7.3	81.5	2	38.9	6.1
P01024	Complement C3	9.0	41.3	4	56.6	323.3
P0C0L5	Complement C4-B	8.4	64.6	4	44.3	323.3
P02748	Complement component C9	6.7	56.1	1	9.7	13.5
P08603	Complement factor H	6.5	46.5	2	4.5	12.8
P08185	Corticosteroid-binding globulin	7.0	33.0	2	5.7	3.3
P04080	Cystatin-B	7.1	42.0	1	55.1	22.4
P01034	Cystatin-C	8.2	50.5	5	37.0	35.6
P00171	Cytochrome b5	6.4	49.1	2	25.4	4.1
P21399	Cytoplasmic aconitate hydratase	7.0	43.5	1	8.8	16.1
P28838	Cytosol aminopeptidase	6.7	40.2	1	11.9	12.2
Q96KP4	Cytosolic non-specific dipeptidase	8.0	38.0	1	38.7	54.7
E1B7J1	Deleted.	6.8	35.1	2	5.9	4.6
P81605	Dermcidin	6.8	70.1	1	22.7	6.1
Q02487	Desmocollin-2	7.6	57.4	2	2.6	6.9
Q16555	Dihydropyrimidinase-related protein 2	6.6	44.2	5	10.5	82.4
Q14118	Dystroglycan	7.3	47.7	1	5.0	14.5
P13639	Elongation factor 2	6.9	42.6	1	6.2	10.0

Supplemental Table S 1: List with all identified proteins, including their mean log10 LFQ intensity, inter-individual variation (CV), WGCNA cluster, sequence coverage, and identification score. *(continued)*

Uniprot ID	Leading protein name	Mean log10 LFQ intensity	CV (%)	WGCNA cluster	Sequence coverage (%)	Identificat-ion score
P11021	Endoplasmic reticulum chaperone BiP	7.9	28.2	1	33.8	124.0
P14625	Endoplasmin	7.5	51.1	1	23.1	40.3
Q15375	Ephrin type-A receptor 7	6.5	52.2	2	3.8	7.5
V9HW68	Epididymis luminal protein 214	8.7	42.9	5	41.5	166.4
V9HWJ7	Epididymis secretory protein Li 37	6.8	39.5	1	13.7	9.0
Q08345	Epithelial discoidin domain-containing receptor 1	7.1	40.2	2	7.4	48.7
P15311	Ezrin	7.8	34.4	1	30.4	103.9
P05413	Fatty acid-binding protein	9.3	45.1	1	95.5	323.3
P49327	Fatty acid synthase S-acetyltransferase	8.4	63.0	1	33.9	323.3
P02671	Fibrinogen alpha chain	7.3	57.8	2	10.4	51.2
P02675	Fibrinogen beta chain	6.9	35.4	2	20.9	26.1
Q5D862	Filaggrin-2	6.9	27.9	2	3.0	67.6
P15328	Folate receptor alpha	7.0	49.2	5	12.6	22.3
Q12841	Follistatin-related protein 1	8.0	80.6	1	47.4	323.3
P04075	Fructose-bisphosphate aldolase A	8.3	38.0	1	45.1	225.7
Q08380	Galectin-3-binding protein	8.4	32.3	3	26.5	323.3
V9HWI6	Gc-globulin	8.5	32.3	1	53.8	281.1
A0A0X9T7V9	GCT-A4 light chain variable region	7.7	71.7	5	39.4	9.1
A0A0X9T0H6	GCT-A5 heavy chain variable region	8.0	66.7	4	17.5	57.0
A0A0X9UWL5	GCT-A5 light chain variable region	7.4	71.3	5	46.9	95.6
A0A125QYY5	GCT-A9 light chain variable region	7.2	59.8	5	31.8	153.0
P06396	Gelsolin	8.0	34.4	3	27.0	68.1
P06744	Glucose-6-phosphate isomerase	6.8	27.1	1	7.9	7.3
P14314	Glucosidase 2 subunit beta	7.7	37.4	1	12.9	44.8
P48637	Glutathione synthetase	7.2	34.1	1	16.4	28.3
P04406	Glyceraldehyde-3-phosphate dehydrogenase	7.8	43.3	1	31.9	20.2
Q8NBJ4	Golgi membrane protein 1	7.1	44.0	5	20.4	17.1

Supplemental Table S 1: List with all identified proteins, including their mean log10 LFQ intensity, inter-individual variation (CV), WGCNA cluster, sequence coverage, and identification score. *(continued)*

Uniprot ID	Leading protein name	Mean log10 LFQ intensity	CV (%)	WGCNA cluster	Sequence coverage (%)	Identification score
Q9HAV0	Guanine nucleotide-binding protein subunit beta-4	6.6	70.2	1	10.0	8.8
P00738	Haptoglobin	9.2	52.4	2	52.5	261.7
P17066	Heat shock 70 kDa protein 6	7.2	33.3	1	14.6	13.7
P11142	Heat shock cognate 71 kDa protein	7.8	32.3	1	28.5	117.9
P40292	Heat shock protein 90	7.2	49.1	2	5.9	84.9
P04792	Heat shock protein beta-1	7.0	43.9	1	41.5	10.2
P07900	Heat shock protein HSP 90-alpha	8.0	46.9	1	24.6	224.0
P08238	Heat shock protein HSP 90-beta	7.2	39.8	1	25.4	23.5
Q6LAM1	Heavy chain of factor I	6.9	70.7	2	18.4	23.4
Q9NRV9	Heme-binding protein 1	6.6	39.9	1	23.3	4.9
P02790	Hemopexin	7.3	34.0	2	20.6	32.8
Q86YZ3	Hornerin	7.9	243.9	1	13.9	208.2
A0A125QYY9	IBM-B2 heavy chain variable region	7.5	87.8	1	30.9	25.8
S6B291	IgG H chain	6.7	67.8	5	35.6	16.5
S6AWF4	IgG L chain	7.8	35.0	3	31.1	5.1
Q6PIL8	IGK@ protein	9.5	43.6	3	65.3	323.3
Q6IN99	IGL@ protein	8.7	47.4	3	52.6	5.3
Q0KKI6	Immunoblobulin light chain	7.5	60.6	5	63.6	91.5
P0DOX2	Immunoglobulin alpha-2 heavy chain	7.5	87.3	4	47.0	31.1
Q9NPP6	Immunoglobulin heavy chain variant	7.0	98.5	5	59.6	13.6
P01861	Immunoglobulin heavy constant gamma 4	7.6	76.5	4	24.7	62.2
P01871	Immunoglobulin heavy constant mu	8.1	198.6	3	37.8	107.2
P01591	Immunoglobulin J chain	9.4	55.8	3	81.1	148.8
P0DOX8	Immunoglobulin lambda-1 light chain	8.0	53.2	3	47.7	183.0
Q15181	Inorganic pyrophosphatase	6.7	25.8	2	22.1	11.1
P18065	Insulin-like growth factor-binding protein 2	7.8	32.3	3	39.4	149.6
P19827	Inter-alpha-trypsin inhibitor heavy chain H1	7.3	120.2	2	11.3	20.3

Supplemental Table S 1: List with all identified proteins, including their mean log10 LFQ intensity, inter-individual variation (CV), WGCNA cluster, sequence coverage, and identification score. *(continued)*

Uniprot ID	Leading protein name	Mean log10 LFQ intensity	CV (%)	WGCNA cluster	Sequence coverage (%)	Identification score
P19823	Inter-alpha-trypsin inhibitor heavy chain H2	7.3	36.2	2	9.3	26.5
Q14624	Inter-alpha-trypsin inhibitor heavy chain H4	8.2	37.4	1	16.1	38.5
O75874	Isocitrate dehydrogenase cytoplasmic-specific ICDH	7.4	37.5	1	22.9	26.5
Q92876	Kallikrein-6	7.8	57.6	5	29.9	41.6
P07498	Kappa-casein	8.5	82.9	4	53.3	57.2
O43278	Kunitz-type protease inhibitor 1	7.0	41.5	2	15.3	7.1
P07195	L-lactate dehydrogenase B chain	8.1	44.0	1	36.5	78.3
Q08431	Lactadherin	7.8	60.4	4	44.6	51.7
P22079	Lactoperoxidase	7.8	84.5	1	25.0	42.0
P02788	Lactotransferrin	10.9	45.4	3	89.8	57.1
Q99538	Legumain	6.9	42.8	1	17.5	9.7
P02750	Leucine-rich alpha-2-glycoprotein	8.4	26.9	3	38.2	59.3
Q86X29	Lipolysis-stimulated lipoprotein receptor	6.5	21.3	2	4.3	7.3
P06858	Lipoprotein lipase	7.4	99.8	4	24.3	61.7
P61626	Lysozyme C	8.4	132.3	4	70.9	63.9
P22897	Macrophage mannose receptor 1	9.3	30.2	2	42.9	323.3
P40925	Malate dehydrogenase	7.5	30.8	1	37.6	95.5
P49006	MARCKS-related protein	7.0	67.4	3	14.1	31.2
P01033	Metalloproteinase inhibitor 1	6.9	29.8	3	32.2	7.1
Q13228	Methanethiol oxidase	7.0	39.7	1	22.5	38.4
P26038	Moesin	7.1	22.2	1	21.3	13.0
P08571	Monocyte differentiation antigen CD14	9.2	29.1	3	47.2	323.3
P15941	Mucin-1	7.5	56.5	4	36.2	18.9
A0A0G2JQJ2	Mucin-4	7.3	74.7	4	9.6	40.3
Q9UL85	Myosin-reactive immunoglobulin kappa chain variable region	7.3	64.9	2	24.8	218.0

Supplemental Table S 1: List with all identified proteins, including their mean log10 LFQ intensity, inter-individual variation (CV), WGCNA cluster, sequence coverage, and identification score. *(continued)*

Uniprot ID	Leading protein name	Mean log10 LFQ intensity	CV (%)	WGCNA cluster	Sequence coverage (%)	Identificat-ion score
Q9UL78	Myosin-reactive immunoglobulin light chain variable region	8.1	41.9	5	65.1	323.3
Q9UL82	Myosin-reactive immunoglobulin light chain variable region	7.2	49.0	5	28.0	4.4
P29966	Myristoylated alanine-rich C-kinase substrate	7.9	51.3	1	14.8	25.7
Q7Z3X3	N-acetylglucosamine-6-sulfatase	7.3	57.6	1	14.3	18.7
O94760	N,N-dimethylarginine dimethylaminohydrolase 1	6.9	28.8	1	11.2	35.9
O14745	Na/H exchange regulatory cofactor NHE-RF1/H exchanger	7.5	34.3	1	38.8	41.8
P43490	Nicotinamide phosphoribosyltransferase	6.9	44.9	2	16.7	8.7
P61916	NPC intracellular cholesterol transporter 2	7.4	53.0	1	39.3	61.0
Q02818	Nucleobindin-1	8.1	41.8	1	56.0	121.1
P80303	Nucleobindin-2	8.4	39.0	1	56.2	302.0
Q9H173	Nucleotide exchange factor SIL1	6.5	26.4	1	10.3	7.8
P10451	Osteopontin	9.8	42.1	1	66.4	323.3
Q99497	Parkinson disease protein 7	7.6	30.8	1	43.2	19.8
P62937	Peptidyl-prolyl cis-trans isomerase A	7.9	33.7	2	43.6	49.1
P23284	Peptidyl-prolyl cis-trans isomerase B	7.5	32.1	5	29.3	17.8
Q99541	Perilipin-2	7.7	61.0	4	43.9	51.7
O60664	Perilipin-3	7.5	36.8	1	36.7	141.9
Q06830	Peroxiredoxin-1	7.7	32.3	1	49.7	25.4
P32119	Peroxiredoxin-2	7.2	36.8	1	39.9	10.5
P30041	Peroxiredoxin-6	6.6	39.7	1	21.9	9.9
P13696	Phosphatidylethanolamine-binding protein 1	7.3	39.2	1	34.2	12.6
P36871	Phosphoglucomutase-1	7.7	43.7	1	31.5	109.1
P00558	Phosphoglycerate kinase 1	7.5	41.3	1	30.0	53.8
P36955	Pigment epithelium-derived factor	6.9	30.1	1	17.2	16.9

Supplemental Table S 1: List with all identified proteins, including their mean log10 LFQ intensity, inter-individual variation (CV), WGCNA cluster, sequence coverage, and identification score. *(continued)*

Uniprot ID	Leading protein name	Mean log10 LFQ intensity	CV (%)	WGCNA cluster	Sequence coverage (%)	Identificat-ion score
P05155	Plasma protease C1 inhibitor	7.5	35.1	2	11.7	19.4
P16671	Platelet glycoprotein 4	7.2	56.8	4	13.8	9.8
O00592	Podocalyxin	7.2	79.3	4	6.6	259.5
P01833	Polymeric immunoglobulin receptor	10.3	25.1	3	61.9	323.3
P01133	Pro-epidermal growth factor	7.5	34.3	3	9.8	54.9
P07737	Profilin-1	6.8	36.8	2	38.6	56.6
P07602	Prosaposin	8.1	60.0	4	33.8	87.5
P02760	Protein AMBP	6.9	38.3	2	7.4	6.6
P07237	Protein disulfide-isomerase	7.9	35.2	1	39.8	144.9
P30101	Protein disulfide-isomerase A3	7.1	48.4	1	31.7	38.2
P13667	Protein disulfide-isomerase A4	7.0	42.8	1	12.4	12.5
P23297	Protein S100-A1	7.0	80.8	1	26.6	67.2
P00734	Prothrombin	6.9	19.6	2	12.7	11.5
P50395	Rab GDP dissociation inhibitor beta	7.6	30.0	1	32.1	121.5
P62491	Ras-related protein Rab-11A	6.6	30.7	1	28.4	6.2
Q9NP72	Ras-related protein Rab-18	6.7	35.2	2	16.0	14.8
P62820	Ras-related protein Rab-1A	7.0	34.5	1	32.4	97.7
P51149	Ras-related protein Rab-7a	6.8	39.4	1	26.9	87.2
A0N5G5	Rheumatoid factor D5 light chain	7.7	56.4	5	28.0	79.1
A2J1M8	Rheumatoid factor RF-IP12	7.7	59.3	3	21.1	22.6
P52565	Rho GDP-dissociation inhibitor 1	7.6	41.5	1	43.1	33.8
P02787	Serotransferrin	8.5	35.0	2	56.4	323.3
P55854	Small ubiquitin-related modifier 3	6.6	55.4	2	17.0	9.5
P27105	Stomatin	6.7	205.5	4	31.6	31.0
O00391	Sulfhydryl oxidase 1	7.7	46.5	3	23.6	200.9
Q99536	Synaptic vesicle membrane protein VAT-1 homolog	6.8	36.9	2	10.7	7.1
P31431	Syndecan-4	7.7	41.9	3	5.6	14.2
P24821	Tenascin	7.7	246.8	5	19.9	323.3
P10599	Thioredoxin	6.9	67.5	2	31.4	9.7
P04066	Tissue alpha-L-fucosidase	7.0	83.0	4	7.5	25.6

Supplemental Table S 1: List with all identified proteins, including their mean log10 LFQ intensity, inter-individual variation (CV), WGCNA cluster, sequence coverage, and identification score. *(continued)*

Uniprot ID	Leading protein name	Mean log10 LFQ intensity	CV (%)	WGCNA cluster	Sequence coverage (%)	Identificat-ion score
Q8WZ42	Titin	9.3	56.7	1	0.8	18.8
O43493	Trans-Golgi network integral membrane protein 2	7.0	32.0	1	10.6	94.4
P20061	Transcobalamin-1	7.8	103.7	4	20.8	92.2
P55072	Transitional endoplasmic reticulum ATPase-ATPase p97 subunit	7.7	69.0	5	5.8	4.8
Q53EM5	Transketolase variant	7.9	59.4	1	29.4	30.8
P60174	Triosephosphate isomerase	7.7	27.2	1	66.7	99.4
Q1ZYL5	Tropomyosin 1 alpha variant 6	6.5	46.6	1	33.1	40.3
Q5VU61	Tropomyosin alpha-3 chain	7.3	57.3	1	54.3	20.9
O75347	Tubulin-specific chaperone A	6.8	28.0	2	35.7	4.9
P62987	Ubiquitin-60S ribosomal protein L40	6.8	16.2	2	67.2	13.7
P22314	Ubiquitin-like modifier-activating enzyme 1	7.0	38.8	1	11.2	162.7
Q8NEJ1	Uncharacterized protein	10.0	50.1	3	52.1	323.3
Q16851	UTP–glucose-1-phosphate uridylyltransferase	8.5	40.4	1	54.5	155.9
P02774	Vitamin D-binding protein	7.6	47.3	1	48.5	5.7
P04004	Vitronectin	7.9	27.9	3	21.5	38.6
P47989	Xanthine dehydrogenase/oxidase	9.4	26.4	1	57.5	323.3
P25311	Zinc-alpha-2-glycoprotein	8.2	54.2	1	50.7	63.6

Table with all identified peptides.

Supplemental Table S 2: List with all identified peptides, including their precursor protein, sequence range, mean log10 LFQ intensity, inter-individual variation (CV), WGCNA cluster, and identification score.

Sequence	Uniprot ID	Leading protein name	Sequence positions	Mean log10 LFQ intensity	CV (%)	WGCNA cluster	Identification score
KVPQLEIVPN	P02662	Alpha-S1-casein	120-129	6.3	57.4	8	96.3
RPKLPLRYPE	P47710	Alpha-S1-casein	16-25	7.3	133.2	3	115.6
RPKLPLRYPER	P47710	Alpha-S1-casein	16-26	5.6	156.4	3	67.2
RPKLPLRYPERL	P47710	Alpha-S1-casein	16-27	6.2	91.0	1	72.3
RPKLPLRYPERLQ	P47710	Alpha-S1-casein	16-28	6.7	73.0	3	135.0
RPKLPLRYPERLQNP	P47710	Alpha-S1-casein	16-30	6.4	75.5	6	72.9
RPKLPLRYPERLQNPS	P47710	Alpha-S1-casein	16-31	6.1	60.1	3	84.9
RPKLPLRYPERLQNPSE	P47710	Alpha-S1-casein	16-32	7.5	63.2	3	179.1
RPKLPLRYPERLQNPSESSEPIP	P47710	Alpha-S1-casein	16-38	6.5	99.3	3	76.0
RPKLPLRYPERLQNPSESSEPIPLE	P47710	Alpha-S1-casein	16-40	6.5	68.0	7	131.6
SDISNPTAHENYEKNNVMLQW	P47710	Alpha-S1-casein	165-185	6.1	290.3	2	148.4
DISNPTAHENYEKNN	P47710	Alpha-S1-casein	166-180	5.5	45.1	2	77.1
DISNPTAHENYEKNNV	P47710	Alpha-S1-casein	166-181	5.4	92.4	2	73.2
DISNPTAHENYEKNNVMLQW	P47710	Alpha-S1-casein	166-185	5.8	63.2	4	103.8
SNPTAHENYEKNNV	P47710	Alpha-S1-casein	168-181	6.0	182.5	2	191.8
NPTAHENYEKNNV	P47710	Alpha-S1-casein	169-181	5.8	146.4	10	136.5
NPTAHENYEKNNVML	P47710	Alpha-S1-casein	169-183	5.8	156.8	2	51.8
HENYEKNNVML	P47710	Alpha-S1-casein	173-183	6.2	78.6	2	140.1
ENYEKNNVML	P47710	Alpha-S1-casein	174-183	5.5	77.5	4	78.1
NYEKNNVML	P47710	Alpha-S1-casein	175-183	6.2	52.1	2	96.3
KLPLRYPERLQNPSE	P47710	Alpha-S1-casein	18-32	5.9	75.4	3	68.8
TDAPSFSDIPNPI	P02662	Alpha-S1-casein	189-201	6.0	55.6	8	155.2
LPLRYPERLQNPSE	P47710	Alpha-S1-casein	19-32	6.6	68.3	6	139.0
YPERLQNPSESSEPIP	P47710	Alpha-S1-casein	23-38	6.4	45.7	1	116.0
RLQNPSESSEPIP	P47710	Alpha-S1-casein	26-38	6.7	59.2	7	140.1
RLQNPSESSEPIPL	P47710	Alpha-S1-casein	26-39	6.2	146.3	2	108.7
RLQNPSESSEPIPLE	P47710	Alpha-S1-casein	26-40	6.8	70.7	7	168.9
RLQNPSESSEPIPLESREE	P47710	Alpha-S1-casein	26-44	5.7	32.9	3	83.3
RLQNPSESSEPIPLESREEYMNGMN	P47710	Alpha-S1-casein	26-50	8.1	63.7	7	258.5
LQNPSESSEPIPLESREEYMNGMN	P47710	Alpha-S1-casein	27-50	7.1	67.9	7	224.3

Supplemental Table S 2: List with all identified peptides, including their precursor protein, sequence range, mean log10 LFQ intensity, inter-individual variation (CV), WGCNA cluster, and identification score. *(continued)*

Sequence	Uniprot ID	Leading protein name	Sequence positions	Mean log10 LFQ intensity	CV (%)	WGCNA cluster	Identification score
NPSESSEPIPLESRE	P47710	Alpha-S1-casein	29-43	5.5	58.7	9	80.5
NPSESSEPIPLESREE	P47710	Alpha-S1-casein	29-44	5.9	88.9	1	204.1
NPSESSEPIPLESREEYMNGMN	P47710	Alpha-S1-casein	29-50	6.6	183.5	1	295.0
NPSESSEPIPLESREEYMNGMNR	P47710	Alpha-S1-casein	29-51	6.2	157.5	7	97.5
ESSEPIPLESREEYMNGMNR	P47710	Alpha-S1-casein	32-51	5.9	58.5	7	56.9
SSEPIPLESREEYMNGMN	P47710	Alpha-S1-casein	33-50	6.7	68.5	7	220.2
SSEPIPLESREEYMNGMNR	P47710	Alpha-S1-casein	33-51	6.3	95.0	7	64.5
SEPIPLESREE	P47710	Alpha-S1-casein	34-44	5.7	53.3	7	76.8
SEPIPLESREEYMNGMN	P47710	Alpha-S1-casein	34-50	6.1	84.0	1	160.2
EPIPLESREEYMNGMN	P47710	Alpha-S1-casein	35-50	6.3	154.5	1	213.4
FVAPFPEVFGK	P02662	Alpha-S1-casein	39-49	5.4	68.6	6	61.0
ESREEYMNGMNR	P47710	Alpha-S1-casein	40-51	5.4	143.9	3	65.5
SREEYMNGMNR	P47710	Alpha-S1-casein	41-51	4.9	109.1	2	86.0
QRNILREKQTDEIKDT	P47710	Alpha-S1-casein	52-67	5.7	76.6	6	111.0
QRNILREKQTDEIKDTR	P47710	Alpha-S1-casein	52-68	6.8	170.0	6	89.5
NILREKQTDEIK	P47710	Alpha-S1-casein	54-65	5.8	170.6	1	136.3
NILREKQTDEIKDT	P47710	Alpha-S1-casein	54-67	6.1	65.9	6	204.3
NILREKQTDEIKDTR	P47710	Alpha-S1-casein	54-68	7.3	63.9	3	186.8
REKQTDEIKDT	P47710	Alpha-S1-casein	57-67	5.1	107.9	3	114.8
EKQTDEIKDT	P47710	Alpha-S1-casein	58-67	5.6	71.0	6	232.4
EKQTDEIKDTR	P47710	Alpha-S1-casein	58-68	7.1	106.7	3	252.3
KQTDEIKDTR	P47710	Alpha-S1-casein	59-68	5.2	175.4	3	82.4
HIQKEDVPSER	P02662	Alpha-S1-casein	95-105	5.8	101.0	8	122.7
EVPKAKDTVYT	P05814	Beta-casein	100-110	7.3	47.1	3	188.9
EVPKAKDTVYTK	P05814	Beta-casein	100-111	6.3	61.9	3	104.0
EVPKAKDTVYTKG	P05814	Beta-casein	100-112	6.3	100.5	2	103.8
EVPKAKDTVYTKGR	P05814	Beta-casein	100-113	5.7	84.1	2	96.6
EVPKAKDTVYTKGRVMP	P05814	Beta-casein	100-116	5.7	81.1	2	54.3
VPKAKDTVYT	P05814	Beta-casein	101-110	6.8	107.5	10	186.7
VPKAKDTVYTK	P05814	Beta-casein	101-111	5.7	99.6	3	124.4
VPKAKDTVYTKG	P05814	Beta-casein	101-112	5.7	122.5	2	132.8
VPKAKDTVYTKGRVM	P05814	Beta-casein	101-115	5.4	85.5	4	65.1

Supplemental Table S 2: List with all identified peptides, including their precursor protein, sequence range, mean log10 LFQ intensity, inter-individual variation (CV), WGCNA cluster, and identification score. *(continued)*

Sequence	Uniprot ID	Leading protein name	Sequence positions	Mean log10 LFQ intensity	CV (%)	WGCNA cluster	Identification score
AKDTVYTKGR	P05814	Beta-casein	104-113	6.4	114.5	3	256.9
AKDTVYTKGRV	P05814	Beta-casein	104-114	5.6	79.5	3	127.2
AKDTVYTKGRVMP	P05814	Beta-casein	104-116	6.8	120.2	3	182.0
AKDTVYTKGRVMPV	P05814	Beta-casein	104-117	5.8	201.7	2	142.8
AKDTVYTKGRVMPVL	P05814	Beta-casein	104-118	7.1	104.7	3	227.6
AKDTVYTKGRVMPVLK	P05814	Beta-casein	104-119	7.9	83.6	3	217.1
AKDTVYTKGRVMPVLKSPTIP	P05814	Beta-casein	104-124	6.9	219.3	2	99.9
KDTVYTKGRVMP	P05814	Beta-casein	105-116	6.1	131.2	6	122.4
KDTVYTKGRVMPV	P05814	Beta-casein	105-117	5.5	95.1	3	73.0
KDTVYTKGRVMPVL	P05814	Beta-casein	105-118	6.9	73.0	3	122.3
KDTVYTKGRVMPVLK	P05814	Beta-casein	105-119	6.9	119.3	3	156.2
DTVYTKGR	P05814	Beta-casein	106-113	6.9	127.9	3	183.7
DTVYTKGRV	P05814	Beta-casein	106-114	6.4	93.6	1	205.7
DTVYTKGRVMP	P05814	Beta-casein	106-116	6.5	78.6	3	140.8
DTVYTKGRVMPVL	P05814	Beta-casein	106-118	7.7	59.9	6	199.7
DTVYTKGRVMPVLK	P05814	Beta-casein	106-119	8.3	77.5	3	204.5
DTVYTKGRVMPVLKSPTIP	P05814	Beta-casein	106-124	6.7	160.4	6	107.6
DTVYTKGRVMPVLKSPTIPF	P05814	Beta-casein	106-125	6.1	258.4	3	126.2
TVYTKGRVMPVL	P05814	Beta-casein	107-118	6.5	98.9	6	131.0
TVYTKGRVMPVLK	P05814	Beta-casein	107-119	6.1	203.4	6	76.5
VYTKGRVMPVLK	P05814	Beta-casein	108-119	5.6	130.6	3	72.7
YTKGRVMPVL	P05814	Beta-casein	109-118	5.5	63.7	3	85.1
YTKGRVMPVLK	P05814	Beta-casein	109-119	6.3	106.0	3	96.2
TKGRVMPVLKSPTIP	P05814	Beta-casein	110-124	5.8	176.7	2	55.7
GRVMPVLK	P05814	Beta-casein	112-119	7.9	102.1	3	175.8
GRVMPVLKSPTIP	P05814	Beta-casein	112-124	8.3	127.2	2	89.3
GRVMPVLKSPTIPF	P05814	Beta-casein	112-125	7.2	289.3	2	121.9
GRVMPVLKSPTIPFFD	P05814	Beta-casein	112-127	6.7	140.7	6	170.0
GRVMPVLKSPTIPFFDPQIP	P05814	Beta-casein	112-131	6.6	188.6	6	69.1
GRVMPVLKSPTIPFFDPQIPK	P05814	Beta-casein	112-132	7.2	100.5	6	135.7
GRVMPVLKSPTIPFFDPQIPKLTD	P05814	Beta-casein	112-135	6.2	130.8	1	110.2
RVMPVLKSPT	P05814	Beta-casein	113-122	5.7	155.8	3	85.7
RVMPVLKSPTIP	P05814	Beta-casein	113-124	6.0	127.8	1	67.2

Supplemental Table S 2: List with all identified peptides, including their precursor protein, sequence range, mean log10 LFQ intensity, inter-individual variation (CV), WGCNA cluster, and identification score. *(continued)*

Sequence	Uniprot ID	Leading protein name	Sequence posi-tions	Mean log10 LFQ in-tensity	CV (%)	WGCNA cluster	Identificat-ion score
VMPVLKSPTIP	P05814	Beta-casein	114-124	7.0	183.6	2	120.1
VMPVLKSPTIPF	P05814	Beta-casein	114-125	5.9	301.8	5	87.9
VMPVLKSPTIPFFD	P05814	Beta-casein	114-127	5.8	195.1	2	120.1
VMPVLKSPTIPFFDPQIPK	P05814	Beta-casein	114-132	6.3	89.7	6	94.0
MPVLKSPT	P05814	Beta-casein	115-122	6.0	101.4	3	127.1
MPVLKSPTIP	P05814	Beta-casein	115-124	7.3	74.0	1	103.8
MPVLKSPTIPFFD	P05814	Beta-casein	115-127	5.9	108.5	6	154.2
MPVLKSPTIPFFDPQIP	P05814	Beta-casein	115-131	7.3	108.0	6	156.2
MPVLKSPTIPFFDPQIPK	P05814	Beta-casein	115-132	6.7	126.2	1	159.8
PVLKSPTIPFFDPQIP	P05814	Beta-casein	116-131	5.7	126.3	6	94.1
VLKSPTIPFFDPQIP	P05814	Beta-casein	117-131	5.7	114.9	6	88.2
VLKSPTIPFFDPQIPK	P05814	Beta-casein	117-132	5.6	78.1	6	123.0
KSPTIPFFDPQIP	P05814	Beta-casein	119-131	5.6	97.6	6	71.0
SPTIPFFDPQ	P05814	Beta-casein	120-129	5.3	40.6	6	69.4
SPTIPFFDPQIP	P05814	Beta-casein	120-131	6.5	140.8	6	90.6
SPTIPFFDPQIPK	P05814	Beta-casein	120-132	6.7	118.5	6	148.3
SPTIPFFDPQIPKL	P05814	Beta-casein	120-133	5.6	312.3	1	123.6
SPTIPFFDPQIPKLT	P05814	Beta-casein	120-134	5.7	106.5	1	89.8
SPTIPFFDPQIPKLTD	P05814	Beta-casein	120-135	6.3	142.4	1	169.9
SPTIPFFDPQIPKLTDLEN	P05814	Beta-casein	120-138	5.7	86.7	1	111.1
PTIPFFDPQIPKL	P05814	Beta-casein	121-133	5.6	55.9	1	96.3
PTIPFFDPQIPKLTD	P05814	Beta-casein	121-135	6.0	53.4	4	122.7
FDPQIPKLTDLENL	P05814	Beta-casein	126-139	5.7	106.0	2	112.1
DPQIPKLTDLENL	P05814	Beta-casein	127-139	5.9	109.5	1	107.8
DPQIPKLTDLENLH	P05814	Beta-casein	127-140	5.8	117.7	1	132.9
DPQIPKLTDLENLHLP	P05814	Beta-casein	127-142	5.9	105.7	1	132.4
DPQIPKLTDLENLHLPLP	P05814	Beta-casein	127-144	6.1	111.3	1	82.0
TDLENLHLP	P05814	Beta-casein	134-142	5.8	73.9	1	226.7
TDLENLHLPLP	P05814	Beta-casein	134-144	5.3	147.3	1	147.2
DLENLHLP	P05814	Beta-casein	135-142	5.9	89.0	1	124.9
LENLHLPLPLLQ	P05814	Beta-casein	136-147	5.1	109.1	10	65.3
ENLHLPLPLL	P05814	Beta-casein	137-146	5.3	114.5	1	87.0

Supplemental Table S 2: List with all identified peptides, including their precursor protein, sequence range, mean log10 LFQ intensity, inter-individual variation (CV), WGCNA cluster, and identification score. *(continued)*

Sequence	Uniprot ID	Leading protein name	Sequence positions	Mean log10 LFQ intensity	CV (%)	WGCNA cluster	Identification score
ENLHLPLPLLQ	P05814	Beta-casein	137-147	5.3	81.8	1	95.1
LLQPLMQQVPQPIP	P05814	Beta-casein	145-158	5.4	98.3	1	111.7
LLQPLMQQVPQPIPQT	P05814	Beta-casein	145-160	7.2	93.8	1	161.9
LLQPLMQQVPQPIPQTL	P05814	Beta-casein	145-161	6.0	224.8	2	186.9
LLQPLMQQVPQPIPQTLALPPQP	P05814	Beta-casein	145-167	6.2	193.8	1	89.5
LQPLMQQVPQPIPQT	P05814	Beta-casein	146-160	5.7	83.1	1	123.8
QPLMQQVPQPIPQT	P05814	Beta-casein	147-160	6.5	132.9	1	120.1
QPLMQQVPQPIPQTL	P05814	Beta-casein	147-161	5.5	75.9	1	86.0
QPLMQQVPQPIPQTLALPPQP	P05814	Beta-casein	147-167	5.8	128.6	1	89.0
PLMQQVPQPIPQTL	P05814	Beta-casein	148-161	5.8	90.2	4	83.0
LMQQVPQPIPQT	P05814	Beta-casein	149-160	6.5	132.5	1	109.0
LMQQVPQPIPQTLALPPQP	P05814	Beta-casein	149-167	5.8	114.2	1	71.7
MQQVPQPIPQT	P05814	Beta-casein	150-160	5.9	100.8	1	58.7
QQVPQPIPQTL	P05814	Beta-casein	151-161	6.2	60.7	4	159.1
QQVPQPIPQTLALPPQPLWSVPQP	P05814	Beta-casein	151-174	5.7	110.6	1	47.8
QVPQPIPQTL	P05814	Beta-casein	152-161	5.9	61.8	4	91.3
VPQPIPQTLALPPQP	P05814	Beta-casein	153-167	6.0	156.8	1	68.1
RETIESLSSSEE	P05814	Beta-casein	16-27	5.9	95.2	7	195.5
RETIESLSSSEESITEY	P05814	Beta-casein	16-32	6.7	65.7	7	213.8
RETIESLSSSEESITEYK	P05814	Beta-casein	16-33	7.9	76.8	7	410.6
RETIESLSSSEESITEYKQ	P05814	Beta-casein	16-34	6.0	114.5	3	116.3
RETIESLSSSEESITEYKQK	P05814	Beta-casein	16-35	6.8	87.3	7	270.5
RETIESLSSSEESITEYKQKVE	P05814	Beta-casein	16-37	6.7	93.9	7	168.7
RETIESLSSSEESITEYKQKVEK	P05814	Beta-casein	16-38	6.9	117.9	7	239.3
LALPPQPLWSVPQP	P05814	Beta-casein	161-174	5.6	125.0	1	116.8
LALPPQPLWSVPQPK	P05814	Beta-casein	161-175	5.5	142.8	6	100.7
ALPPQPLWSVPQP	P05814	Beta-casein	162-174	6.0	59.8	6	91.6
ETIESLSSSEE	P05814	Beta-casein	17-27	5.4	60.9	7	84.5
ETIESLSSSEESITE	P05814	Beta-casein	17-31	7.0	105.7	11	189.7
ETIESLSSSEESITEY	P05814	Beta-casein	17-32	6.8	55.3	7	267.6
ETIESLSSSEESITEYK	P05814	Beta-casein	17-33	8.3	55.6	7	384.8
ETIESLSSSEESITEYKQ	P05814	Beta-casein	17-34	6.4	125.4	7	279.6
ETIESLSSSEESITEYKQK	P05814	Beta-casein	17-35	7.1	83.3	7	169.8

Supplemental Table S 2: List with all identified peptides, including their precursor protein, sequence range, mean log10 LFQ intensity, inter-individual variation (CV), WGCNA cluster, and identification score. *(continued)*

Sequence	Uniprot ID	Leading protein name	Sequence positions	Mean log10 LFQ intensity	CV (%)	WGCNA cluster	Identification score
ETIESLSSSEESITEYKQKV	P05814	Beta-casein	17-36	5.9	97.5	2	45.3
ETIESLSSSEESITEYKQKVE	P05814	Beta-casein	17-37	6.6	159.7	1	197.7
ETIESLSSSEESITEYKQKVEK	P05814	Beta-casein	17-38	7.5	112.1	1	179.6
SVPQPKVLPIPQQVVPYP	P05814	Beta-casein	170-187	6.1	144.0	1	104.9
SVPQPKVLPIPQQVVPYPQ	P05814	Beta-casein	170-188	6.4	290.6	1	96.7
SVPQPKVLPIPQQVVPYPQR	P05814	Beta-casein	170-189	6.9	461.9	1	107.4
VPQPKVLPIPQQ	P05814	Beta-casein	171-182	6.0	154.2	4	93.6
VPQPKVLPIPQQV	P05814	Beta-casein	171-183	6.0	51.9	2	132.0
VPQPKVLPIPQQVVP	P05814	Beta-casein	171-185	6.5	80.6	1	85.4
VPQPKVLPIPQQVVPYP	P05814	Beta-casein	171-187	6.8	96.4	1	128.0
QPKVLPIPQQVVP	P05814	Beta-casein	173-185	5.7	69.7	1	59.4
QPKVLPIPQQVVPYP	P05814	Beta-casein	173-187	5.9	115.8	1	131.4
VLPIPQQV	P05814	Beta-casein	176-183	6.2	97.7	6	130.0
VLPIPQQVVP	P05814	Beta-casein	176-185	6.5	68.3	1	89.6
VLPIPQQVVPYP	P05814	Beta-casein	176-187	6.9	85.3	6	133.6
VLPIPQQVVPYPQ	P05814	Beta-casein	176-188	5.7	175.0	6	131.0
VLPIPQQVVPYPQR	P05814	Beta-casein	176-189	7.2	229.2	6	175.7
VLPIPQQVVPYPQRAVP	P05814	Beta-casein	176-192	5.9	71.9	1	79.7
VLPIPQQVVPYPQRAVPV	P05814	Beta-casein	176-193	5.9	179.5	2	100.7
VLPIPQQVVPYPQRAVPVQ	P05814	Beta-casein	176-194	6.6	133.1	1	108.3
VLPIPQQVVPYPQRAVPVQA	P05814	Beta-casein	176-195	7.5	72.1	1	168.4
VLPIPQQVVPYPQRAVPVQAL	P05814	Beta-casein	176-196	6.6	68.3	1	153.4
LPIPQQVVPYP	P05814	Beta-casein	177-187	6.1	107.7	1	89.3
LPIPQQVVPYPQRA	P05814	Beta-casein	177-190	6.3	75.7	1	145.9
LPIPQQVVPYPQRAV	P05814	Beta-casein	177-191	5.6	110.9	1	81.3
LPIPQQVVPYPQRAVP	P05814	Beta-casein	177-192	6.6	64.6	1	138.2
LPIPQQVVPYPQRAVPV	P05814	Beta-casein	177-193	5.7	111.8	1	58.7
LPIPQQVVPYPQRAVPVQ	P05814	Beta-casein	177-194	6.8	77.9	1	122.4
LPIPQQVVPYPQRAVPVQA	P05814	Beta-casein	177-195	7.4	88.9	1	162.7
PIPQQVVPYPQRAVPVQ	P05814	Beta-casein	178-194	6.0	74.1	2	84.7
PIPQQVVPYPQRAVPVQA	P05814	Beta-casein	178-195	6.2	93.0	2	115.8
IPQQVVPYPQRAVPVQA	P05814	Beta-casein	179-195	6.9	112.2	1	122.5

Supplemental Table S 2: List with all identified peptides, including their precursor protein, sequence range, mean log10 LFQ intensity, inter-individual variation (CV), WGCNA cluster, and identification score. *(continued)*

Sequence	Uniprot ID	Leading protein name	Sequence positions	Mean log10 LFQ intensity	CV (%)	WGCNA cluster	Identification score
TIESLSSSEESITEY	P05814	Beta-casein	18-32	6.1	68.6	7	110.4
TIESLSSSEESITEYK	P05814	Beta-casein	18-33	7.6	73.4	7	347.7
TIESLSSSEESITEYKQK	P05814	Beta-casein	18-35	5.9	82.4	7	52.5
TIESLSSSEESITEYKQKVEK	P05814	Beta-casein	18-38	6.2	118.8	7	59.5
VVPYPQRAVPVQ	P05814	Beta-casein	183-194	6.5	128.4	4	107.2
VPYPQRAVPVQ	P05814	Beta-casein	184-194	5.8	75.1	1	98.4
VPYPQRAVPVQA	P05814	Beta-casein	184-195	6.3	57.4	2	130.8
IESLSSSEESITEYK	P05814	Beta-casein	19-33	7.2	83.8	7	386.6
NQELLLNPTHQIYP	P05814	Beta-casein	199-212	5.9	143.6	2	47.3
NQELLLNPTHQIYPVT	P05814	Beta-casein	199-214	6.5	71.9	4	108.4
NQELLLNPTHQIYPVTQ	P05814	Beta-casein	199-215	6.1	58.2	4	116.0
NQELLLNPTHQIYPVTQPLAPV	P05814	Beta-casein	199-220	5.7	101.4	1	120.1
NQELLLNPTHQIYPVTQPLAPVH	P05814	Beta-casein	199-221	6.1	95.0	1	53.2
ESLSSSEESITE	P05814	Beta-casein	20-31	6.3	77.7	7	174.0
ESLSSSEESITEYK	P05814	Beta-casein	20-33	7.0	88.2	7	278.5
ESLSSSEESITEYKQKVEK	P05814	Beta-casein	20-38	6.2	155.8	1	59.0
ELLLNPTHQIYP	P05814	Beta-casein	201-212	5.6	123.8	1	55.6
ELLLNPTHQIYPVTQ	P05814	Beta-casein	201-215	5.8	68.2	1	70.5
ELLLNPTHQIYPVTQPLAPVHNPIS	P05814	Beta-casein	201-225	5.9	105.6	1	118.2
LLLNPTHQIYPVT	P05814	Beta-casein	202-214	6.3	52.5	4	73.1
LLLNPTHQIYPVTQ	P05814	Beta-casein	202-215	5.8	59.6	4	82.4
LLLNPTHQIYPVTQPLAPVHNPIS	P05814	Beta-casein	202-225	6.1	75.5	1	117.6
LLLNPTHQIYPVTQPLAPVHNPISV	P05814	Beta-casein	202-226	7.8	67.3	4	228.8
LLNPTHQIYPVTQ	P05814	Beta-casein	203-215	5.9	34.0	1	70.0
LLNPTHQIYPVTQPLAPVHNPIS	P05814	Beta-casein	203-225	6.5	103.7	2	129.3
LLNPTHQIYPVTQPLAPVHNPISV	P05814	Beta-casein	203-226	8.2	85.2	2	316.4
LNPTHQIYPVT	P05814	Beta-casein	204-214	5.5	44.4	5	78.9
LNPTHQIYPVTQ	P05814	Beta-casein	204-215	5.7	44.8	2	71.0
LNPTHQIYPVTQPLAPVHNPISV	P05814	Beta-casein	204-226	7.6	88.0	2	263.6
NPTHQIYPVTQ	P05814	Beta-casein	205-215	6.3	54.4	1	103.7
NPTHQIYPVTQPLAPVH	P05814	Beta-casein	205-221	6.5	166.2	1	228.1
NPTHQIYPVTQPLAPVHNP	P05814	Beta-casein	205-223	6.2	159.4	6	80.0
NPTHQIYPVTQPLAPVHNPIS	P05814	Beta-casein	205-225	7.0	136.6	10	190.1

Supplemental Table S 2: List with all identified peptides, including their precursor protein, sequence range, mean log10 LFQ intensity, inter-individual variation (CV), WGCNA cluster, and identification score. *(continued)*

Sequence	Uniprot ID	Leading protein name	Sequence positions	Mean log10 LFQ intensity	CV (%)	WGCNA cluster	Identification score
NPTHQIYPVTQPLAPVHNPISV	P05814	Beta-casein	205-226	7.8	64.0	1	281.1
PTHQIYPVTQPLAPVHNPIS	P05814	Beta-casein	206-225	6.0	106.3	1	113.5
PTHQIYPVTQPLAPVHNPISV	P05814	Beta-casein	206-226	7.5	53.9	5	214.2
THQIYPVTQPLAPVHNPISV	P05814	Beta-casein	207-226	6.8	61.7	9	138.1
YQEPVLGPVRGPFPIIV	P02666	Beta-casein	208-224	5.7	107.8	8	127.4
HQIYPVTQPLAPVHNPISV	P05814	Beta-casein	208-226	6.8	143.4	4	244.7
QIYPVTQPLAPVHNPISV	P05814	Beta-casein	209-226	7.3	178.9	4	258.0
SLSSSEESITE	P05814	Beta-casein	21-31	6.3	72.5	7	127.6
SLSSSEESITEY	P05814	Beta-casein	21-32	6.0	58.7	7	105.8
SLSSSEESITEYK	P05814	Beta-casein	21-33	7.2	59.6	7	250.7
SLSSSEESITEYKQKV	P05814	Beta-casein	21-36	6.3	117.9	7	147.2
SLSSSEESITEYKQKVE	P05814	Beta-casein	21-37	5.7	104.7	1	45.7
SLSSSEESITEYKQKVEK	P05814	Beta-casein	21-38	6.7	89.0	7	101.0
IYPVTQPLAPVHNPISV	P05814	Beta-casein	210-226	6.5	193.3	8	192.9
YPVTQPLAPVH	P05814	Beta-casein	211-221	5.6	173.2	1	176.8
YPVTQPLAPVHNPISV	P05814	Beta-casein	211-226	7.4	64.2	1	235.3
PVTQPLAPVH	P05814	Beta-casein	212-221	5.3	122.3	5	172.2
PVTQPLAPVHNPISV	P05814	Beta-casein	212-226	7.7	81.0	2	254.2
VTQPLAPVHNPISV	P05814	Beta-casein	213-226	7.2	159.4	2	162.2
TQPLAPVHNPI	P05814	Beta-casein	214-224	6.0	130.1	2	68.8
TQPLAPVHNPISV	P05814	Beta-casein	214-226	8.4	97.1	2	156.0
QPLAPVHNPISV	P05814	Beta-casein	215-226	8.4	67.1	2	123.6
PLAPVHNPISV	P05814	Beta-casein	216-226	8.3	45.2	4	193.1
LAPVHNPISV	P05814	Beta-casein	217-226	6.8	279.6	3	97.6
LSSSEESITEYK	P05814	Beta-casein	22-33	7.0	86.8	7	263.1
LSSSEESITEYKQKV	P05814	Beta-casein	22-36	5.8	78.0	7	57.1
LSSSEESITEYKQKVEK	P05814	Beta-casein	22-38	6.1	97.7	7	60.9
SSSEESITEYK	P05814	Beta-casein	23-33	7.2	66.0	7	255.1
SSSEESITEYKQK	P05814	Beta-casein	23-35	5.2	102.6	1	51.0
SSSEESITEYKQKV	P05814	Beta-casein	23-36	6.3	119.8	6	210.3
SSSEESITEYKQKVE	P05814	Beta-casein	23-37	5.7	86.3	7	79.6
SSSEESITEYKQKVEK	P05814	Beta-casein	23-38	6.0	119.3	6	86.6

Supplemental Table S 2: List with all identified peptides, including their precursor protein, sequence range, mean log10 LFQ intensity, inter-individual variation (CV), WGCNA cluster, and identification score. *(continued)*

Sequence	Uniprot ID	Leading protein name	Sequence positions	Mean log10 LFQ intensity	CV (%)	WGCNA cluster	Identification score
SSEESITEYK	P05814	Beta-casein	24-33	6.8	57.7	7	258.5
SEESITEYK	P05814	Beta-casein	25-33	7.0	58.0	7	218.8
SEESITEYKQKV	P05814	Beta-casein	25-36	6.4	90.7	7	222.3
EESITEYKQKV	P05814	Beta-casein	26-36	6.7	117.8	7	250.1
EESITEYKQKVEK	P05814	Beta-casein	26-38	5.4	85.9	6	53.3
ESITEYKQKVEK	P05814	Beta-casein	27-38	5.9	66.8	3	83.6
SITEYKQKVEK	P05814	Beta-casein	28-38	6.0	110.8	3	86.0
TEYKQKVE	P05814	Beta-casein	30-37	6.2	82.0	4	173.7
TEYKQKVEKV	P05814	Beta-casein	30-39	6.8	63.1	4	165.0
TEYKQKVEKVKHED	P05814	Beta-casein	30-43	5.9	61.1	4	204.8
TEYKQKVEKVKHEDQQQ	P05814	Beta-casein	30-46	5.8	86.8	4	126.8
TEYKQKVEKVKHEDQQQGEDE	P05814	Beta-casein	30-50	5.3	125.7	10	98.1
KQKVEKVKHEDQQQG	P05814	Beta-casein	33-47	5.2	161.1	2	84.5
QKVEKVKHEDQQQG	P05814	Beta-casein	34-47	6.2	80.5	3	92.9
QKVEKVKHEDQQQGEDEHQD	P05814	Beta-casein	34-53	7.0	93.9	3	265.5
QKVEKVKHEDQQQGEDEHQDK	P05814	Beta-casein	34-54	7.1	118.8	6	384.1
KVEKVKHEDQQQGEDEHQDK	P05814	Beta-casein	35-54	6.4	123.8	6	360.0
VEKVKHEDQQQGEDEHQD	P05814	Beta-casein	36-53	6.6	78.6	6	311.0
VEKVKHEDQQQGEDEHQDK	P05814	Beta-casein	36-54	7.0	142.4	6	386.5
EKVKHEDQQQGEDEHQD	P05814	Beta-casein	37-53	6.3	150.7	1	341.7
EKVKHEDQQQGEDEHQDK	P05814	Beta-casein	37-54	6.4	179.4	6	317.5
KVKHEDQQQGEDEHQDK	P05814	Beta-casein	38-54	5.7	405.8	2	280.2
VKHEDQQQGEDEHQ	P05814	Beta-casein	39-52	5.5	168.5	3	208.8
VKHEDQQQGEDEHQD	P05814	Beta-casein	39-53	7.5	134.0	3	397.5
VKHEDQQQGEDEHQDK	P05814	Beta-casein	39-54	7.8	152.1	3	411.3
VKHEDQQQGEDEHQDKIYP	P05814	Beta-casein	39-57	6.1	74.3	3	74.1
VKHEDQQQGEDEHQDKIYPS	P05814	Beta-casein	39-58	6.4	82.2	6	198.2
VKHEDQQQGEDEHQDKIYPSFQPQP	P05814	Beta-casein	39-63	6.9	110.7	1	169.4
HEDQQQGEDEHQD	P05814	Beta-casein	41-53	6.3	82.9	3	370.2
HEDQQQGEDEHQDK	P05814	Beta-casein	41-54	6.7	84.2	3	353.8
HEDQQQGEDEHQDKIYP	P05814	Beta-casein	41-57	6.3	87.2	2	125.5
HEDQQQGEDEHQDKIYPS	P05814	Beta-casein	41-58	6.1	64.3	6	175.5
HEDQQQGEDEHQDKIYPSFQPQP	P05814	Beta-casein	41-63	6.0	75.6	1	73.3

Supplemental Table S 2: List with all identified peptides, including their precursor protein, sequence range, mean log10 LFQ intensity, inter-individual variation (CV), WGCNA cluster, and identification score. *(continued)*

Sequence	Uniprot ID	Leading protein name	Sequence positions	Mean log10 LFQ intensity	CV (%)	WGCNA cluster	Identification score
EDQQQGEDEHQD	P05814	Beta-casein	42-53	5.3	81.5	3	120.1
EDQQQGEDEHQDK	P05814	Beta-casein	42-54	5.3	127.4	3	62.1
EDQQQGEDEHQDKIYP	P05814	Beta-casein	42-57	5.7	58.1	2	45.9
EDQQQGEDEHQDKIYPS	P05814	Beta-casein	42-58	5.6	92.0	4	94.5
DQQQGEDEHQDKIYP	P05814	Beta-casein	43-57	6.2	49.2	2	125.3
DQQQGEDEHQDKIYPS	P05814	Beta-casein	43-58	6.4	65.3	6	202.6
GEDEHQDK	P05814	Beta-casein	47-54	5.2	77.3	7	241.5
GEDEHQDKIYPS	P05814	Beta-casein	47-58	5.4	108.3	2	64.6
FQSEEQQQTEDELQDK	P02666	Beta-casein	48-63	5.4	43.5	6	111.4
VYPFPGPIPN	P02666	Beta-casein	74-83	6.0	67.9	8	108.2
GFLPQNILPLAQPA	P05814	Beta-casein	75-88	5.9	197.7	2	129.4
QNILPLAQPAVVLPVPQPEIMEVPK	P05814	Beta-casein	79-103	5.6	93.3	1	83.0
ILPLAQPAVVLPVPQPEIMEVPK	P05814	Beta-casein	81-103	6.3	326.2	1	104.0
PLAQPAVVLPVPQPEI	P05814	Beta-casein	83-98	6.2	74.9	4	110.9
LAQPAVVLPVPQPEIMEVPK	P05814	Beta-casein	84-103	6.5	107.2	6	93.8
LAQPAVVLPVPQPEIMEVPKA	P05814	Beta-casein	84-104	5.8	167.4	1	114.7
LAQPAVVLPVPQPEIMEVPKAKDT	P05814	Beta-casein	84-107	5.7	74.8	6	85.3
AQPAVVLPVPQPEIMEVPK	P05814	Beta-casein	85-103	6.6	73.0	6	138.9
AQPAVVLPVPQPEIMEVPKA	P05814	Beta-casein	85-104	6.0	101.8	1	132.1
AQPAVVLPVPQPEIMEVPKAKDT	P05814	Beta-casein	85-107	6.0	80.0	1	63.2
AQPAVVLPVPQPEIMEVPKAKDTVY	P05814	Beta-casein	85-109	6.0	149.2	1	52.5
QPAVVLPVPQPEIMEVP	P05814	Beta-casein	86-102	5.5	158.7	1	75.7
QPAVVLPVPQPEIMEVPK	P05814	Beta-casein	86-103	7.1	102.1	1	155.1
QPAVVLPVPQPEIMEVPKA	P05814	Beta-casein	86-104	6.4	127.3	1	156.6
QPAVVLPVPQPEIMEVPKAKDTVYT	P05814	Beta-casein	86-110	6.1	105.4	3	80.2
QPAVVLPVPQPEI	P05814	Beta-casein	86-98	5.6	73.8	1	74.2
PAVVLPVPQPEI	P05814	Beta-casein	87-98	5.8	62.0	4	90.6
AVVLPVPQPEIMEVPKAKDTVYT	P05814	Beta-casein	88-110	6.4	78.8	1	55.8
VVLPVPQPEIMEVPK	P05814	Beta-casein	89-103	7.0	143.9	2	112.4
VVLPVPQPEIMEVPKA	P05814	Beta-casein	89-104	6.4	159.9	2	151.9
VVLPVPQPEIMEVPKAKDTVYT	P05814	Beta-casein	89-110	7.3	137.6	10	87.6
VLPVPQPEIMEVPK	P05814	Beta-casein	90-103	6.3	139.6	2	101.9

Supplemental Table S 2: List with all identified peptides, including their precursor protein, sequence range, mean log10 LFQ intensity, inter-individual variation (CV), WGCNA cluster, and identification score. *(continued)*

Sequence	Uniprot ID	Leading protein name	Sequence positions	Mean log10 LFQ intensity	CV (%)	WGCNA cluster	Identification score
LPVPQPEIME	P05814	Beta-casein	91-100	5.7	104.9	1	79.9
LPVPQPEIMEVP	P05814	Beta-casein	91-102	5.8	93.0	3	54.6
LPVPQPEIMEVPK	P05814	Beta-casein	91-103	7.5	74.6	1	142.1
LPVPQPEIMEVPKA	P05814	Beta-casein	91-104	6.7	92.6	1	137.2
LPVPQPEIMEVPKAKDT	P05814	Beta-casein	91-107	6.9	69.9	1	179.5
LPVPQPEIMEVPKAKDTV	P05814	Beta-casein	91-108	6.4	71.1	1	137.4
LPVPQPEIMEVPKAKDTVYT	P05814	Beta-casein	91-110	7.1	75.1	1	120.3
VPQPEIMEVPK	P05814	Beta-casein	93-103	6.4	109.5	1	143.5
TQTPVVVPPFLQPEV	P02666	Beta-casein	93-107	5.9	67.8	8	70.0
VPQPEIMEVPKAKDT	P05814	Beta-casein	93-107	6.3	91.4	1	130.2
VPQPEIMEVPKAKDTVYT	P05814	Beta-casein	93-110	6.6	82.7	1	147.2
QPEIMEVPKAKDT	P05814	Beta-casein	95-107	5.5	106.1	1	50.6
TPVVVPPFLQPEV	P02666	Beta-casein	95-107	5.6	64.5	8	70.1
QPEIMEVPKAKDTVYT	P05814	Beta-casein	95-110	6.4	73.0	1	45.7
EIMEVPKAKDTVYT	P05814	Beta-casein	97-110	6.7	69.3	1	149.3
EIMEVPKAKDTVYTK	P05814	Beta-casein	97-111	6.1	106.9	6	80.5
IMEVPKAKDTVYT	P05814	Beta-casein	98-110	5.7	91.0	1	75.8
MEVPKAKDTVYT	P05814	Beta-casein	99-110	6.2	54.9	3	99.6
MEVPKAKDTVYTK	P05814	Beta-casein	99-111	5.4	79.7	3	70.6
MEVPKAKDTVYTKG	P05814	Beta-casein	99-112	5.6	104.9	4	96.7
AKLGAVYTEGGFVEGVNK	P19835	Bile salt-activated lipase	21-38	6.3	88.4	3	229.6
AKLGAVYTEGGFVEGVNKK	P19835	Bile salt-activated lipase	21-39	6.5	100.4	3	145.1
KLGAVYTEGGFVEGVNK	P19835	Bile salt-activated lipase	22-38	6.3	62.7	3	205.3
KLGAVYTEGGFVEGVNKK	P19835	Bile salt-activated lipase	22-39	6.3	69.2	3	214.5
GAVYTEGGFVEGVNK	P19835	Bile salt-activated lipase	24-38	6.2	56.3	3	210.9
GAVYTEGGFVEGVNKK	P19835	Bile salt-activated lipase	24-39	6.2	105.4	3	78.4

Supplemental Table S 2: List with all identified peptides, including their precursor protein, sequence range, mean log10 LFQ intensity, inter-individual variation (CV), WGCNA cluster, and identification score. *(continued)*

Sequence	Uniprot ID	Leading protein name	Sequence positions	Mean log10 LFQ intensity	CV (%)	WGCNA cluster	Identification score
TEGGFVEGVNK	P19835	Bile salt-activated lipase	28-38	6.1	57.1	3	207.8
TEGGFVEGVNKK	P19835	Bile salt-activated lipase	28-39	6.1	73.8	3	134.6
EGGFVEGVNK	P19835	Bile salt-activated lipase	29-38	6.6	69.4	3	114.5
GNKKVTEEDFYK	P19835	Bile salt-activated lipase	355-366	5.7	107.1	3	110.9
TTENSGYLEITK	P19835	Bile salt-activated lipase	512-523	5.5	100.6	2	131.8
GIPFAAPTKALENPQPHPGWQGTL	P19835	Bile salt-activated lipase	52-75	6.6	61.5	6	120.5
GIPFAAPTKALENPQPHPGWQGTLK	P19835	Bile salt-activated lipase	52-76	6.2	119.1	3	60.7
APTKALENPQPHPGWQGTL	P19835	Bile salt-activated lipase	57-75	5.8	54.7	6	63.3
ALENPQPHPGWQGTLK	P19835	Bile salt-activated lipase	61-76	5.4	108.8	3	67.4
IPASSLPRLTPWI	Q13410	Butyrophilin subfamily 1 member A1	235-247	5.3	80.0	1	152.1
IPASSLPRLTPWIV	Q13410	Butyrophilin subfamily 1 member A1	235-248	5.5	68.6	1	129.9
IPASSLPRLTPWIVA	Q13410	Butyrophilin subfamily 1 member A1	235-249	5.8	91.3	7	122.7
IPASSLPRLTPWIVAVA	Q13410	Butyrophilin subfamily 1 member A1	235-251	5.4	78.9	1	116.5
APFDVIGPPEPI	Q13410	Butyrophilin subfamily 1 member A1	27-38	5.6	119.4	2	70.0

Supplemental Table S 2: List with all identified peptides, including their precursor protein, sequence range, mean log10 LFQ intensity, inter-individual variation (CV), WGCNA cluster, and identification score. *(continued)*

Sequence	Uniprot ID	Leading protein name	Sequence positions	Mean log10 LFQ intensity	CV (%)	WGCNA cluster	Identification score
APFDVIGPPEPILA	Q13410	Butyrophilin subfamily 1 member A1	27-40	5.4	80.8	4	101.1
YEDSKSVRLE	Q13410	Butyrophilin subfamily 1 member A1	319-328	5.3	92.0	3	78.7
DGPERVTVIANAQDLS	Q13410	Butyrophilin subfamily 1 member A1	477-492	6.3	65.9	4	124.3
KEIPLSPMGED	Q13410	Butyrophilin subfamily 1 member A1	493-503	5.6	63.8	9	91.9
KEIPLSPMGEDS	Q13410	Butyrophilin subfamily 1 member A1	493-504	5.9	113.4	10	105.9
KEIPLSPMGEDSAPR	Q13410	Butyrophilin subfamily 1 member A1	493-507	6.2	78.2	10	150.4
KEIPLSPMGEDSAPRD	Q13410	Butyrophilin subfamily 1 member A1	493-508	5.8	88.6	10	86.6
KEIPLSPMGEDSAPRDADTLH	Q13410	Butyrophilin subfamily 1 member A1	493-513	7.3	73.4	10	196.6
KEIPLSPMGEDSAPRDADTLHS	Q13410	Butyrophilin subfamily 1 member A1	493-514	6.4	92.2	10	191.8
EIPLSPMGEDSAPR	Q13410	Butyrophilin subfamily 1 member A1	494-507	6.3	91.2	10	84.7
EIPLSPMGEDSAPRDADTLH	Q13410	Butyrophilin subfamily 1 member A1	494-513	6.8	82.3	10	60.6

Supplemental Table S 2: List with all identified peptides, including their precursor protein, sequence range, mean log10 LFQ intensity, inter-individual variation (CV), WGCNA cluster, and identification score. *(continued)*

Sequence	Uniprot ID	Leading protein name	Sequence positions	Mean log10 LFQ intensity	CV (%)	WGCNA cluster	Identification score
IPLSPMGEDSAPR	Q13410	Butyrophilin subfamily 1 member A1	495-507	6.7	74.2	10	215.6
IPLSPMGEDSAPRD	Q13410	Butyrophilin subfamily 1 member A1	495-508	5.9	86.5	10	99.5
IPLSPMGEDSAPRDADTLH	Q13410	Butyrophilin subfamily 1 member A1	495-513	6.9	60.7	10	170.9
PLSPMGEDSAPRD	Q13410	Butyrophilin subfamily 1 member A1	496-508	5.8	81.0	10	111.1
SPMGEDSAPR	Q13410	Butyrophilin subfamily 1 member A1	498-507	5.5	75.6	10	94.7
SPMGEDSAPRD	Q13410	Butyrophilin subfamily 1 member A1	498-508	5.8	96.9	10	146.1
SPMGEDSAPRDADTLH	Q13410	Butyrophilin subfamily 1 member A1	498-513	6.1	80.0	10	113.4
GEDSAPRDADTLH	Q13410	Butyrophilin subfamily 1 member A1	501-513	5.8	74.3	10	58.7
GEDSAPRDADTLHS	Q13410	Butyrophilin subfamily 1 member A1	501-514	5.1	73.3	10	68.8
SAPRDADTLHSKLIPTQPSQGAP	Q13410	Butyrophilin subfamily 1 member A1	504-526	6.1	64.5	5	60.7
APRDADTLH	Q13410	Butyrophilin subfamily 1 member A1	505-513	6.3	103.8	7	98.3

Supplemental Table S 2: List with all identified peptides, including their precursor protein, sequence range, mean log10 LFQ intensity, inter-individual variation (CV), WGCNA cluster, and identification score. *(continued)*

Sequence	Uniprot ID	Leading protein name	Sequence positions	Mean log10 LFQ intensity	CV (%)	WGCNA cluster	Identification score
APRDADTLHSKLIP	Q13410	Butyrophilin subfamily 1 member A1	505-518	5.9	69.9	5	71.6
APRDADTLHSKLIPT	Q13410	Butyrophilin subfamily 1 member A1	505-519	5.6	65.2	6	55.9
APRDADTLHSKLIPTQPSQGAP	Q13410	Butyrophilin subfamily 1 member A1	505-526	6.7	66.6	6	67.3
DADTLHSKLIPTQPSQGAP	Q13410	Butyrophilin subfamily 1 member A1	508-526	6.9	54.8	4	77.9
ADTLHSKLIPTQPSQGAP	Q13410	Butyrophilin subfamily 1 member A1	509-526	6.6	108.7	5	161.8
DTLHSKLI	Q13410	Butyrophilin subfamily 1 member A1	510-517	5.4	68.2	8	122.0
DTLHSKLIPTQPSQGAP	Q13410	Butyrophilin subfamily 1 member A1	510-526	5.5	53.8	10	54.0
HSKLIPTQPSQGAP	Q13410	Butyrophilin subfamily 1 member A1	513-526	5.5	101.9	11	71.0
SKLIPTQPSQG	Q13410	Butyrophilin subfamily 1 member A1	514-524	6.6	75.0	5	130.0
SKLIPTQPSQGAP	Q13410	Butyrophilin subfamily 1 member A1	514-526	6.9	64.6	4	126.0
KLIPTQPSQG	Q13410	Butyrophilin subfamily 1 member A1	515-524	6.0	65.9	5	66.3

Supplemental Table S 2: List with all identified peptides, including their precursor protein, sequence range, mean log10 LFQ intensity, inter-individual variation (CV), WGCNA cluster, and identification score. *(continued)*

Sequence	Uniprot ID	Leading protein name	Sequence positions	Mean log10 LFQ intensity	CV (%)	WGCNA cluster	Identification score
KLIPTQPSQGAP	Q13410	Butyrophilin subfamily 1 member A1	515-526	6.2	150.9	2	61.3
KVSPAVLVHRDG	Q13410	Butyrophilin subfamily 1 member A1	69-80	5.6	110.5	4	68.0
SPAVLVHRDGREQE	Q13410	Butyrophilin subfamily 1 member A1	71-84	5.9	38.5	9	173.7
SPAVLVHRDGREQEAEQMPEY	Q13410	Butyrophilin subfamily 1 member A1	71-91	6.6	107.9	1	167.4
DGREQEAEQMPE	Q13410	Butyrophilin subfamily 1 member A1	79-90	6.1	47.4	11	112.8
DGREQEAEQMPEY	Q13410	Butyrophilin subfamily 1 member A1	79-91	7.2	49.3	9	172.1
DGREQEAEQMPEYR	Q13410	Butyrophilin subfamily 1 member A1	79-92	7.5	61.2	11	329.7
DGREQEAEQMPEYRG	Q13410	Butyrophilin subfamily 1 member A1	79-93	6.8	101.8	9	203.9
DGREQEAEQMPEYRGR	Q13410	Butyrophilin subfamily 1 member A1	79-94	7.2	67.0	11	180.6
GREQEAEQMPEY	Q13410	Butyrophilin subfamily 1 member A1	80-91	6.0	87.7	9	139.9
GREQEAEQMPEYR	Q13410	Butyrophilin subfamily 1 member A1	80-92	6.1	86.9	11	115.1

Supplemental Table S 2: List with all identified peptides, including their precursor protein, sequence range, mean log10 LFQ intensity, inter-individual variation (CV), WGCNA cluster, and identification score. *(continued)*

Sequence	Uniprot ID	Leading protein name	Sequence positions	Mean log10 LFQ intensity	CV (%)	WGCNA cluster	Identification score
GREQEAEQMPEYRGR	Q13410	Butyrophilin subfamily 1 member A1	80-94	5.6	79.7	11	147.1
GRATLVQDGIA	Q13410	Butyrophilin subfamily 1 member A1	93-103	6.3	85.2	5	73.8
GRATLVQDGIAK	Q13410	Butyrophilin subfamily 1 member A1	93-104	6.5	68.3	11	132.3
GRATLVQDGIAKGRVA	Q13410	Butyrophilin subfamily 1 member A1	93-108	6.1	63.9	5	115.3
GRATLVQDGIAKGRVAL	Q13410	Butyrophilin subfamily 1 member A1	93-109	5.8	52.1	10	78.2
TLVQDGIAKGRVA	Q13410	Butyrophilin subfamily 1 member A1	96-108	5.8	112.8	5	73.8
APLSFIPR	Q6WN34	Chordin-like protein 2	221-228	5.8	91.2	9	127.8
HFRPKGAGSTTV	Q6WN34	Chordin-like protein 2	229-240	5.7	84.0	5	60.4
HFRPKGAGSTTVK	Q6WN34	Chordin-like protein 2	229-241	5.6	94.8	11	87.0
RPKGAGSTTVKIV	Q6WN34	Chordin-like protein 2	231-243	5.6	91.0	8	82.4
RPKGAGSTTVKIVL	Q6WN34	Chordin-like protein 2	231-244	5.9	78.8	6	75.7
GAGSTTVKIVLK	Q6WN34	Chordin-like protein 2	234-245	5.6	64.5	3	94.6
APGRVLVHTSVSPSPDNL	Q6WN34	Chordin-like protein 2	334-351	5.6	58.0	7	74.8
ELKVTASPDKVTKT	Q6WN34	Chordin-like protein 2	416-429	5.8	76.8	2	65.5

Supplemental Table S 2: List with all identified peptides, including their precursor protein, sequence range, mean log10 LFQ intensity, inter-individual variation (CV), WGCNA cluster, and identification score. *(continued)*

Sequence	Uniprot ID	Leading protein name	Sequence positions	Mean log10 LFQ intensity	CV (%)	WGCNA cluster	Identification score
KVTASPDKVTKT	Q6WN34	Chordin-like protein 2	418-429	6.1	66.1	10	150.3
SLERLPSPDPGAEGHGQS	H0YCU0	Chordin-like protein 2	64-81	5.6	103.7	2	94.6
PGAEGHGQSRQSDQDITKT	H0YCU0	Chordin-like protein 2	73-91	5.6	82.8	8	132.8
QSDQDITKT	H0YCU0	Chordin-like protein 2	83-91	6.1	104.0	4	214.8
NGFKSHALQLNNRQ	P0C0L5	Complement C4-B	1337-1350	5.4	142.1	1	101.5
NGFKSHALQLNNRQI	P0C0L5	Complement C4-B	1337-1351	6.4	256.8	9	270.2
NGFKSHALQLNNRQIR	P0C0L5	Complement C4-B	1337-1352	5.4	138.0	1	56.9
DDPDAPLQPVTPLQ	P0C0L5	Complement C4-B	1429-1442	5.9	100.2	1	71.6
DDPDAPLQPVTPLQL	P0C0L5	Complement C4-B	1429-1443	5.9	90.0	1	97.2
DDPDAPLQPVTPLQLFEG	P0C0L5	Complement C4-B	1429-1446	6.1	97.4	9	125.6
DDPDAPLQPVTPLQLFEGR	P0C0L5	Complement C4-B	1429-1447	6.3	77.1	1	121.8
DDPDAPLQPVTPLQLFEGRRN	P0C0L5	Complement C4-B	1429-1449	7.2	83.2	1	122.0
PDAPLQPVTPLQLFEGRRN	P0C0L5	Complement C4-B	1431-1449	5.5	109.5	7	71.0
DSGEGDFLAEGGGVR	P02671	Fibrinogen alpha chain	21-35	7.2	65.2	11	326.4
KPVPDLVPGNF	P02671	Fibrinogen alpha chain	227-237	5.7	123.5	9	85.7
EGDFLAEGGGVR	P02671	Fibrinogen alpha chain	24-35	6.0	118.5	4	211.3
SSSYSKQFTSSTSYNRGDSTFES	P02671	Fibrinogen alpha chain	576-598	5.7	59.1	1	112.0

Supplemental Table S 2: List with all identified peptides, including their precursor protein, sequence range, mean log10 LFQ intensity, inter-individual variation (CV), WGCNA cluster, and identification score. *(continued)*

Sequence	Uniprot ID	Leading protein name	Sequence positions	Mean log10 LFQ intensity	CV (%)	WGCNA cluster	Identification score
DEAGSEADHEGTHS	P02671	Fibrinogen alpha chain	605-618	5.8	74.7	11	205.1
DEAGSEADHEGTHST	P02671	Fibrinogen alpha chain	605-619	5.4	71.7	9	60.2
DEAGSEADHEGTHSTK	P02671	Fibrinogen alpha chain	605-620	5.5	82.5	5	74.7
DEAGSEADHEGTHSTKRG	P02671	Fibrinogen alpha chain	605-622	6.1	85.3	5	117.4
DEAGSEADHEGTHSTKRGHA	P02671	Fibrinogen alpha chain	605-624	6.0	84.3	9	199.8
GPEAAKSDETAAK	P04792	Heat shock protein beta-1	193-205	5.6	87.3	4	99.8
RPNLHPSFIAIPPKKIQD	P07498	Kappa-casein	110-127	5.8	125.7	1	39.2
IAIPPKKIQDK	P07498	Kappa-casein	118-128	5.8	90.4	3	88.7
RPAIAINNPYVP	P07498	Kappa-casein	66-77	5.8	139.0	1	72.5
RPAIAINNPYVPR	P07498	Kappa-casein	66-78	5.9	122.1	2	77.6
TYYANPAVVRPHAQIP	P07498	Kappa-casein	79-94	6.3	163.4	9	65.1
TYYANPAVVRPHAQIPQ	P07498	Kappa-casein	79-95	6.3	107.4	9	122.4
TYYANPAVVRPHAQIPQR	P07498	Kappa-casein	79-96	6.6	67.2	9	141.8
YANPAVVRPHAQIPQR	P07498	Kappa-casein	81-96	5.6	109.7	9	88.1
ANPAVVRPHAQIPQ	P07498	Kappa-casein	82-95	5.6	89.9	1	49.4
NPAVVRPHAQIPQR	P07498	Kappa-casein	83-96	5.6	154.6	9	96.7
VVRPHAQIPQRQYLPNSHPPTVV	P07498	Kappa-casein	86-108	6.1	178.0	1	92.5
IPQRQYLPNSHPPTVV	P07498	Kappa-casein	93-108	5.9	119.1	1	86.0
IPQRQYLPNSHPPTVVR	P07498	Kappa-casein	93-109	6.5	164.4	9	27.5
RQYLPNSHPPTVVR	P07498	Kappa-casein	96-109	5.3	127.2	6	51.5
LPNSHPPTVVR	P07498	Kappa-casein	99-109	6.6	255.3	9	93.3
DPSKPSSNVA	P22897	Macrophage mannose receptor 1	1380-1389	5.0	143.9	2	100.0
DPSKPSSNVAG	P22897	Macrophage mannose receptor 1	1380-1390	6.2	89.8	4	101.4
DPSKPSSNVAGVVI	P22897	Macrophage mannose receptor 1	1380-1393	6.4	52.0	8	39.3

Supplemental Table S 2: List with all identified peptides, including their precursor protein, sequence range, mean log10 LFQ intensity, inter-individual variation (CV), WGCNA cluster, and identification score. *(continued)*

Sequence	Uniprot ID	Leading protein name	Sequence positions	Mean log10 LFQ intensity	CV (%)	WGCNA cluster	Identification score
DPSKPSSNVAGVVII	P22897	Macrophage mannose receptor 1	1380-1394	7.1	45.0	11	128.2
DPSKPSSNVAGVVIIV	P22897	Macrophage mannose receptor 1	1380-1395	7.3	45.6	11	122.4
DPSKPSSNVAGVVIIVI	P22897	Macrophage mannose receptor 1	1380-1396	6.7	43.6	11	106.5
KPSSNVAGVVII	P22897	Macrophage mannose receptor 1	1383-1394	5.7	77.3	10	54.4
KPSSNVAGVVIIV	P22897	Macrophage mannose receptor 1	1383-1395	5.8	67.0	10	75.3
EYPTYHTHGRYVPPS	P15941	Mucin-1	1208-1222	5.9	59.4	7	66.2
EYPTYHTHGRYVPPSS	P15941	Mucin-1	1208-1223	6.3	77.0	5	51.8
EYPTYHTHGRYVPPSSTD	P15941	Mucin-1	1208-1225	5.6	77.4	3	43.4
YPTYHTHGRYVPP	P15941	Mucin-1	1209-1221	5.5	117.9	6	74.6
YPTYHTHGRYVPPSS	P15941	Mucin-1	1209-1223	5.8	52.9	5	46.4
HTHGRYVPPSS	P15941	Mucin-1	1213-1223	5.7	93.9	5	84.7
HTHGRYVPPSSTD	P15941	Mucin-1	1213-1225	5.1	100.4	1	53.3
PPSSTDRSPYE	P15941	Mucin-1	1220-1230	5.3	65.2	5	63.3
STDRSPYEKVSAGNGGSSLS	P15941	Mucin-1	1223-1242	6.3	52.4	5	92.3
STDRSPYEKVSAGNGGSSLSY	P15941	Mucin-1	1223-1243	6.3	65.2	5	164.6
TDRSPYEKVSAGNGGSSL	P15941	Mucin-1	1224-1241	5.9	94.3	5	65.7
TDRSPYEKVSAGNGGSSLS	P15941	Mucin-1	1224-1242	6.9	51.6	5	153.2

Supplemental Table S 2: List with all identified peptides, including their precursor protein, sequence range, mean log10 LFQ intensity, inter-individual variation (CV), WGCNA cluster, and identification score. *(continued)*

Sequence	Uniprot ID	Leading protein name	Sequence positions	Mean log10 LFQ intensity	CV (%)	WGCNA cluster	Identification score
TDRSPYEKVSAGNGGSSLSY	P15941	Mucin-1	1224-1243	6.9	61.9	5	158.0
DRSPYEKVSAGNGGSSL	P15941	Mucin-1	1225-1241	5.9	79.9	5	64.5
DRSPYEKVSAGNGGSSLS	P15941	Mucin-1	1225-1242	6.6	62.0	5	116.3
DRSPYEKVSAGNGGSSLSY	P15941	Mucin-1	1225-1243	6.7	64.8	10	191.2
SPYEKVSAGNGGS	P15941	Mucin-1	1227-1239	5.6	62.0	5	152.0
SPYEKVSAGNGGSS	P15941	Mucin-1	1227-1240	6.0	55.7	5	196.4
SPYEKVSAGNGGSSL	P15941	Mucin-1	1227-1241	6.5	88.0	5	285.5
SPYEKVSAGNGGSSLS	P15941	Mucin-1	1227-1242	6.8	49.3	5	302.6
SPYEKVSAGNGGSSLSY	P15941	Mucin-1	1227-1243	6.8	46.8	5	299.6
YEKVSAGNGGSS	P15941	Mucin-1	1229-1240	4.9	108.2	7	107.1
YEKVSAGNGGSSL	P15941	Mucin-1	1229-1241	5.8	96.9	5	206.1
YEKVSAGNGGSSLS	P15941	Mucin-1	1229-1242	5.5	56.3	2	82.2
YEKVSAGNGGSSLSY	P15941	Mucin-1	1229-1243	5.5	67.1	5	121.0
EKVSAGNGGSSL	P15941	Mucin-1	1230-1241	5.7	95.7	5	150.1
EKVSAGNGGSSLS	P15941	Mucin-1	1230-1242	5.9	50.8	4	152.5
TNPAVAATSANL	P15941	Mucin-1	1244-1255	6.7	68.9	3	83.4
NPAVAATSANL	P15941	Mucin-1	1245-1255	6.9	47.3	11	220.0

Supplemental Table S 2: List with all identified peptides, including their precursor protein, sequence range, mean log10 LFQ intensity, inter-individual variation (CV), WGCNA cluster, and identification score. *(continued)*

Sequence	Uniprot ID	Leading protein name	Sequence positions	Mean log10 LFQ intensity	CV (%)	WGCNA cluster	Identification score
TYDGRGDSVVYGLR	P10451	Osteopontin	155-168	6.9	82.7	8	163.8
TYDGRGDSVVYGLRSK	P10451	Osteopontin	155-170	5.5	81.6	3	67.7
YDGRGDSVVYGLR	P10451	Osteopontin	156-168	6.1	83.8	6	112.4
DGRGDSVVYGLR	P10451	Osteopontin	157-168	6.3	118.7	8	141.1
DGRGDSVVYGLRSKSKKF	P10451	Osteopontin	157-174	5.6	99.3	2	31.8
GRGDSVVYGLR	P10451	Osteopontin	158-168	6.5	82.3	8	204.3
GRGDSVVYGLRSK	P10451	Osteopontin	158-170	5.8	70.6	3	63.7
GRGDSVVYGLRSKSKKF	P10451	Osteopontin	158-174	5.9	62.2	8	41.6
RGDSVVYGLR	P10451	Osteopontin	159-168	6.7	85.9	8	255.1
RGDSVVYGLRSK	P10451	Osteopontin	159-170	5.8	70.3	3	86.9
RGDSVVYGLRSKS	P10451	Osteopontin	159-171	5.4	95.4	8	52.5
DSVVYGLR	P10451	Osteopontin	161-168	7.0	107.8	8	174.2
DSVVYGLRSK	P10451	Osteopontin	161-170	5.7	116.8	3	91.6
DSVVYGLRSKSKK	P10451	Osteopontin	161-173	5.0	85.0	3	85.3
DSVVYGLRSKSKKF	P10451	Osteopontin	161-174	5.9	96.8	8	111.1
SVVYGLRSKSKKF	P10451	Osteopontin	162-174	5.7	97.7	8	95.4
VYGLRSKSKKF	P10451	Osteopontin	164-174	5.6	186.3	2	75.0
YGLRSKSKKF	P10451	Osteopontin	165-174	5.5	211.2	2	80.0
SKSKKFRRPD	P10451	Osteopontin	169-178	5.6	113.1	10	78.7
SKSKKFRRPDIQYPD	P10451	Osteopontin	169-183	5.4	90.6	2	63.7
SKSKKFRRPDIQYPDATD	P10451	Osteopontin	169-186	6.9	114.7	11	111.2
SKSKKFRRPDIQYPDATDEDITSH	P10451	Osteopontin	169-192	6.8	165.9	11	189.5
IPVKQADSG	P10451	Osteopontin	17-25	6.0	101.5	8	93.4
SKKFRRPDIQYPDATD	P10451	Osteopontin	171-186	6.2	59.3	7	112.7
KFRRPDIQYPDATD	P10451	Osteopontin	173-186	5.8	101.6	4	88.1
FRRPDIQYPDATD	P10451	Osteopontin	174-186	6.2	89.4	2	106.0
RRPDIQYPDATD	P10451	Osteopontin	175-186	5.6	48.9	5	140.1
RRPDIQYPDATDEDITSH	P10451	Osteopontin	175-192	5.9	150.1	2	211.1
RPDIQYPDATD	P10451	Osteopontin	176-186	6.6	66.9	2	182.7
RPDIQYPDATDED	P10451	Osteopontin	176-188	5.6	50.2	3	87.4
RPDIQYPDATDEDIT	P10451	Osteopontin	176-190	6.5	101.9	2	77.6
RPDIQYPDATDEDITSH	P10451	Osteopontin	176-192	7.1	99.4	2	278.5

Supplemental Table S 2: List with all identified peptides, including their precursor protein, sequence range, mean log10 LFQ intensity, inter-individual variation (CV), WGCNA cluster, and identification score. *(continued)*

Sequence	Uniprot ID	Leading protein name	Sequence positions	Mean log10 LFQ intensity	CV (%)	WGCNA cluster	Identification score
DIQYPDATDEDIT	P10451	Osteopontin	178-190	6.2	100.0	5	96.8
DIQYPDATDEDITSH	P10451	Osteopontin	178-192	7.0	81.6	2	164.0
DIQYPDATDEDITSHMESEELNGAY	P10451	Osteopontin	178-202	6.4	110.3	1	114.9
YPDATDEDITSH	P10451	Osteopontin	181-192	6.1	116.7	2	290.9
DATDEDITSH	P10451	Osteopontin	183-192	6.0	97.2	1	258.2
ATDEDITSH	P10451	Osteopontin	184-192	5.1	93.7	4	225.8
TDEDITSH	P10451	Osteopontin	185-192	5.5	120.8	6	130.1
EDITSHME	P10451	Osteopontin	187-194	5.7	88.3	8	126.2
EDITSHMESEELNGAYK	P10451	Osteopontin	187-203	5.7	76.3	4	87.9
NGAYKAIPVAQD	P10451	Osteopontin	199-210	5.4	60.9	3	96.9
GAYKAIPVAQDLNAPSD	P10451	Osteopontin	200-216	6.2	61.3	8	89.4
AIPVAQDLNAPS	P10451	Osteopontin	204-215	6.4	86.8	8	141.9
AIPVAQDLNAPSD	P10451	Osteopontin	204-216	6.8	59.2	3	199.1
AIPVAQDLNAPSDWD	P10451	Osteopontin	204-218	5.9	110.8	3	128.0
IPVAQDLNAPS	P10451	Osteopontin	205-215	7.0	135.5	2	216.1
PVAQDLNAPSD	P10451	Osteopontin	206-216	6.0	70.2	2	78.9
DLNAPSDWDSRGK	P10451	Osteopontin	210-222	5.4	130.7	1	52.6
ETSQLDDQSAETHSHKQS	P10451	Osteopontin	226-243	5.6	101.9	1	57.5
ETSQLDDQSAETHSHKQSRLY	P10451	Osteopontin	226-246	5.4	113.5	1	135.7
DSGSSEEKQLYNKYPDAVAT	P10451	Osteopontin	23-42	6.1	163.9	1	207.3
DDQSAETHSHKQSRLY	P10451	Osteopontin	231-246	6.0	111.3	2	179.5
DQSAETHSHKQSRLY	P10451	Osteopontin	232-246	5.6	76.7	6	133.5
SKEEDKHLKF	P10451	Osteopontin	291-300	5.3	56.8	7	116.8
SKEEDKHLKFRIS	P10451	Osteopontin	291-303	5.7	170.4	11	106.6
FRISHELDSASSEVN	P10451	Osteopontin	300-314	6.0	100.9	3	51.5
RISHELDSASSEVN	P10451	Osteopontin	301-314	5.5	107.6	8	109.2
ISHELDSASSEVN	P10451	Osteopontin	302-314	6.3	71.5	6	199.7
SHELDSASSEVN	P10451	Osteopontin	303-314	6.5	72.1	6	225.7
HELDSASSEVN	P10451	Osteopontin	304-314	5.9	57.6	6	155.4
NKYPDAVAT	P10451	Osteopontin	34-42	5.9	136.2	2	85.2
PSQKQNLLAPQN	P10451	Osteopontin	48-59	5.5	98.0	7	68.9

Supplemental Table S 2: List with all identified peptides, including their precursor protein, sequence range, mean log10 LFQ intensity, inter-individual variation (CV), WGCNA cluster, and identification score. *(continued)*

Sequence	Uniprot ID	Leading protein name	Sequence positions	Mean log10 LFQ intensity	CV (%)	WGCNA cluster	Identification score
YLTQETNKVE	P12272	Parathyroid hormone-related protein	103-112	5.4	135.0	2	112.1
KEQPLKTPGKK	P12272	Parathyroid hormone-related protein	115-125	5.5	104.7	2	78.7
EHQLLHDKGKSIQD	P12272	Parathyroid hormone-related protein	40-53	5.4	82.2	8	73.9
ATSEVSPNSKPSPNTK	P12272	Parathyroid hormone-related protein	74-89	5.5	81.8	8	72.9
TSEVSPNSKPSPNTK	P12272	Parathyroid hormone-related protein	75-89	4.9	66.8	2	71.0
NAKGAVTGAKDAVTTTVT	Q99541	Perilipin-2	107-124	5.9	79.4	5	164.0
GAVTGAKDAVTTTVT	Q99541	Perilipin-2	110-124	5.5	81.3	10	24.9
GAKDAVTTTVT	Q99541	Perilipin-2	114-124	5.5	103.6	8	116.3
GAKDSVASTITGVMD	Q99541	Perilipin-2	125-139	5.4	71.9	10	110.7
KTKGAVTGSVEKTKSVVSG	Q99541	Perilipin-2	140-158	5.6	123.6	1	137.9
SVEKTKSVVSG	Q99541	Perilipin-2	148-158	5.8	76.2	5	138.2
SGVENALTKSELL	Q99541	Perilipin-2	174-186	6.1	64.4	5	181.7
SGVENALTKSELLVEQ	Q99541	Perilipin-2	174-189	6.3	62.1	5	212.2
SGVENALTKSELLVEQY	Q99541	Perilipin-2	174-190	5.8	79.9	5	210.1
GVENALTKSELL	Q99541	Perilipin-2	175-186	5.8	78.7	5	121.4
GVENALTKSELLVEQ	Q99541	Perilipin-2	175-189	5.6	62.8	10	86.3
LPLTEEELEKEAKKVEGFDLV	Q99541	Perilipin-2	191-211	5.6	97.1	5	59.3
EELEKEAKKVE	Q99541	Perilipin-2	196-206	5.7	73.7	5	100.6
EELEKEAKKVEGFDLV	Q99541	Perilipin-2	196-211	6.7	99.6	5	108.6
ASVAVDPQPSVV	Q99541	Perilipin-2	2-13	6.1	116.4	2	163.9
ASVAVDPQPSVVT	Q99541	Perilipin-2	2-14	6.3	49.7	5	185.1
ASVAVDPQPSVVTR	Q99541	Perilipin-2	2-15	6.8	42.5	5	113.5
ASVAVDPQPSVVTRVV	Q99541	Perilipin-2	2-17	6.0	102.7	2	262.4

Supplemental Table S 2: List with all identified peptides, including their precursor protein, sequence range, mean log10 LFQ intensity, inter-individual variation (CV), WGCNA cluster, and identification score. *(continued)*

Sequence	Uniprot ID	Leading protein name	Sequence positions	Mean log10 LFQ intensity	CV (%)	WGCNA cluster	Identification score
ASVAVDPQPSVVTRVVNLPLV	Q99541	Perilipin-2	2-22	6.2	62.3	5	106.9
ASVAVDPQPSVVTRVVNLPLVS	Q99541	Perilipin-2	2-23	7.1	49.1	5	110.4
SLSTKLHSRAYQQALS	Q99541	Perilipin-2	222-237	6.6	120.0	10	124.3
RVKEAKQKSQQTIS	Q99541	Perilipin-2	238-251	5.9	120.2	5	151.5
RVKEAKQKSQQTISQ	Q99541	Perilipin-2	238-252	4.9	120.4	10	45.9
RVKEAKQKSQQTISQLH	Q99541	Perilipin-2	238-254	6.6	130.2	5	230.3
LVGPFYPQLTESQNAQD	Q99541	Perilipin-2	401-417	6.3	105.1	6	59.9
LVGPFYPQLTESQNAQDQ	Q99541	Perilipin-2	401-418	5.9	157.8	7	42.8
DQGAEMDKSSQETQR	Q99541	Perilipin-2	417-431	5.0	100.1	10	45.9
DQGAEMDKSSQETQRSEHKTH	Q99541	Perilipin-2	417-437	5.7	109.8	4	100.7
AEMDKSSQETQRSEHKTH	Q99541	Perilipin-2	420-437	6.5	146.4	5	176.9
EMDKSSQETQRSEHKTH	Q99541	Perilipin-2	421-437	5.7	109.0	5	90.6
AENGVKTITS	Q99541	Perilipin-2	50-59	5.4	99.9	5	103.7
VDPQPSVVTRVVN	Q99541	Perilipin-2	6-18	5.7	85.4	10	93.3
ALPIIQKLEPQIA	Q99541	Perilipin-2	65-77	6.3	44.1	5	139.9
ALPIIQKLEPQIAVANTY	Q99541	Perilipin-2	65-82	5.7	84.5	5	143.9
LPIIQKLEPQIA	Q99541	Perilipin-2	66-77	6.4	57.5	5	199.4
NQPSTQIVANAKGAVT	Q99541	Perilipin-2	98-113	6.0	59.4	10	159.5
QPSTQIVANAKGAVT	Q99541	Perilipin-2	99-113	6.0	51.6	5	66.5
ADTKELVSSKVSGAQEMVS	O60664	Perilipin-3	119-137	5.5	85.0	5	55.0
DTKELVSSKVSGAQ	O60664	Perilipin-3	120-133	5.1	103.7	2	56.5
GVDKTKSVVTG	O60664	Perilipin-3	161-171	5.3	76.0	5	74.8
VGPFAPGITEKAPEEK	O60664	Perilipin-3	418-433	6.1	85.2	10	54.0
VGPFAPGITEKAPEEKK	O60664	Perilipin-3	418-434	6.1	100.5	5	53.2
GAQPILSKLEPQIA	O60664	Perilipin-3	77-90	5.6	68.7	5	101.9
AQPILSKLEPQIA	O60664	Perilipin-3	78-90	5.9	53.7	5	119.4
QPILSKLEPQIA	O60664	Perilipin-3	79-90	5.9	63.2	5	98.0
GPPEEAEDRFSMPLIIT	O00592	Podocalyxin	449-465	5.7	117.8	3	116.5
LTKDDLDEEEDTHL	O00592	Podocalyxin	545-558	5.9	84.8	4	159.1
TKDDLDEEEDTHL	O00592	Podocalyxin	546-558	6.0	86.5	4	247.3
DDLDEEEDTHL	O00592	Podocalyxin	548-558	5.6	153.4	9	169.1

Supplemental Table S 2: List with all identified peptides, including their precursor protein, sequence range, mean log10 LFQ intensity, inter-individual variation (CV), WGCNA cluster, and identification score. *(continued)*

Sequence	Uniprot ID	Leading protein name	Sequence positions	Mean log10 LFQ intensity	CV (%)	WGCNA cluster	Identification score
ADAAPDEKVLDSGFREIENK	P01833	Polymeric immunoglobulin receptor	578-597	6.3	66.8	2	119.4
DAAPDEKVLDSGFREIEN	P01833	Polymeric immunoglobulin receptor	579-596	5.7	88.6	9	97.6
DAAPDEKVLDSGFREIENK	P01833	Polymeric immunoglobulin receptor	579-597	6.7	72.2	9	145.9
EIENKAIQDPRLFAEEK	P01833	Polymeric immunoglobulin receptor	593-609	6.6	53.2	4	74.0
ENKAIQDPRLFAEEK	P01833	Polymeric immunoglobulin receptor	595-609	5.7	52.7	4	49.3
AIQDPRLF	P01833	Polymeric immunoglobulin receptor	598-605	6.1	70.0	3	133.1
AIQDPRLFAEEK	P01833	Polymeric immunoglobulin receptor	598-609	6.7	73.3	3	79.2
AIQDPRLFAEEKAVADT	P01833	Polymeric immunoglobulin receptor	598-614	5.8	80.9	2	75.5
AIQDPRLFAEEKAVADTR	P01833	Polymeric immunoglobulin receptor	598-615	6.7	52.6	9	155.1
AIQDPRLFAEEKAVADTRDQ	P01833	Polymeric immunoglobulin receptor	598-617	6.2	62.9	4	149.7
AIQDPRLFAEEKAVADTRDQADGS	P01833	Polymeric immunoglobulin receptor	598-621	7.0	56.9	9	159.7

Supplemental Table S 2: List with all identified peptides, including their precursor protein, sequence range, mean log10 LFQ intensity, inter-individual variation (CV), WGCNA cluster, and identification score. *(continued)*

Sequence	Uniprot ID	Leading protein name	Sequence positions	Mean log10 LFQ intensity	CV (%)	WGCNA cluster	Identification score
AIQDPRLFAEEKAVADTRDQADGSR	P01833	Polymeric immunoglobulin receptor	598-622	6.9	43.7	9	74.1
QDPRLFAEEKAVADTR	P01833	Polymeric immunoglobulin receptor	600-615	6.1	78.9	2	114.6
DPRLFAEEKAVAD	P01833	Polymeric immunoglobulin receptor	601-613	6.3	51.1	9	117.1
DPRLFAEEKAVADTR	P01833	Polymeric immunoglobulin receptor	601-615	6.6	73.1	9	101.5
DPRLFAEEKAVADTRDQ	P01833	Polymeric immunoglobulin receptor	601-617	6.3	120.9	9	64.5
PRLFAEEKAVADTR	P01833	Polymeric immunoglobulin receptor	602-615	5.5	90.3	2	76.6
LFAEEKAVADTRDQA	P01833	Polymeric immunoglobulin receptor	604-618	5.8	110.8	2	59.2
LFAEEKAVADTRDQAD	P01833	Polymeric immunoglobulin receptor	604-619	5.5	49.4	4	58.9
LFAEEKAVADTRDQADG	P01833	Polymeric immunoglobulin receptor	604-620	5.5	56.1	2	100.1
LFAEEKAVADTRDQADGS	P01833	Polymeric immunoglobulin receptor	604-621	5.9	106.9	9	88.4
LFAEEKAVADTRDQADGSR	P01833	Polymeric immunoglobulin receptor	604-622	6.7	46.9	4	160.1

Supplemental Table S 2: List with all identified peptides, including their precursor protein, sequence range, mean log10 LFQ intensity, inter-individual variation (CV), WGCNA cluster, and identification score. *(continued)*

Sequence	Uniprot ID	Leading protein name	Sequence positions	Mean log10 LFQ intensity	CV (%)	WGCNA cluster	Identification score
FAEEKAVADTRD	P01833	Polymeric immunoglobulin receptor	605-616	5.7	62.1	9	107.1
FAEEKAVADTRDQA	P01833	Polymeric immunoglobulin receptor	605-618	6.1	61.1	9	144.3
FAEEKAVADTRDQAD	P01833	Polymeric immunoglobulin receptor	605-619	5.8	87.9	5	152.9
FAEEKAVADTRDQADG	P01833	Polymeric immunoglobulin receptor	605-620	5.9	66.4	10	81.3
FAEEKAVADTRDQADGS	P01833	Polymeric immunoglobulin receptor	605-621	6.0	137.9	9	200.2
FAEEKAVADTRDQADGSR	P01833	Polymeric immunoglobulin receptor	605-622	6.9	73.8	4	189.6
FAEEKAVADTRDQADGSRASV	P01833	Polymeric immunoglobulin receptor	605-625	5.5	82.6	2	87.8
FAEEKAVADTRDQADGSRASVD	P01833	Polymeric immunoglobulin receptor	605-626	5.6	61.7	5	53.6
AEEKAVADTRDQA	P01833	Polymeric immunoglobulin receptor	606-618	5.8	125.9	9	154.3
AEEKAVADTRDQADGS	P01833	Polymeric immunoglobulin receptor	606-621	5.6	106.6	2	150.2
AEEKAVADTRDQADGSR	P01833	Polymeric immunoglobulin receptor	606-622	6.6	93.8	4	157.1

Supplemental Table S 2: List with all identified peptides, including their precursor protein, sequence range, mean log10 LFQ intensity, inter-individual variation (CV), WGCNA cluster, and identification score. *(continued)*

Sequence	Uniprot ID	Leading protein name	Sequence positions	Mean log10 LFQ intensity	CV (%)	WGCNA cluster	Identification score
EEKAVADTRDQ	P01833	Polymeric immunoglobulin receptor	607-617	5.0	67.8	4	121.8
EEKAVADTRDQA	P01833	Polymeric immunoglobulin receptor	607-618	5.8	124.1	4	144.0
EEKAVADTRDQADG	P01833	Polymeric immunoglobulin receptor	607-620	6.7	69.5	4	225.2
EEKAVADTRDQADGS	P01833	Polymeric immunoglobulin receptor	607-621	6.0	64.1	9	187.4
EEKAVADTRDQADGSR	P01833	Polymeric immunoglobulin receptor	607-622	6.7	49.1	11	103.5
EKAVADTRDQADG	P01833	Polymeric immunoglobulin receptor	608-620	6.6	50.8	11	243.8
EKAVADTRDQADGSR	P01833	Polymeric immunoglobulin receptor	608-622	5.7	72.4	11	51.6
AVADTRDQAD	P01833	Polymeric immunoglobulin receptor	610-619	6.0	64.6	11	177.1
AVADTRDQADG	P01833	Polymeric immunoglobulin receptor	610-620	6.4	72.9	4	204.3
AVADTRDQADGS	P01833	Polymeric immunoglobulin receptor	610-621	6.2	87.7	4	209.8
AVADTRDQADGSR	P01833	Polymeric immunoglobulin receptor	610-622	5.7	150.2	1	94.2

Supplemental Table S 2: List with all identified peptides, including their precursor protein, sequence range, mean log10 LFQ intensity, inter-individual variation (CV), WGCNA cluster, and identification score. *(continued)*

Sequence	Uniprot ID	Leading protein name	Sequence positions	Mean log10 LFQ intensity	CV (%)	WGCNA cluster	Identification score
AVADTRDQADGSRA	P01833	Polymeric immunoglobulin receptor	610-623	5.9	62.0	8	117.9
AVADTRDQADGSRAS	P01833	Polymeric immunoglobulin receptor	610-624	6.4	84.3	10	144.7
AVADTRDQADGSRASV	P01833	Polymeric immunoglobulin receptor	610-625	5.8	131.5	2	120.7
AVADTRDQADGSRASVD	P01833	Polymeric immunoglobulin receptor	610-626	6.5	51.2	4	177.7
AVADTRDQADGSRASVDS	P01833	Polymeric immunoglobulin receptor	610-627	5.6	69.2	8	104.4
AVADTRDQADGSRASVDSG	P01833	Polymeric immunoglobulin receptor	610-628	6.7	58.7	8	253.3
AVADTRDQADGSRASVDSGS	P01833	Polymeric immunoglobulin receptor	610-629	5.4	86.3	8	68.1
AVADTRDQADGSRASVDSGSSE	P01833	Polymeric immunoglobulin receptor	610-631	5.6	58.2	8	85.1
AVADTRDQADGSRASVDSGSSEE	P01833	Polymeric immunoglobulin receptor	610-632	6.1	57.5	9	161.7
AVADTRDQADGSRASVDSGSSEEQ	P01833	Polymeric immunoglobulin receptor	610-633	5.8	51.7	8	138.3
AVADTRDQADGSRASVDSGSSEEQG	P01833	Polymeric immunoglobulin receptor	610-634	6.2	55.1	8	169.6

Supplemental Table S 2: List with all identified peptides, including their precursor protein, sequence range, mean log10 LFQ intensity, inter-individual variation (CV), WGCNA cluster, and identification score. *(continued)*

Sequence	Uniprot ID	Leading protein name	Sequence positions	Mean log10 LFQ intensity	CV (%)	WGCNA cluster	Identification score
VADTRDQADGSRA	P01833	Polymeric immunoglobulin receptor	611-623	5.6	160.1	5	95.0
VADTRDQADGSRAS	P01833	Polymeric immunoglobulin receptor	611-624	6.1	81.8	11	145.8
VADTRDQADGSRASV	P01833	Polymeric immunoglobulin receptor	611-625	5.2	77.9	2	92.9
VADTRDQADGSRASVD	P01833	Polymeric immunoglobulin receptor	611-626	5.7	50.9	9	126.6
VADTRDQADGSRASVDSG	P01833	Polymeric immunoglobulin receptor	611-628	5.8	57.6	8	103.5
VADTRDQADGSRASVDSGSSEE	P01833	Polymeric immunoglobulin receptor	611-632	5.7	128.8	9	163.4
VADTRDQADGSRASVDSGSSEEQG	P01833	Polymeric immunoglobulin receptor	611-634	5.7	72.3	8	95.7
VADTRDQADGSRASVDSGSSEEQGG	P01833	Polymeric immunoglobulin receptor	611-635	5.7	58.4	8	145.1
ADTRDQADGSRASV	P01833	Polymeric immunoglobulin receptor	612-625	5.1	73.7	2	69.7
DTRDQADGSRASVDSGSSE	P01833	Polymeric immunoglobulin receptor	613-631	5.6	51.2	9	136.3
DTRDQADGSRASVDSGSSEEQGGSS	P01833	Polymeric immunoglobulin receptor	613-637	6.3	130.5	9	259.4

Supplemental Table S 2: List with all identified peptides, including their precursor protein, sequence range, mean log10 LFQ intensity, inter-individual variation (CV), WGCNA cluster, and identification score. *(continued)*

Sequence	Uniprot ID	Leading protein name	Sequence positions	Mean log10 LFQ intensity	CV (%)	WGCNA cluster	Identification score
TRDQADGSRASVDSGSSEEQGGSS	P01833	Polymeric immunoglobulin receptor	614-637	5.4	123.8	1	134.8
DQADGSRASVD	P01833	Polymeric immunoglobulin receptor	616-626	5.8	56.6	11	150.0
DQADGSRASVDSG	P01833	Polymeric immunoglobulin receptor	616-628	5.9	74.1	5	239.0
DQADGSRASVDSGSSE	P01833	Polymeric immunoglobulin receptor	616-631	5.4	54.0	5	141.5
DQADGSRASVDSGSSEE	P01833	Polymeric immunoglobulin receptor	616-632	5.7	68.9	9	297.2
DQADGSRASVDSGSSEEQ	P01833	Polymeric immunoglobulin receptor	616-633	5.4	39.2	8	68.8
DQADGSRASVDSGSSEEQG	P01833	Polymeric immunoglobulin receptor	616-634	5.8	50.4	8	187.9
DQADGSRASVDSGSSEEQGG	P01833	Polymeric immunoglobulin receptor	616-635	5.8	48.5	8	244.4
DQADGSRASVDSGSSEEQGGSS	P01833	Polymeric immunoglobulin receptor	616-637	6.9	88.4	9	409.7
DQADGSRASVDSGSSEEQGGSSR	P01833	Polymeric immunoglobulin receptor	616-638	6.7	59.9	11	384.8
DQADGSRASVDSGSSEEQGGSSRA	P01833	Polymeric immunoglobulin receptor	616-639	7.5	57.9	8	374.3

Supplemental Table S 2: List with all identified peptides, including their precursor protein, sequence range, mean log10 LFQ intensity, inter-individual variation (CV), WGCNA cluster, and identification score. *(continued)*

Sequence	Uniprot ID	Leading protein name	Sequence positions	Mean log10 LFQ intensity	CV (%)	WGCNA cluster	Identification score
DQADGSRASVDSGSSEEQGGSSRAL	P01833	Polymeric immunoglobulin receptor	616-640	5.9	44.1	4	235.6
QADGSRASVDSGSSEEQGGSS	P01833	Polymeric immunoglobulin receptor	617-637	5.4	110.8	9	240.2
QADGSRASVDSGSSEEQGGSSRA	P01833	Polymeric immunoglobulin receptor	617-639	5.9	51.2	4	213.7
ADGSRASVDSGSSEEQGGSS	P01833	Polymeric immunoglobulin receptor	618-637	5.6	96.8	4	280.7
ADGSRASVDSGSSEEQGGSSR	P01833	Polymeric immunoglobulin receptor	618-638	5.2	125.3	4	56.1
ADGSRASVDSGSSEEQGGSSRA	P01833	Polymeric immunoglobulin receptor	618-639	5.7	69.2	4	176.8
DGSRASVDSGSSEE	P01833	Polymeric immunoglobulin receptor	619-632	4.8	106.9	4	142.1
DGSRASVDSGSSEEQGGSS	P01833	Polymeric immunoglobulin receptor	619-637	6.1	86.1	9	323.0
DGSRASVDSGSSEEQGGSSR	P01833	Polymeric immunoglobulin receptor	619-638	5.6	112.4	5	147.9
DGSRASVDSGSSEEQGGSSRA	P01833	Polymeric immunoglobulin receptor	619-639	6.5	79.9	9	226.7
DGSRASVDSGSSEEQGGSSRALVST	P01833	Polymeric immunoglobulin receptor	619-643	5.8	81.1	9	53.7

Supplemental Table S 2: List with all identified peptides, including their precursor protein, sequence range, mean log10 LFQ intensity, inter-individual variation (CV), WGCNA cluster, and identification score. *(continued)*

Sequence	Uniprot ID	Leading protein name	Sequence positions	Mean log10 LFQ intensity	CV (%)	WGCNA cluster	Identification score
GSRASVDSGSSEEQGGSS	P01833	Polymeric immunoglobulin receptor	620-637	5.1	78.7	3	124.4
GSRASVDSGSSEEQGGSSRA	P01833	Polymeric immunoglobulin receptor	620-639	5.9	48.6	11	156.9
SRASVDSGSSEEQGGSS	P01833	Polymeric immunoglobulin receptor	621-637	5.4	111.6	4	185.6
SRASVDSGSSEEQGGSSRA	P01833	Polymeric immunoglobulin receptor	621-639	5.7	53.5	8	131.1
ASVDSGSSEEQGGSSRA	P01833	Polymeric immunoglobulin receptor	623-639	6.1	65.3	4	348.2
ASVDSGSSEEQGGSSRALVST	P01833	Polymeric immunoglobulin receptor	623-643	5.6	86.8	4	113.7
SVDSGSSEEQGGSSR	P01833	Polymeric immunoglobulin receptor	624-638	5.5	66.2	4	133.3
SVDSGSSEEQGGSSRA	P01833	Polymeric immunoglobulin receptor	624-639	6.7	47.6	11	338.8
SVDSGSSEEQGGSSRAL	P01833	Polymeric immunoglobulin receptor	624-640	5.1	98.3	4	156.0
SVDSGSSEEQGGSSRALV	P01833	Polymeric immunoglobulin receptor	624-641	5.3	67.9	4	145.1
SVDSGSSEEQGGSSRALVST	P01833	Polymeric immunoglobulin receptor	624-643	6.0	67.6	4	136.3

Supplemental Table S 2: List with all identified peptides, including their precursor protein, sequence range, mean log10 LFQ intensity, inter-individual variation (CV), WGCNA cluster, and identification score. *(continued)*

Sequence	Uniprot ID	Leading protein name	Sequence positions	Mean log10 LFQ intensity	CV (%)	WGCNA cluster	Identification score
SVDSGSSEEQGGSSRALVSTLVPLG	P01833	Polymeric immunoglobulin receptor	624-648	7.5	58.6	8	78.3
VDSGSSEEQGGSSR	P01833	Polymeric immunoglobulin receptor	625-638	5.3	77.3	11	119.8
VDSGSSEEQGGSSRA	P01833	Polymeric immunoglobulin receptor	625-639	6.6	75.0	11	299.2
VDSGSSEEQGGSSRALVST	P01833	Polymeric immunoglobulin receptor	625-643	5.6	68.1	2	68.8
VDSGSSEEQGGSSRALVSTLVPLG	P01833	Polymeric immunoglobulin receptor	625-648	6.6	57.3	8	63.1
DSGSSEEQGGSSRA	P01833	Polymeric immunoglobulin receptor	626-639	6.7	67.8	11	340.9
DSGSSEEQGGSSRAL	P01833	Polymeric immunoglobulin receptor	626-640	5.8	49.6	4	200.8
DSGSSEEQGGSSRALV	P01833	Polymeric immunoglobulin receptor	626-641	6.5	62.0	4	233.4
DSGSSEEQGGSSRALVST	P01833	Polymeric immunoglobulin receptor	626-643	6.5	61.4	11	188.0
DSGSSEEQGGSSRALVSTLVPL	P01833	Polymeric immunoglobulin receptor	626-647	6.7	82.9	1	68.8
DSGSSEEQGGSSRALVSTLVPLG	P01833	Polymeric immunoglobulin receptor	626-648	7.1	108.5	4	125.5

Supplemental Table S 2: List with all identified peptides, including their precursor protein, sequence range, mean log10 LFQ intensity, inter-individual variation (CV), WGCNA cluster, and identification score. *(continued)*

Sequence	Uniprot ID	Leading protein name	Sequence positions	Mean log10 LFQ intensity	CV (%)	WGCNA cluster	Identification score
SGSSEEQGGSSRALV	P01833	Polymeric immunoglobulin receptor	627-641	5.6	77.3	4	160.2
SGSSEEQGGSSRALVSTLVPLG	P01833	Polymeric immunoglobulin receptor	627-648	6.3	95.8	5	92.1
GSSEEQGGSSRALV	P01833	Polymeric immunoglobulin receptor	628-641	5.6	54.5	4	178.7
SSEEQGGSSRALVSTLVPLG	P01833	Polymeric immunoglobulin receptor	629-648	6.0	64.0	5	113.7
EQGGSSRALVSTLVPLG	P01833	Polymeric immunoglobulin receptor	632-648	5.7	101.5	9	109.2
QGGSSRALVSTLVPLG	P01833	Polymeric immunoglobulin receptor	633-648	6.0	78.3	4	110.9
GGSSRALVSTLVPLG	P01833	Polymeric immunoglobulin receptor	634-648	6.2	66.3	4	125.7
GSSRALVSTLVPLG	P01833	Polymeric immunoglobulin receptor	635-648	6.5	60.1	8	114.8
SSRALVSTLVPLG	P01833	Polymeric immunoglobulin receptor	636-648	6.3	76.5	8	109.4
SRALVSTLVPLG	P01833	Polymeric immunoglobulin receptor	637-648	5.5	72.1	8	66.4
SSTVAAEAQDGPQEA	P01833	Polymeric immunoglobulin receptor	750-764	5.5	77.6	5	130.0

Supplemental Table S 2: List with all identified peptides, including their precursor protein, sequence range, mean log10 LFQ intensity, inter-individual variation (CV), WGCNA cluster, and identification score. *(continued)*

Sequence	Uniprot ID	Leading protein name	Sequence positions	Mean log10 LFQ intensity	CV (%)	WGCNA cluster	Identification score
STVAAEAQDGPQEA	P01833	Polymeric immunoglobulin receptor	751-764	5.2	93.3	4	58.7
AVDKKAAGAGKVT	Q5VTE0	Putative elongation factor 1-alpha-like 3	440-452	5.4	75.7	4	76.1
AAGAGKVTKSAQKAQKA	Q5VTE0	Putative elongation factor 1-alpha-like 3	445-461	5.7	100.5	5	136.3
ESRAELTSDKDMY	Q58DD4	Syndecan-2	19-31	6.2	155.0	2	172.9
SRAELTSDKDMY	Q58DD4	Syndecan-2	20-31	6.2	160.0	2	167.2
SPVEESEDVSNK	P31431	Syndecan-4	117-128	5.5	63.4	11	208.6
SPVEESEDVSNKVS	P31431	Syndecan-4	117-130	5.4	76.0	4	69.0

Table with all identified metabolites.

Supplemental Table S 3: List with all identified metabolites, including their mean intensity, inter-individual variation (CV), inter-individual variation for secretor and Lewis positive subjects and the integrated NMR region.

Metabolite	Mean intensity	CV (%)	CV (%) Se(+)Le(+)	Integrated region
2'-FL	11	50.6	37.2	5.31-5.284
3'-FL	20	53.0	41.1	1.189-1.165
Acetate	100	15.9	17.0	1.91-1.894
Alanine	207	15.5	16.1	1.473-1.451
Aspartate	9	90.8	89.3	2.828-2.776
Butyrate	575	4.9	4.8	1.582-1.517
Caprylate	31	25.3	24.7	0.868-0.863
Choline	300	47.8	45.4	3.19-3.177
cis-Aconitate	63	13.8	11.2	3.165-3.149
Citrate	993	23.0	20.4	2.554-2.478
CMP	4	59.8	52.2	6.116-6.096
Creatinine	27	27.0	26.7	3.035-3.024
CTP/CDP	8	46.1	36.7	7.968-7.935
Formate	9	36.9	38.7	8.446-8.434
Fucose-alpha-1,3GLcNAC	1	72.2	58.1	5.146-5.128
Fumarate	1	67.4	64.7	6.515-6.499
Galactose	549	36.4	27.3	4.521-4.497
GlcNAc	363	41.0	38.4	2.018-2.008
Glutamic acid	669	26.6	26.9	2.362-2.319
Glutamine	182	29.9	26.8	2.463-2.416
Glycerophosphocholine	696	31.9	31.4	3.218-3.205
Histidine	4	48.9	50.6	7.072-7.046
Lactate	58	16.4	16.0	1.321-1.313
Lacto-N-difucohexaose I	187	30.7	21.9	5.189-5.172
Lacto-N-difucohexaose II	77	19.4	13.8	5.364-5.349
Lactose	97452	12.0	9.5	3.82-3.748
LDFT	23	52.6	44.6	5.451-5.437
Leucine/isoleucine	12	43.7	44.7	0.937-0.929
LNFP I	1	67.2	63.0	8.39-8.358
LNFP III	1	45.4	41.7	5.122-5.104

Supplemental Table S 3: List with all identified metabolites, including their mean intensity, inter-individual variation (CV), inter-individual variation for secretor and Lewis positive subjects and the integrated NMR region. *(continued)*

Metabolite	Mean intensity	CV (%)	CV (%) Se(+)Le(+)	Integrated region
Methionine	54	19.4	18.7	2.144-2.139
N-acetyl-neuraminic acid	352	33.8	31.2	2.045-2.036
N-acetyl sugar	438	42.5	35.2	2.033-2.023
Pantothenate	7	63.9	64.1	0.926-0.919
Phenylalanine	6	44.7	46.7	7.335-7.304
Phosphocholine	674	35.7	36.6	3.205-3.196
Tyrosine	5	45.2	46.8	7.195-7.164
Urea	571	17.2	17.0	5.873-5.63
Uridine	14	20.7	18.9	5.908-5.886
Valine	20	16.2	17.1	1.056-1.035

Listing S1 - Overview with the parameters of the NMR analysis

##TITLE= Parameter file, TopSpin 3.6.1

##JCAMPDX= 5.0

##DATATYPE= Parameter Values

##NPOINTS= 22 $$ modification sequence number

##ORIGIN= Bruker BioSpin GmbH

##OWNER= nmr

$$ 2020-07-08 11:17:21.887 +0200 nmr@AV600U

$$ /opt/nmrdata/path/Milk/10/acqu

$$ process /opt/topspin3.6.1/prog/mod/go4

##$ACQT0= 0

##$AMP= (0..31)

100 100 100 100 100 100 100 100 100 100 100 100 100 100 100 100 100 100

100 100 100 100 100 100 100 100 100 100 100 100 100 100

##$AMPCOIL= (0..19)

-1 0 0 0 0 0 0 0 0 0 0 0 0 0 0 0 0 0 0 0

##$ANAVPT= 1

##$AQSEQ= 0

##$AQ_mod= 3

##$AUNM=

##$AUTOPOS= <1 A1 - 101 >

##$BF1= 600.36

##$BF2= 600.36

##$BF3= 600.36

##$BF4= 600.36

##$BF5= 600.36

##$BF6= 600.36

##$BF7= 600.36

##$BF8= 600.36

##$BWFAC= (0..63)

0 0 0 0 0 0 0 0 0 0 0 0 0 0 0 0 0 0 0 0 0 0 0 0 0 0 0 0 0 0 0 0 0 0 0 0

0 0 0 0 0 0 0 0 0 0 0 0 0 0 0 0 0 0 0 0 0 0 0 0 0 0 0 0

##$BYTORDA= 0

##$CAGPARS= (0..11)

0 0 0 0 0 0 0 0 0 0 0 0

##$CHEMSTR=

##$CNST= (0..63)

1 1 145 1 1 1 1 1 1 1 1 1 1 1 1 1 1 1 1 1 1 1 1 1 1 1 1 1 1 0.75 1 1 1

1 1 1 1 1 1 1 1 1 1 1 1 1 1 1 1 1 1 1 1 1 1 1 1 1 1 1 1 1 1 1

##$CPDPRG= (0..8)

<> <> <> <>

##$D= (0..63)

0 4 0 0 0 0 0 0 0.01 0.06 0 0.03 2e-05 0 0 0 0.0002 0 0 0 0 0 0 0 0 0 0

0 0 0 0 0 0 0 0 0 0 0 0 0 0 0 0 0 0 0 0 0 0 0 0 0 0 0 0 0 0 0 0 0 0 0 0

0

##$DATE= 0

##$DE= 10

##$DECBNUC=

##$DECIM= 1109.33333333333

##$DECNUC=

##$DECSTAT= 4

##$DIGMOD= 3

##$DIGTYP= 12

##$DQDMODE= 0

##$DR= 22

##$DS= 4

##$DSPFIRM= 4

##$DSPFVS= 0

##$DTYPA= 0

##$EXP=

##$FCUCHAN= (0..9)

0 2 0 0 0 0 0 0 0 0

##$FL1= 90

##$FL2= 90

##$FL3= 90

##$FL4= 90

##$FN_INDIRECT= (0..7)

0 2 2 2 2 2 2 2

##$FOV= 20

##$FQ1LIST=

##$FQ2LIST=

##$FQ3LIST=

##$FQ4LIST=

##$FQ5LIST=

##$FQ6LIST=

##$FQ7LIST=

##$FQ8LIST=

##$FRQLO3= 0

##$FRQLO3N= 0

##$FS= (0..7)

83 83 83 83 83 83 83 83

##$FTLPGN= 0

##$FW= 4032000

##$FnILOOP= 0

##$FnMODE= 0

##$FnTYPE= 0

##$GPNAM= (0..31)

<sine.100> <SMSQ10.100> <SMSQ10.100> <sine.100> <sine.100> <sine.100> <sine.100>

<sine.100> <sine.100> <sine.100> <sine.100> <sine.100> <sine.100> <sine.100>

<sine.100> <sine.100> <sine.100> <sine.100> <sine.100> <sine.100> <sine.100>

<sine.100> <sine.100> <sine.100> <sine.100> <sine.100> <sine.100> <sine.100>

<sine.100> <sine.100> <sine.100> <sine.100>

##$GPX= (0..31)

0 0 0 0 0 0 0 0 0 0 0 0 0 0 0 0 0 0 0 0 0 0 0 0 0 0 0 0 0 0 0 0

##$GPY= (0..31)

0 0 0 0 0 0 0 0 0 0 0 0 0 0 0 0 0 0 0 0 0 0 0 0 0 0 0 0 0 0 0 0

##$GPZ= (0..31)

0 50 -10 0 0 0 0 0 0 0 0 0 0 0 0 0 0 0 0 0 0 0 0 0 0 0 0 0 0 0 0 0

##$GRDPROG= <>

##$GRPDLY= -1

##$HDDUTY= 20

##$HDRATE= 20

##$HGAIN= (0..3)

0 0 0 0

##$HL1= 1

##$HL2= 35

##$HL3= 16

##$HL4= 17

##$HOLDER= 101

##$HPMOD= (0..7)

0 0 0 0 0 0 0 0

##$HPPRGN= 0

##$IN= (0..63)

0.001 0.001 0.001 0.001 0.001 0.001 0.001 0.001 0.001 0.001 0.001 0.001

0.001 0.001 0.001 0.001 0.001 0.001 0.001 0.001 0.001 0.001 0.001 0.001

0.001 0.001 0.001 0.001 0.001 0.001 0.001 0.001 0.001 0.001 0.001 0.001

0.001 0.001 0.001 0.001 0.001 0.001 0.001 0.001 0.001 0.001 0.001 0.001

0.001 0.001 0.001 0.001 0.001 0.001 0.001 0.001 0.001 0.001 0.001 0.001

0.001 0.001 0.001 0.001

##$INF= (0..7)

0 0 0 0 0 0 0 0

##$INP= (0..63)

0 0 0 0 0 0 0 0 0 0 0 0 0 0 0 0 0 0 0 0 0 0 0 0 0 0 0 0 0 0 0 0 0 0 0 0

0 0 0 0 0 0 0 0 0 0 0 0 0 0 0 0 0 0 0 0 0 0 0 0 0 0 0 0

##$INSTRUM= <>

##$INTEGFAC= (0..63)

0 0 0 0 0 0 0 0 0 0 0 0 0 0 0 0 0 0 0 0 0 0 0 0 0 0 0 0 0 0 0 0 0 0 0 0

0 0 0 0 0 0 0 0 0 0 0 0 0 0 0 0 0 0 0 0 0 0 0 0 0 0 0 0

##$L= (0..31)

1 1 1 1 1 1 1 1 1 1 1 1 1 1 1 1 1 1 1 1 1 1 1 1 1 1 1 1 1 1 1 1

##$LFILTER= 10

##$LGAIN= -10

##$LINPSTP= 0

##$LOCKED= no

##$LOCKFLD= 0

##$LOCKGN= 0

##$LOCKPOW= -20

##$LOCKPPM= 0

##$LOCNUC= <2H>

##$LOCPHAS= 0

##$LOCSHFT= no

##$LOCSW= 0

##$LTIME= 0.1

##$MASR= 4200

##$MASRLST=

##$MULEXPNO= (0..15)

0 0 0 0 0 0 0 0 0 0 0 0 0 0 0 0

##$NBL= 1

##$NC= 0

##$NLOGCH= 2

##$NOVFLW= 0

##$NS= 192

##$NUC1= <1H>

##$NUC2=

##$NUC3=

##$NUC4=

##$NUC5=

##$NUC6=

##$NUC7=

##$NUC8=

##$NUCLEUS=

##$NUSLIST=

##$NusAMOUNT= 25

##$NusFPNZ= no

##$NusJSP= 0

##$NusSEED= 54321

##$NusSPTYPE= 0

##$NusT2= 1

##$NusTD= 0

##$O1= 2822.737

##$O2= 2822.737

##$O3= 2822.737

##$O4= 2822.737

##$O5= 2822.737

##$O6= 2822.737

##$O7= 2822.737

##$O8= 2822.737

##$OVERFLW= 0

##$P= (0..63)

7.2 7.2 14.4 0 0 16.5 25 50 20000 0 0 80000 80000 1000 0 200000 1000 2500

100000 600 2500 0 0 0 0 180 100000 7.2 0 1000 0 0 20000 0 0 0 0 0 0 0 2000

0 0 0 200 0 80000 46600 10000 0 80000 0 0 0 0 0 0 0 0 0 0 0 0 0

##$PACOIL= (0..15)

1 0 4 2 3 0 0 0 0 0 0 0 0 0 0 0

##$PAPS= 2

##$PARMODE= 0

##$PCPD= (0..9)

100 70 100 100 100 100 100 100 100 100

##$PEXSEL= (0..9)

1 1 1 1 1 1 1 1 1 1

##$PHCOR= (0..31)

0 0 0 0 0 0 0 0 0 0 0 0 0 0 0 0 0 0 0 0 0 0 0 0 0 0 0 0 0 0 0 0

##$PHLIST= <>

##$PHP= 1

##$PH_ref= 0

##$PL= (0..63)

120 6.5 120 120 120 120 120 120 120 60 15.18248 28.17752 120 120 120 120

120 120 6.5 21.80235 120 120 120 120 120 120 120 28.17752 120 120 120 120

120 120 120 120 120 120 120 120 120 120 120 120 120 120 120 120 120 120

120 120 120 120 120 120 120 120 120 120 120 120 120 120

##$PLSTEP= 0.1

##$PLSTRT= -6

##$PLW= (0..63)

0 4 0 0 0 0 0 0 0 8.3058e-06 0.33223 0.025635 0 0 0 0 0 0 4 0.042377 0

0 0 0 0 0 0 0.025635 0 0.33223 0 0 3.3223e-07 0 0 0 0 0 0 0 0 0 0 0 0 0

0 0 0 0 0 0 0 0 0 0 0 0 0 0 0 0 0 0

##$PLWMAX= (0..7)

0 0 0 0 0 0 0 0

##$PQPHASE= 0

##$PQSCALE= 0

##$PR= 1

##$PRECHAN= (0..15)

-1 3 0 2 -1 -1 -1 -1 -1 -1 -1 -1 -1 -1 -1 -1

##$PRGAIN= 0

##$PROBHD= <34>

##$PROBINPUTS= (0..15)

<> <> <> <> <> <> <> <> <> <> <> <> <> <> <> <>

##$PULPROG=

##$PW= 0

##$PYNM= <acqu.py>

##$ProjAngle= 0

##$QNP= 1

##$RD= 0

##$RECCHAN= (0..15)

0 2 0 0 0 0 0 0 0 0 0 0 0 0 0 0

##$RECPH= 0

##$RECPRE= (0..15)

-1 0 -1 -1 -1 -1 -1 -1 -1 -1 -1 -1 -1 -1 -1 -1

##$RECPRFX= (0..15)

1 0 0 0 0 0 0 0 0 0 0 0 0 0 0 0

##$RECSEL= (0..15)

0 0 1 0 0 0 0 0 0 0 0 0 0 0 0 0

##$RG= 32

##$RO= 0

##$RSEL= (0..15)

0 0 2 0 0 0 0 0 0 0 0 0 0 0 0 0

##$S= (0..7)

83 4 83 83 83 83 83 83

##$SELREC= (0..9)

0 0 0 0 0 0 0 0 0 0

##$SFO1= 600.362822737

##$SFO2= 600.362822737

##$SFO3= 600.362822737

##$SFO4= 600.362822737

##$SFO5= 600.362822737

##$SFO6= 600.362822737

##$SFO7= 600.362822737

##$SFO8= 600.362822737

##$SOLVENT= <H2O+D2O>

##$SOLVOLD=

##$SP= (0..63)

1 76.59016 70.56956 120 120 0 66.23932 120 0 0 0 43.22378 0 0 120 0 150

150 120 68.53075 66.62075 69.93075 62.61075 150 150 150 150 150 150 120

120 120 120 120 120 120 120 120 120 120 120 120 120 120 120 120 120 120

120 120 120 120 120 120 120 120 120 120 120 120 120 120 120 120

##$SPECTR= 0

##$SPINCNT= 0

##$SPNAM= (0..63)

<Gaus1_270.1000> <Gaus1_180r.1000> <> <> <Sinc1.1000> <Squa100.1000>

<> <Squa100.1000> <Sinc1.1000> <Sinc1.1000> <>

<> <> <Sinc1.1000> <Sinc1.1000> <Sinc1.1000> <Sinc1.1000>

<Crp60,20,20.10> <Bip720,50,20.1>

<> <> <> <Rsnob.1000> <Gaus1_180r.1000> <Gaus1_180r.1000> <> <> <> <Gaus1_180r.1000>

<Squa100.1000> <> <> <> <> <> <> <> <> <> <> <> <> <> <> <> <> <>

<> <>

<> <> <>

##$SPOAL= (0..63)

0.5 1 0.5 0.5 0.5 0.5 0.5 0.5 0.5 0.5 0.5 0.5 0.5 0.5 0.5 0.5 0.5 0.5 0.5

1 1 1 1 0.5 0.5 0.5 0.5 0.5 0.5 0.5 0.5 0.5 0.5 0.5 0.5 0.5 0.5 0.5 0.5

0.5 0.5 0.5 0.5 0.5 0.5 0.5 0.5 0.5 0.5 0.5 0.5 0.5 0.5 0.5 0.5 0.5 0.5

0.5 0.5 0.5 0.5 0.5 0.5 0.5

##$SPOFFS= (0..63)

0 0 0 0 0 0 0 0 0 0 0 0 0 0 0 0 0 0 0 0 0 0 0 0 0 0 0 0 0 0 0 0 0 0 0 0

0 0 0 0 0 0 0 0 0 0 0 0 0 0 0 0 0 0 0 0 0 0 0 0 0 0 0 0

##$SPPEX= (0..63)

0 0 0 0 0 0 0 0 0 0 0 0 0 0 0 0 0 0 0 0 0 0 0 0 0 0 0 0 0 0 0 0 0 0 0 0

0 0 0 0 0 0 0 0 0 0 0 0 0 0 0 0 0 0 0 0 0 0 0 0 0 0 0 0

##$SPW= (0..63)

0 1.7238e-06 7.6612e-07 0 0 0.00059874 8.3058e-06 0 2.0764e-08 0 0.00059874

0.00059874 0 0 0 0 0 0 0 1.222967e-06 1.901063e-06 8.876617e-07 4.787086e-06

0 0 0 0 0 0 0.0079315 0.33223 0 0 0 8.3756e-06 7.6612e-07 4.9032e-05 0

0 0 7.6612e-07 2.0764e-08 0 0 0 0 0 0 0 0 0 0 0 0 0 0 0 0 0 0 0 0 0 0

##$SUBNAM= (0..9)

<"“> <’%‘> <’%‘> <’%‘> <’%‘> <’%‘> <’%‘> <’%‘> <’%‘> <”">

##$SW= 30.0299176948508

##$SWIBOX= (0..19)

0 0 2 0 0 0 0 0 0 0 0 0 0 0 0 0 0 0 0 0

##$SW_h= 18028.8461538462

##$SWfinal= 0

##$SigLockShift= 0

##$TD= 97352

##$TD0= 1

##$TD_INDIRECT= (0..7)

0 2 2 2 2 2 2 2

##$TDav= 0

##$TE= 300

##$TE1= 300

##$TE2= 300

##$TE3= 300

##$TE4= 300

##$TEG= 300

##$TE_MAGNET= 300

##$TE_PIDX= 0

##$TE_STAB= (0..9)

0 0 0 0 0 0 0 0 0 0

##$TL= (0..7)

0 120 120 120 120 120 120 120

##$TOTROT= (0..63)

0 0 0 0 0 0 0 0 0 0 0 0 0 0 0 0 0 0 0 0 0 0 0 0 0 0 0 0 0 0 0 0 0 0 0 0

0 0 0 0 0 0 0 0 0 0 0 0 0 0 0 0 0 0 0 0 0 0 0 0 0 0 0 0

##$TUBE_TYPE= <>

##$USERA1=

##$USERA2=

##$USERA3=

##$USERA4=

##$USERA5=

##$V9= 5

##$VALIDCODE= 0

##$VALIST=

##$VCLIST=

##$VDLIST=

##$VPLIST=

##$VTLIST=

##$WBST= 1024

##$WBSW= 4

##$XGAIN= (0..3)

0 0 0 0

##$XL= 0

##$YL= 0

##$YMAX_a= 0

##$YMIN_a= 0

##$ZGOPTNS= <-DFLAG_BLK>

##$ZL1= 120

##$ZL2= 120

##$ZL3= 120

##$ZL4= 120

##$scaledByNS= no

##$scaledByRG= no

##END=

References

(1) Hettinga, K.; van Valenberg, H.; de Vries, S.; Boeren, S.; van Hooijdonk, T.; van Arendonk, J.; Vervoort, J. The host defense proteome of human and bovine milk. *PLoS One* **2011**, *6*, e19433.
